# The R2TP complex regulates paramyxovirus RNA synthesis

**DOI:** 10.1371/journal.ppat.1007749

**Published:** 2019-05-23

**Authors:** Hiroshi Katoh, Tsuyoshi Sekizuka, Yuichiro Nakatsu, Reiko Nakagawa, Naganori Nao, Masafumi Sakata, Fumihiro Kato, Makoto Kuroda, Minoru Kidokoro, Makoto Takeda

**Affiliations:** 1 Department of Virology III, National Institute of Infectious Diseases, Tokyo, Japan; 2 Pathogen Genomics Center, National Institute of Infectious Diseases, Tokyo, Japan; 3 Laboratory for Phyloinformatics, Center for Biosystems Dynamics Research, RIKEN, Hyogo, Japan; Boston University, UNITED STATES

## Abstract

The regulation of paramyxovirus RNA synthesis by host proteins is poorly understood. Here, we identified a novel regulation mechanism of paramyxovirus RNA synthesis by the Hsp90 co-chaperone R2TP complex. We showed that the R2TP complex interacted with the paramyxovirus polymerase L protein and that silencing of the R2TP complex led to uncontrolled upregulation of mumps virus (MuV) gene transcription but not genome replication. Regulation by the R2TP complex was critical for MuV replication and evasion of host innate immune responses. The R2TP complex also regulated measles virus (MeV) RNA synthesis, but its function was inhibitory and not beneficial to MeV, as MeV evaded host innate immune responses in the absence of the R2TP complex. The identification of the R2TP complex as a critical host factor sheds new light on the regulation of paramyxovirus RNA synthesis.

## Author summary

The family *Paramyxoviridae* includes several important human and animal pathogens such as mumps virus (MuV) and measles virus (MeV). Paramyxovirus RNA synthesis is strictly regulated by both viral and host proteins. In this study, we identified the R2TP complex as a novel host factor regulating paramyxovirus RNA synthesis. The R2TP complex is a Hsp90 co-chaperone and is involved in Hsp90-mediated assembly of large protein complexes. We showed that the R2TP complex precisely regulated MuV transcription by interacting with the polymerase L protein. This regulation was critical for MuV evasion of host innate immune responses and for viral replication. We also showed that the R2TP complex regulated MeV RNA synthesis, but that its function was inhibitory and not beneficial to MeV. Our findings support a novel regulation mechanism of paramyxovirus RNA synthesis that is directly relevant to its biology and life cycle, and provide the first evidence linking the R2TP complex to defense against viral infection.

## Introduction

Many human and animal pathogens are members of the family *Paramyxoviridae* [1], including mumps rubulavirus (MuV) and measles morbillivirus (MeV). Mumps is a common childhood illness characterized by painful swelling of the parotid glands, and is often accompanied by severe complications such as orchitis, aseptic meningitis, pancreatitis and deafness [2]. Measles often causes a maculopapular skin rash and fever, and is accompanied by cough, coryza and conjunctivitis [3].

Paramyxoviruses have a non-segmented negative-strand RNA genome, 15–19 kb in length [1]. The genome encodes six or seven structural proteins and contains control regions at both genomic termini [4]. In addition to the terminal control regions, transcriptional control sequences exist at the beginning and end of each gene. The viral genome forms ribonucleoprotein (RNP) complexes with the nucleocapsid (N) protein and the RNA-dependent RNA polymerase (RdRp), which is composed of the large (L) protein and the phosphoprotein (P) [1]. The RNP complex, but not the naked genome, functions as an active template for both transcription and genome replication. The L protein exhibits all the major catalytic activities for RNA synthesis (nucleotide polymerization [5], mRNA capping [6] and polyadenylation [7]), while the P protein functions as an essential cofactor for the L protein functions. RdRp initiates transcription from the 3’ end of the genome, and transcribes viral genes in sequential order [8]. Since RdRp may dissociate from the genome at the boundaries between each gene, mRNAs derived from 3’ genes are always more abundant than those of 5’ genes, producing a transcriptional gradient of mRNA abundance [8,9].

Heat shock protein 90 (Hsp90) supports maturation of the paramyxovirus L protein and RdRp complex formation [10–12]. Hsp90 is a ubiquitously-expressed molecular chaperone that plays essential roles in cellular homeostasis and survival [13]. The primary function of Hsp90 is thought to be protein stabilization and activation. In addition, based on recent comprehensive analyses of the physical interaction network of molecular chaperones, Hsp90 appears to have another major role in the assembly of multiprotein complexes by stabilizing unstable protein subunits and facilitating their incorporation into complexes [14,15]. The R2TP complex is one of Hsp90’s co-chaperones and is involved in Hsp90-mediated assembly of large protein or protein-RNA complexes such as RNA polymerase II [16], phosphatidylinositol-3 kinase-related protein kinase [17] and small nuclear and nucleolar ribonucleoproteins [18–22]. The R2TP complex consists of two RuvB-like AAA+ ATPases (RuvBL1 and RuvBL2), PIH1 domain containing 1 (PIH1D1) and RNA polymerase II associated protein 3 (RPAP3) [23]. Although the R2TP complex functions with Hsp90 to facilitate assembly of protein complexes, the molecular mechanisms of its action remain unclear.

Here, using two representative paramyxoviruses, MuV and MeV, we showed that the R2TP complex functions as a regulator and suppressor of paramyxovirus RNA synthesis. This is the first discovery of a host factor exhibiting a regulatory function on paramyxovirus RNA synthesis.

## Results

### R2TP core proteins interact with paramyxovirus L protein

**Fig 1:**
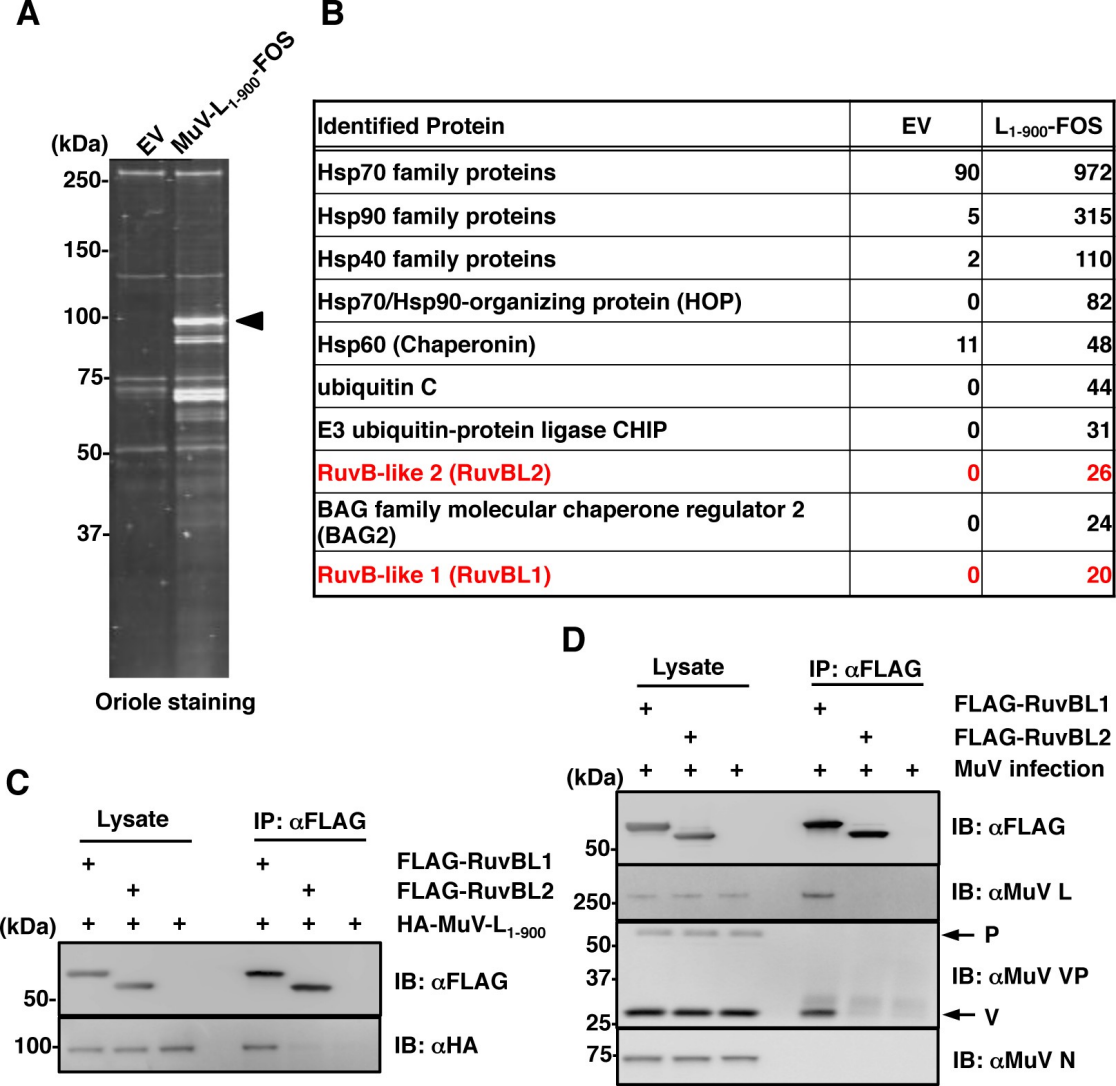
RuvBL1 is a host factor interacting with the MuV L protein. (**A**) Purified proteins were subjected to SDS-PAGE, followed by oriole staining. The empty vector (EV) was used as a negative control. An arrowhead indicates the band corresponding to MuV-L_1-900_-FOS. (**B**) List of proteins identified by mass spectrometry. The numbers indicate the total spectrum counts for each protein family. (**C**) FLAG-RuvBL1 or FLAG-RuvBL2 were co-expressed with HA-MuV-L_1-900_ in 293T cells, immunoprecipitated with anti-FLAG antibody, and immunoblotted with anti-FLAG and anti-HA antibodies. (**D**) 293T cells were infected with MuV at a MOI of 5.0 and transfected with pcDNA-FLAG-RuvBL1 or pcDNA-FLAG-RuvBL2. After 24 h, cell lysates were immunoprecipitated with anti-FLAG antibody and immunoblotted with the indicated antibodies.

We previously showed that MuV L protein interacts with Hsp90 [12]. N-terminally HA-tagged MuV L protein and truncated variants were expressed and immunoprecipitated with anti-HA antibody (S1 Fig). Hsp90 was co-immunoprecipitated with truncated mutants retaining the N-terminal half of MuV L protein, including the 900-residue HA-MuV-L_1-900_. Lesser amounts of Hsp90 were co-immunoprecipitated when shorter N-terminal subsequences of the L protein were used. MuV-L_1-900_-FOS was expressed in 293T cells and purified by FOS affinity chromatography as described previously [24]. SDS-PAGE showed that several host factors were present in the purified complexes (Fig 1A). LC-MS/MS analysis revealed that, in addition to Hsp90, several chaperone and co-chaperone proteins were detected with high peptide coverage (Fig 1B). Among these, we focused on the newly-identified RuvBL1 and RuvBL2 as interaction partners of the paramyxovirus L protein. These proteins are components of the R2TP complex, which functions as a Hsp90 co-chaperone [25].

To confirm the interaction of MuV L protein with RuvBL1 and RuvBL2, HA-MuV-L_1-900_ and FLAG-tagged RuvBL1 or RuvBL2 were co-expressed and immunoprecipitated with anti-FLAG antibody. HA-MuV-L_1-900_ was co-precipitated with FLAG-RuvBL1 (Fig 1C). FLAG-RuvBL1 or FLAG-RuvBL2 was expressed in MuV-infected cells and immunoprecipitated with anti-FLAG antibody. FLAG-RuvBL1 interacted with the untagged native form of the L protein in MuV-infected cells (Fig 1D). Unexpectedly, the V protein was also co-immunoprecipitated with FLAG-RuvBL1. However, these co-immunoprecipitation assays failed to detect the L-RuvBL2 interaction, which was demonstrated using the FOS-tagged purification assay.

**Fig 2:**
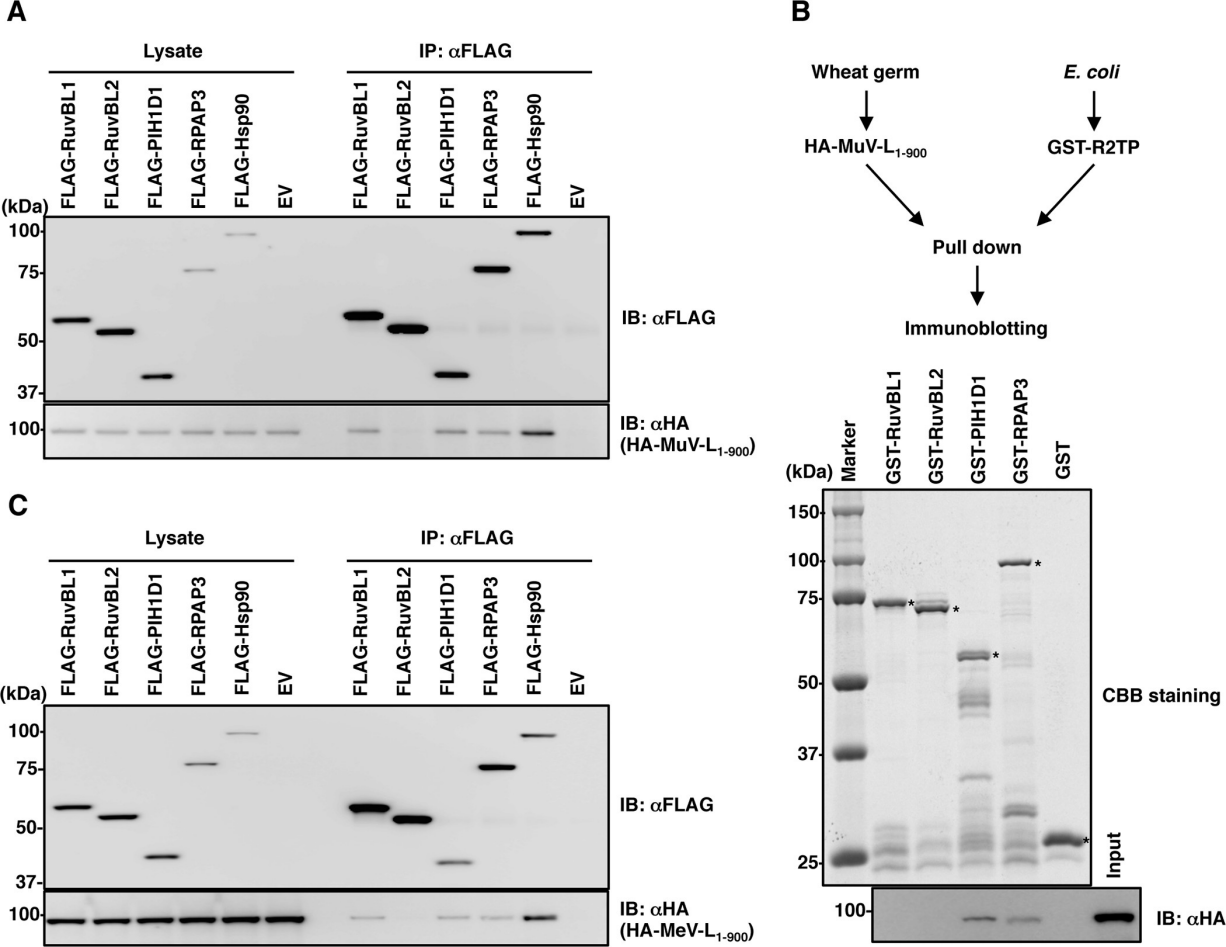
The paramyxoviral L protein interacts with the R2TP complex. (**A, C**) FLAG-RuvBL1, FLAG-RuvBL2, FLAG-PIH1D1, FLAG-RPAP3 or FLAG-Hsp90 were co-expressed with HA-MuV-L_1-900_ (A) or HA-MeV-L_1-900_ (C) in 293T cells, immunoprecipitated with anti-FLAG antibody, and immunoblotted with anti-FLAG and anti-HA antibodies. (**B**) HA-MuV-L_1-900_ and GST-R2TP complex proteins were expressed in a wheat germ cell-free system and in *E*. *coli*, respectively, and then a pull-down assay and immunoblotting were performed. Asterisks indicate each recombinant protein.

Co-immunoprecipitation assays using a mammalian cell expression system further demonstrated the interaction of the MuV L protein with other R2TP core proteins, PIH1D1 and RPAP3 (Fig 2A). HA-MuV-L_1-900_ and glutathione S-transferase (GST)-tagged individual components of the R2TP complex were also expressed independently in a wheat germ cell free system and in *Escherichia coli*, respectively, and a GST-pull-down assay was performed. The GST-pull-down assay confirmed that HA-MuV-L_1-900_ interacted with both PIH1D1 and RPAP3 (Fig 2B). The N-terminal domain of PIH1D1 interacts with several client proteins [26], but HA-MuV-L_1-900_ was not co-immunoprecipitated with the N-terminal domain of PIH1D1 (S2 Fig), suggesting that the interaction between MuV L protein and PIH1D1 was mediated through another recognition mechanism. Similar co-immunoprecipitation analyses were performed using the L protein of another paramyxovirus, MeV. RuvBL1, PIH1D1 and RPAP3 also interacted with the MeV L protein (Fig 2C).

### Silencing of the R2TP complex enhances paramyxoviral RNA synthesis

**Fig 3:**
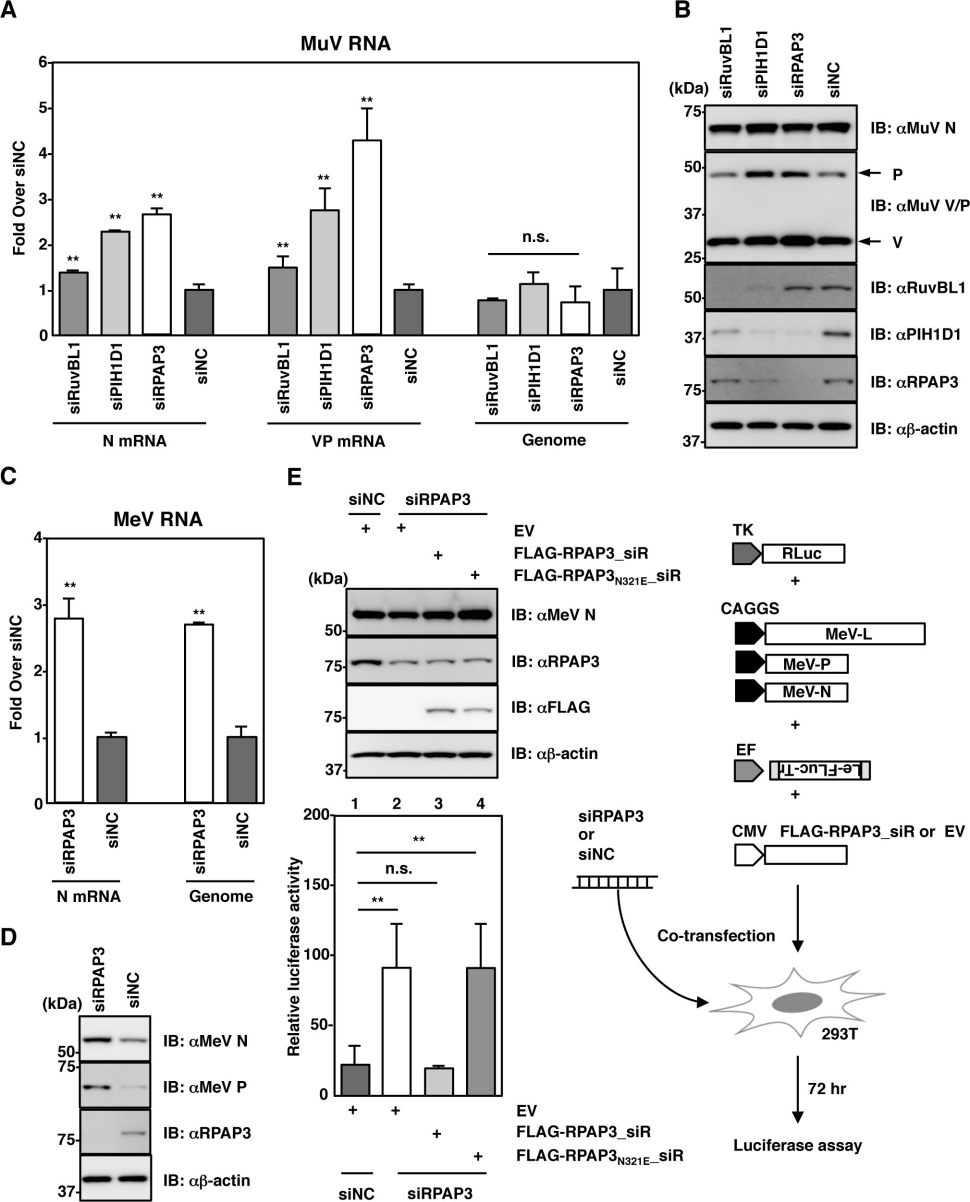
Silencing of the R2TP complex enhances paramyxoviral RNA synthesis. (**A, B**) At 48 h post-transfection with either siRuvBL1, siPIH1D1, siRPAP3 or siNC, A549 cells were infected with rMuV at a MOI of 5.0 and incubated for 24 h. (A) The relative levels of each viral RNA (based on levels in cells transfected with siNC) were determined by qPCR. (B) Cell lysates were subjected to immunoblotting with the indicated antibodies. (**C, D**) At 48 h post-transfection with either siRPAP3 or siNC, A549/hSLAM cells were infected with rMeV/eGFP at a MOI of 1.0 and incubated for 24 h. (C) Relative levels of N mRNA and genomic RNA (based on levels in cells transfected with siNC) were determined by qPCR. (D) Cell lysates were immunoblotted with the indicated antibodies. (**E**) 293T cells were co-transfected with the MeV minigenome plasmid, three support plasmids, pRL-TK, the rescue plasmid and siRNA as indicated in the figure. At 72 h post-transfection, the relative luciferase activity (based on the levels of transfected cells without pCA7-L) was determined. At 72 h post-transfection, cell lysates were immunoblotted with the indicated antibodies. Error bars indicate the standard deviations of triplicate wells. The significance of differences between means was determined using the Student’s *t*-test. **p<0.01. n.s. = not significant.

To investigate the roles of the R2TP complex in paramyxoviral RNA synthesis, expression of the R2TP complex was silenced using small interfering RNAs (siRNAs). Each siRNA efficiently decreased levels of the target molecule (RuvBL1, PIH1D1 or RPAP3). The levels of non-target R2TP components were also reduced by theses siRNAs, likely due to codependence of these components for expression of the R2TP complex (Fig 3B). At 48 h post-siRNA transfection, A549 cells were infected with recombinant MuV (rMuV) at a multiplicity of infection (MOI) of 5.0, and the effect of the siRNAs on viral RNA synthesis at the one-step growth stage of infection was analyzed. At 24 h post-infection (hpi), total RNA was extracted, and levels of viral mRNA and genomic RNA were determined by quantitative reverse transcription-PCR (qRT-PCR). The levels of mRNAs encoding both N and V/P proteins in cells treated with RuvBL1-, PIH1D1- or RPAP3-specific siRNAs were ~1.5- to 4-fold higher than those in cells treated with a control siRNA (siNC) (Fig 3A). By contrast, genomic RNA levels remained unchanged (Fig 3A). The increased expression levels of P and V proteins were evident in cells treated with siPIH1D1 and siRPAP3 (Fig 3B). Since siRPAP3 showed the greatest effect among the siRNAs, further studies were mainly performed using siRPAP3.

MeV RNA synthesis at the one-step stage of viral growth was also enhanced in cells treated with siRPAP3. In the case of MeV, both viral transcription and genome replication were upregulated (Fig 3C and 3D). The intracellular localization of MuV and MeV N proteins in RPAP3-knockdown cells and control cells was analyzed. In concordance with the high levels of viral RNAs and viral proteins in RPAP3-knockdown cells, the size of syncytia was greater in these cells than in control cells (S3 Fig). However, N protein distribution was similar in RPAP3-knockdown and control cells. In both cells, the MuV N protein was mainly distributed in cytoplasmic inclusion bodies, and the MeV N protein was diffusely distributed in the cytoplasm. These data suggested that RPAP3 knockdown did not affect the intracellular transport and localization of the paramyxovirus N protein. To further evaluate the effects of RPAP3 silencing on MeV gene expression, a new MeV minigenome system was established using 293T cells (Fig 3E). Knockdown of RPAP3 increased MeV minigenome expression, as shown by increased luciferase activity (Fig 3E). This increased activity was abrogated by expression of a siRNA-resistant RPAP3 (FLAG-RPAP3_siR, Fig 3E). As expected, abrogation did not occur for siRNA-resistant RPAP3 bearing the N321E mutation (FLAG-RPAP3_N321E__siR, Fig 3E), as this mutation in the TPR2 domain disrupted the interaction between RPAP3 with Hsp90 (S4 Fig). However, one caveat to be noted in interpreting these data is that the anti-RPAP3 antibody failed to detect FLAG-tagged siRNA-resistant RPAP3s (FLAG-RPAP3_siR and FLAG-RPAP3_N321E__siR). Therefore, the expression of FLAG-tagged siRNA-resistant RPAP3s was confirmed using an anti-FLAG antibody. These data suggested that the R2TP complex functions as a suppressor of paramyxoviral RNA synthesis with the support of Hsp90.

### RPAP3 knockdown enhances MeV propagation, but restricts MuV propagation

**Fig 4:**
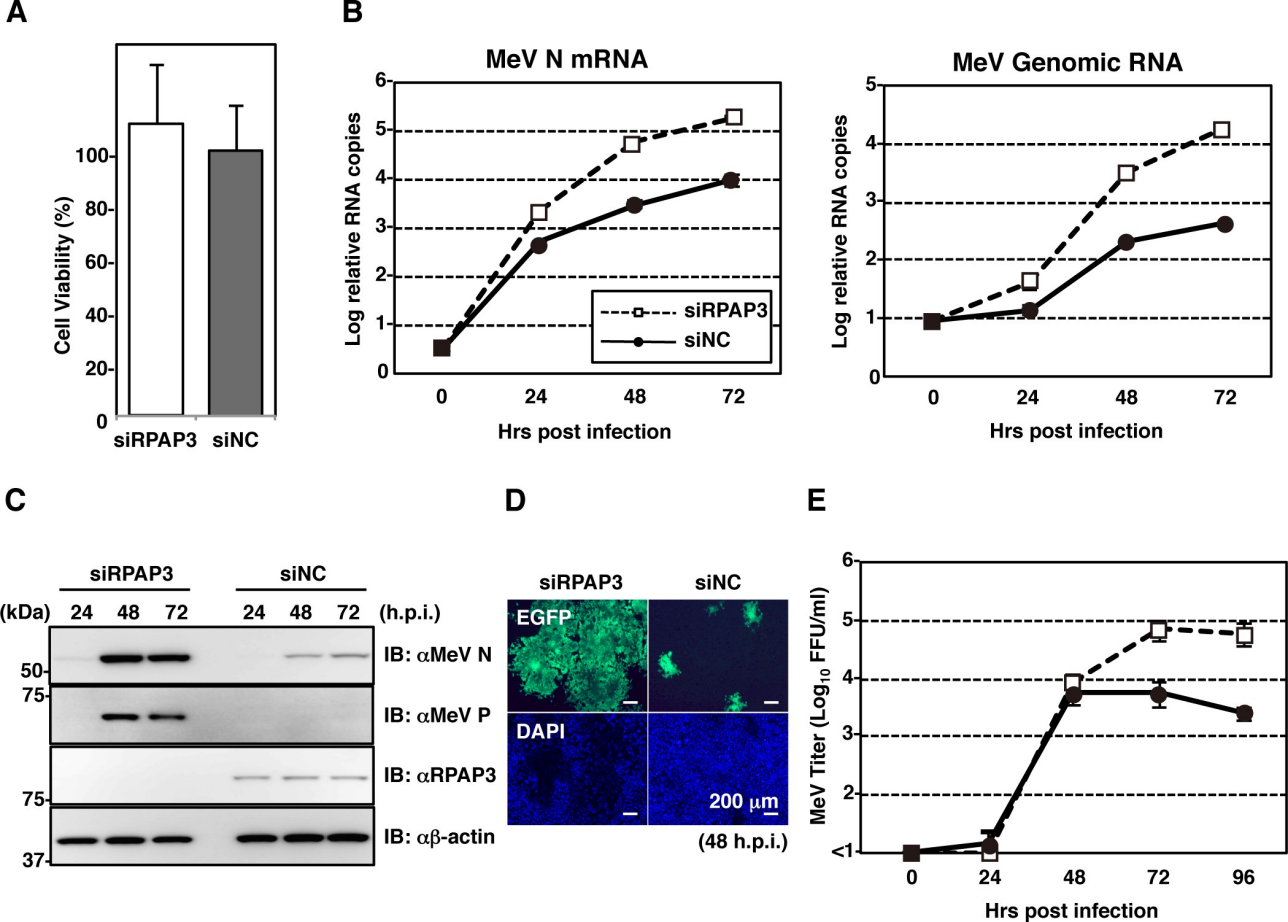
RPAP3 knockdown enhances MeV propagation. (**A**) At 96 h post-transfection with siRPAP3, cell viability was determined and calculated as a percentage of the viability of cells transfected with siNC. (**B-E**) At 48 h post-transfection with either siRPAP3 or siNC, A549/hSLAM cells were infected with rMeV/eGFP at a MOI of 0.05. Total cellular RNA and cell lysates were collected at 0, 24, 48 and 72 hpi, and relative levels of viral RNAs and proteins were analyzed by qPCR (B) and immunoblotting (C), respectively. (D) At 48 hpi, the cells were observed under a fluorescence microscope. (E) Supernatants were collected at 0, 24, 48, 72 and 96 hpi, and the infectious titers were determined by plaque assay. Error bars indicate the standard deviations of triplicate wells.

**Fig 5:**
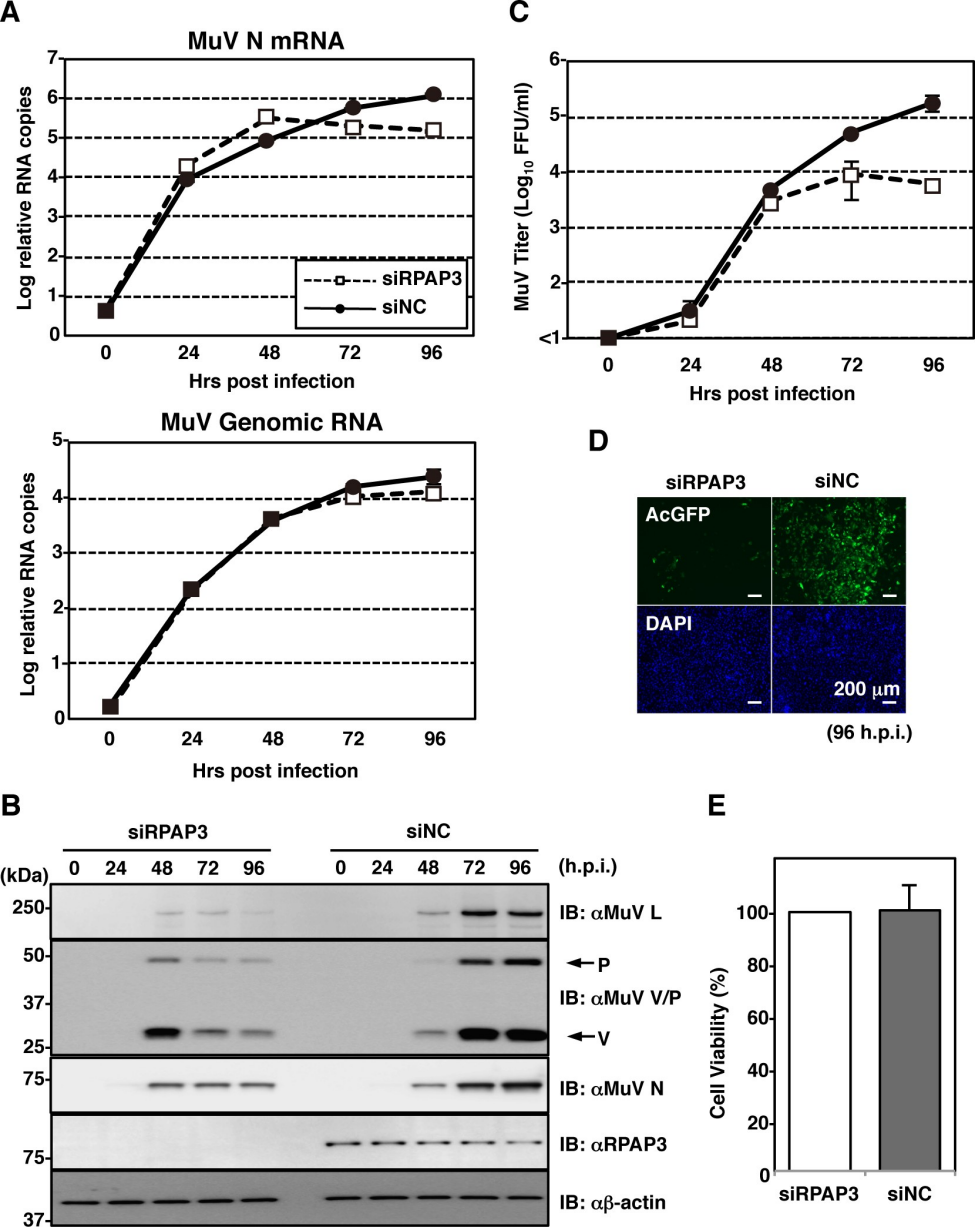
RPAP3 knockdown restricts MuV propagation. (**A-D**) At 48 h post-transfection with either siRPAP3 or siNC, A549 cells were infected with rMuV/AcGFP at a MOI of 0.05. Total cellular RNA and cell lysates were collected at 0, 24, 48, 72 and 96 hpi, and relative levels of viral RNAs and proteins were analyzed by qPCR (A) and immunoblotting (B), respectively. (C) Supernatants were collected at 0, 24, 48, 72 and 96 hpi, and infectious titers were determined by plaque assay. (D) At 96 hpi, cells were observed under a fluorescence microscope. (**E**) At 120 h post-transfection with siRPAP3, cell viability was determined and calculated as a percentage of the viability of cells transfected with siNC. Error bars indicate the standard deviations of triplicate wells.

The effect of RPAP3 knockdown was assessed in the setting of multiple-round viral infection. First, A549/hSLAM cells treated with siRPAP3 or siNC were infected with eGFP-expressing recombinant MeV at a MOI of 0.05. Cell viability was not affected by knockdown of RPAP3 (Fig 4A). As expected, levels of viral RNAs (both mRNA and genomic RNA) and proteins were upregulated in RPAP3-knockdown cells (Fig 4B–4D). The effect on viral titers in culture supernatants was delayed, but became evident after 48 hpi (Fig 4E). Next, A549 cells treated with siRPAP3 or siNC were infected with AcGFP-expressing rMuV at a MOI of 0.05. Until 48 hpi, levels of viral transcripts and proteins in RPAP3-knockdown cells were higher than those of control cells (Fig 5A and 5B). However, these levels decreased after 48 hpi. Genomic RNA levels were comparable until 48 hpi. Accordingly, titers of infectious MuV in RPAP3-knockdown cells were significantly suppressed after 48 hpi (Fig 5C and 5D). This unexpected result was unlikely due to cytotoxicity caused by RPAP3 knockdown (Fig 5E). Thus, for MuV, the effects of RPAP3 knockdown on multiple-round viral infection were consistent with the results of the one-step growth experiments until 48 hpi (Fig 3A), but an additional mechanism was necessary to explain the data after 48 hpi.

### RPAP3 knockdown induces strong host immune and inflammatory responses in MuV-infected cells

**Fig 6:**
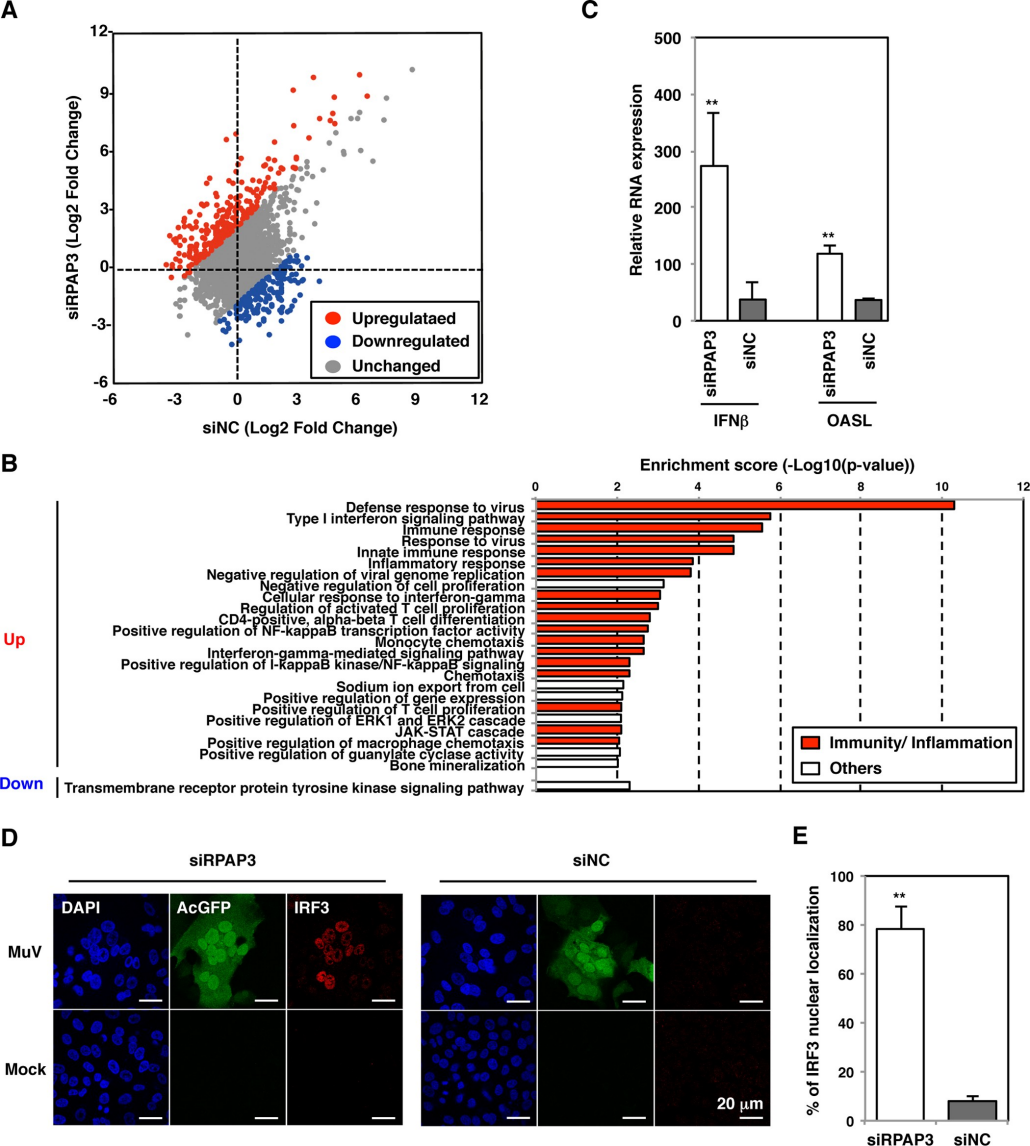
RPAP3 knockdown induces strong host immune and inflammatory responses in MuV-infected cells. (**A**) Scatter plot of differential gene expression upon MuV infection at 48 hpi in RPAP3-knockdown and control cells. Each dot represents the mean regulation value (log2-fold change of MuV-infected versus mock cells). x-axis: control cells, y-axis: RPAP3-knockdown cells. Genes with altered expression (>4-fold change) are colored in red and blue for upregulated and downregulated genes, respectively. (**B**) Gene ontology (GO) enrichment analysis of genes with altered expression (>4-fold change) between RPAP3-knockdown and control cells involved in the indicated biological processes. GO categories associated with immunity and inflammation are colored in red. (**C**) Levels of IFNβ and OASL mRNAs during MuV infection were measured relative to expression in mock-infected cells and normalized to levels of HPRT1 mRNA. (**D, E**) At 48 h post-transfection with either siRPAP3 or siNC, A549 cells were infected with rMuV/AcGFP at a MOI of 0.05. (D) At 48 hpi, cells were stained with mouse anti-IRF3 antibody and AF594-conjugated anti-mouse IgG. The cell nuclei were stained with DAPI (blue). (E) The percentages of cells with nuclear localization of IRF3 relative to the total number of infected cells were determined at 48 hpi using a fluorescence microscope. Error bars indicate the standard deviations of triplicate wells. The significance of differences between means was determined using the Student’s *t*-test. **p<0.01.

To understand the molecular mechanism of MuV growth suppression in RPAP3-knockdown cells after 48 hpi, viral and host gene expression were analyzed using RNA-Seq. A higher abundance of viral RNAs was observed in RPAP3-knockdown cells compared with control cells at 48 hpi (2.2% and 0.73% of total reads mapped to the MuV genome in RPAP3-knockdown and control cells, respectively). Expression of a large number of host genes was altered by MuV infection in both RPAP3-knockdown and control cells (S1 and S2 Tables). Scatter plots showed global differences in the transcriptomes of RPAP3-knockdown and control cells (Fig 6A). In total, 270 genes were upregulated in RPAP3-knockdown cells and 175 genes were upregulated in control cells (criteria: log_2_ fold change ≥2; S3 Table). We used gene ontology (GO) analysis to predict potential signaling pathways and cellular processes stimulated or suppressed by MuV infection in RPAP3-knockdown cells. Expression of large numbers of genes in GO categories associated with immunity and inflammation, such as defense against viruses, type I interferon signaling pathway, and inflammatory response, were stimulated by MuV infection (Fig 6B). Upregulation of innate immunity-related genes, such as interferon beta (IFNβ) and oligoadenylate synthetase-like (OASL), were confirmed by qRT-PCR (Fig 6C). To further confirm the stimulation of innate immunity in RPAP3-knockdown cells, but not in control cells, nuclear localization of IFN-regulatory factor 3 (IRF3) was demonstrated in these cells (Fig 6D and 6E). Expression of some innate immunity genes was modulated by RPAP3 knockdown even in uninfected control cells, but these effects were negligible when compared with MuV-infected cells (S5 Fig and S4 Table). These findings demonstrated that host immune and inflammatory responses were strongly stimulated during MuV infection in RPAP3-knockdown cells, but only minimally in control cells.

**Fig 7:**
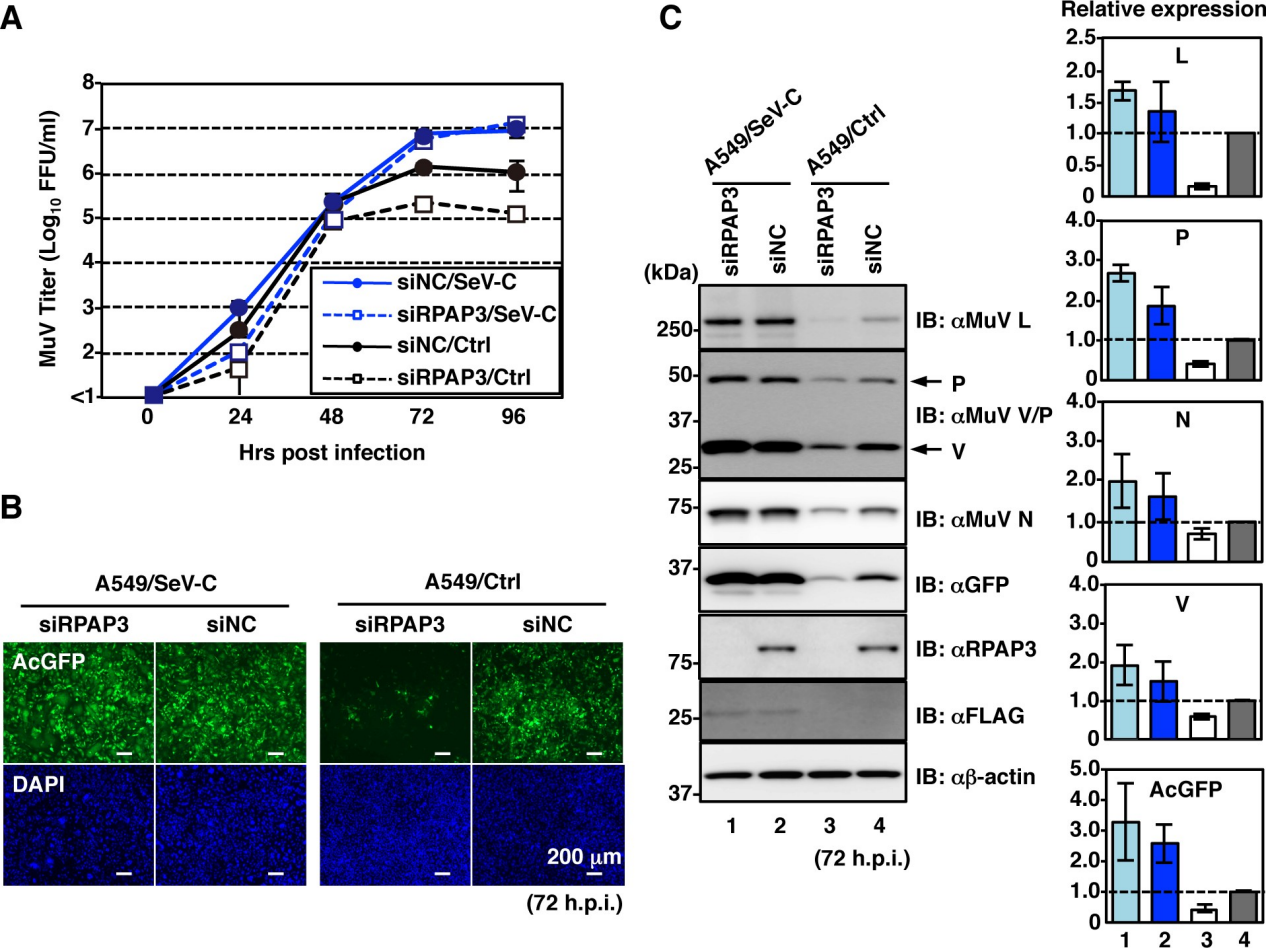
Host antiviral responses restrict MuV propagation in RPAP3-knockdown cells. At 48 h post-transfection with either siRPAP3 or siNC, A549/SeV-C and A549/Ctrl cells were infected with rMuV/AcGFP at a MOI of 0.05. (**A**) Supernatants were collected at 0, 24, 48, 72 and 96 hpi, and infectious titers were determined by plaque assay. (**B**) At 72 hpi, cells were observed under a fluorescence microscope. (**C**) At 72 hpi, cell lysates were immunoblotted with the indicated antibodies. The relative band intensities of each viral protein were normalized to β-actin level, and the relative expression of each protein based on levels in siNC-treated A549/Ctrl cells is shown. Error bars indicate the standard deviations of triplicate wells.

We further confirmed that the stimulated immune responses suppressed MuV growth in RPAP3-knockdown cells. Sendai virus C protein (SeV-C) has the ability to counteract host antiviral responses [27,28]. We assessed whether SeV-C could complement the growth defect of MuV in RPAP3-knockdown cells. To this end, we generated A549 cells constitutively expressing SeV-C (A549/SeV-C cells) as well as control A549 (A549/Ctrl) cells. In A549/Ctrl cells, RPAP3 knockdown significantly suppressed MuV growth after 48 hpi (Fig 7A–7C). By contrast, MuV continued to grow well in A549/SeV-C cells after 48 hpi, even if RPAP3 was knocked down. As expected, RNA-Seq analysis confirmed that host immune responses were minimal in A549/SeV-C cells (S6 Fig and S5–S10 Tables).

### RPAP3 knockdown does not disturb V protein functions

**Fig 8:**
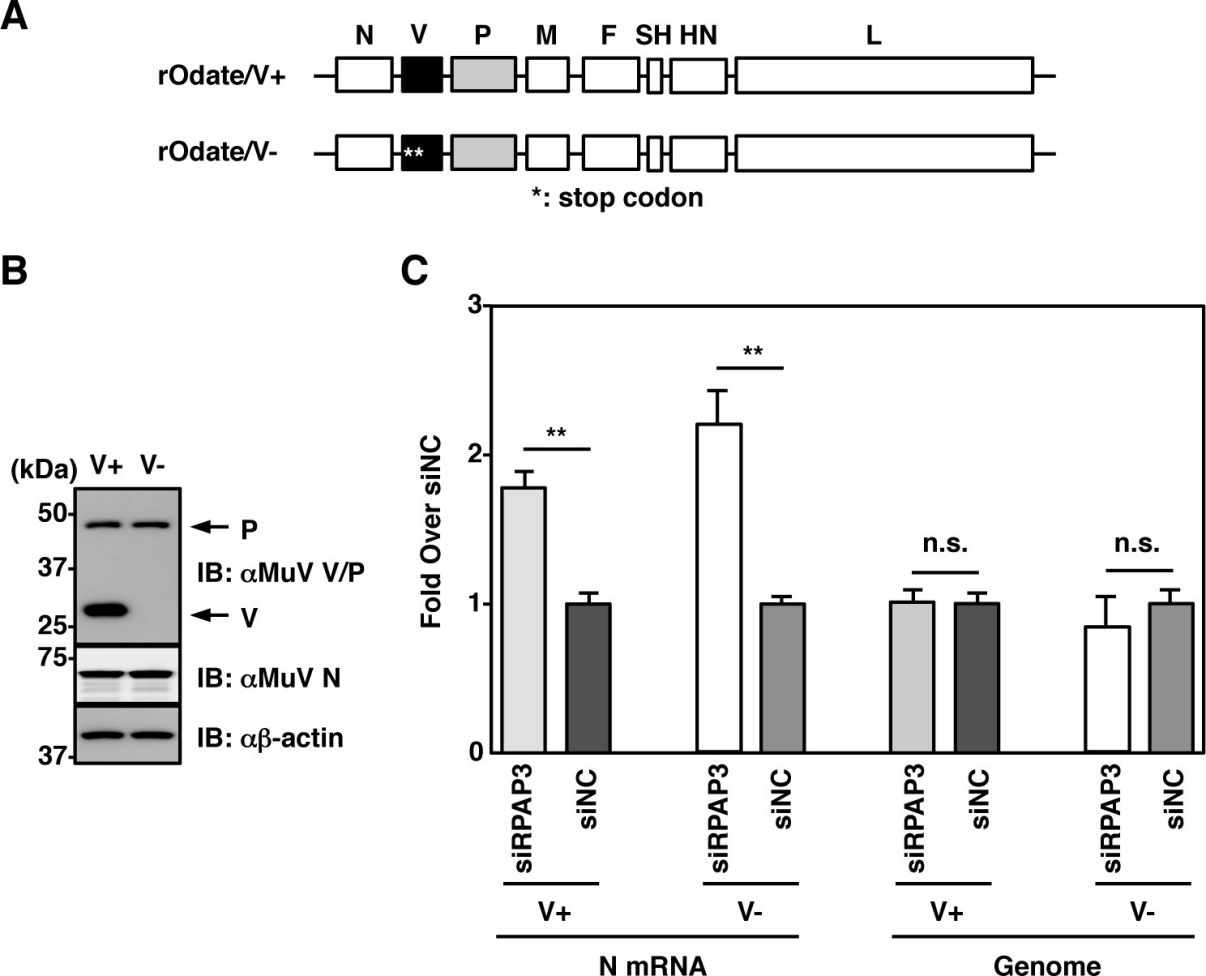
Enhanced viral transcription in RPAP3-knockdown cells is independent of V protein function. (**A**) Schematic of the rOdate/V^+^ and rOdate/V^-^ genes. (**B**) Vero cells were infected with rOdate/V^+^ or rOdate/V^-^ at an MOI of 3.0. Cell lysates were collected at 24 hpi and subjected to immunoblotting with the indicated antibodies. (**C**) At 48 h post-transfection with either siRPAP3 or siNC, A549 cells were infected with rOdate/V^+^ or rOdate/V^-^ at an MOI of 3.0 and incubated for 24 h. Relative levels of N mRNA and genomic RNA (based on levels in cells transfected with siNC) were determined by qPCR. Error bars indicate the standard deviations of triplicate wells. The significance of differences between means was determined using the Student’s *t*-test. **p<0.01. n.s. = not significant.

We found that RuvBL1 was also associated with the MuV V protein (Fig 1D). Because previous studies [29–31] demonstrated that the paramyxovirus V protein regulates viral RNA synthesis, an effect on the V protein may contribute to the enhancement of viral transcription in RPAP3-knockdown cells. To exclude the potential effects of the V protein, a recombinant MuV lacking V protein expression (rOdate/V^-^) and a control wild-type recombinant MuV (rOdate/V^+^) were generated (Fig 8A and 8B). Levels of viral mRNA in RPAP3-knockdown cells were higher than those in control cells and genomic RNA levels remained unchanged, regardless of V protein expression (Fig 8C). These data showed that regulation of viral RNA synthesis by the R2TP complex was independent of V protein function.

**Fig 9:**
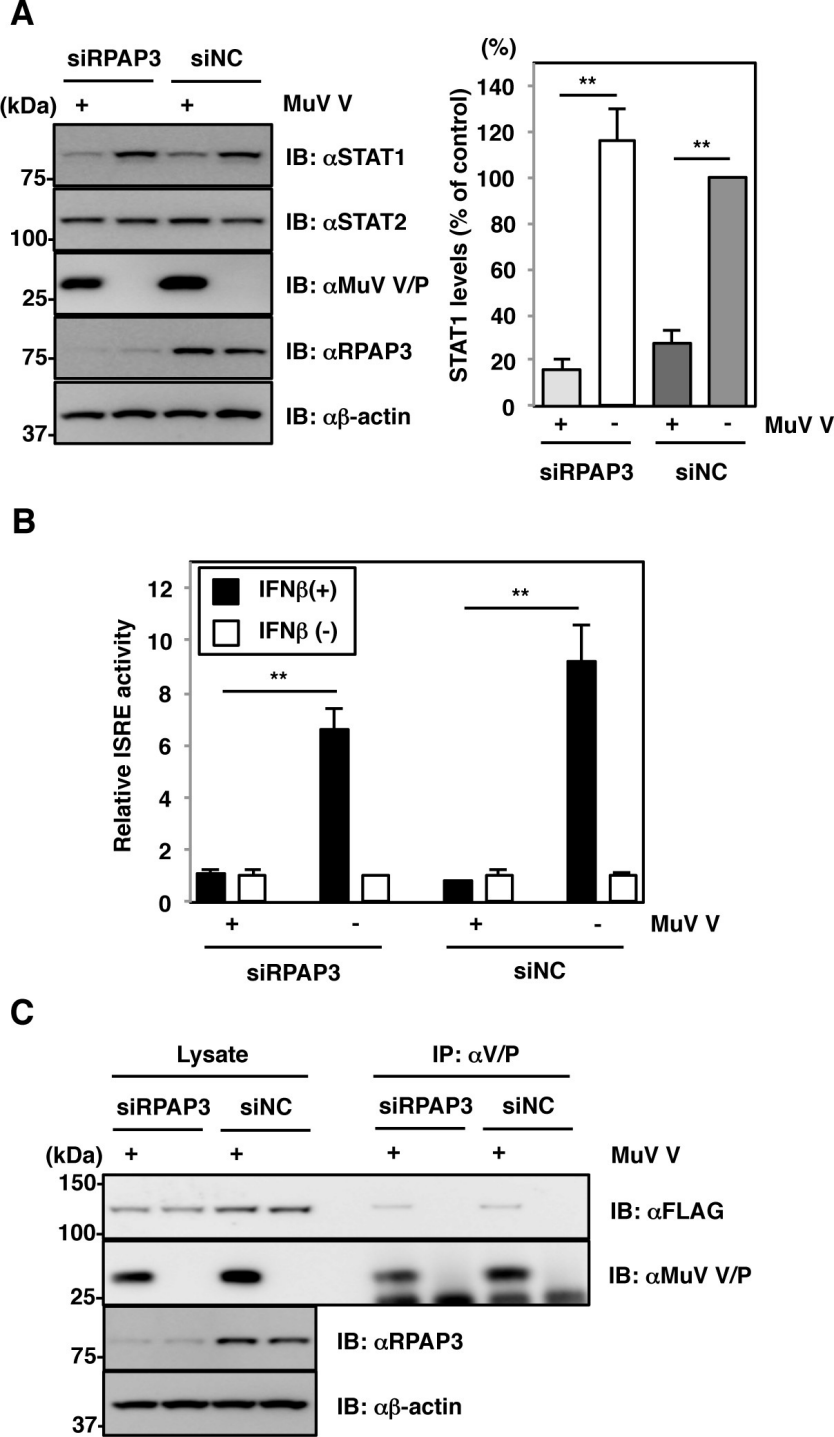
RPAP3 knockdown does not affect V protein function in anti-IFN responses. (**A**) 293T cells were co-transfected with pCAGGS-V or empty vector and siRPAP3 or siNC. At 72 h post-transfection, cell lysates were immunoblotted with the indicated antibodies. Relative STAT1 band intensities were normalized to β-actin level, and the relative expression of each protein based on levels in siNC-treated and empty vector-transfected cells are shown. (**B**) 293T cells were co-transfected with plasmids pISRE-luc13-p92, pRL-TK and pCAGGS-V or empty vector along with siRNA. At 72 h post-transfection, the cells were treated with IFNβ for 6 hr. Luciferase activities (based on levels in IFNβ-untreated cells) were quantified by normalizing to *Renilla* luciferase activities. (**C**) 293T cells were co-transfected with plasmids pEF-FLAG-MDA5 and pCAGGS-V or empty vector and siRNA. At 72 h post-transfection, cell lysates were immunoprecipitated with anti-MuV V/P antibody and immunoblotted with the indicated antibodies. Error bars indicate the standard deviations of triplicate wells. The significance of differences between means was determined using the Student’s *t*-test. **p<0.01.

The MuV V protein also contributes to counteracting innate immune responses. The V protein blocks IFN signaling by targeting STAT1 degradation [32] and also suppresses IFN production by interacting with MDA5 [33]. Therefore, the effect of RPAP3 knockdown on the anti-IFN function of the MuV V protein was analyzed. The V protein reduced expression levels of STAT1 (Fig 9A) and blocked type I IFN signaling (Fig 9B). This effect was evident even in RPAP3-knockdown cells (Fig 9A and 9B). Furthermore, the V protein retained its ability to interact with MDA5 in RPAP3-knockdown cells (Fig 9C). These findings suggest that the interaction of the R2TP complex with the L protein, but not the V protein, is critical for regulation of viral RNA synthesis and inhibition of host immune responses.

## Discussion

The paramyxovirus RNP is a large and multifunctional protein-RNA complex. The process of its formation and its many functional regulation mechanisms remain to be elucidated. Previous studies showed that Hsp90 assists in RNP formation through interaction with the paramyxovirus L protein [11,12]. In this study, we searched for additional host factors that interacted with the L protein and contributed to the formation or function of the RNP complex. We found that the R2TP complex is a novel host factor interacting with the paramyxovirus L protein. The R2TP complex is a Hsp90 co-chaperone and facilitates assembly of multi-proteins and protein-RNA complexes [23]. Thus, we initially expected that the R2TP complex might be a host factor promoting RNP formation or function. However, paramyxovirus RNA synthesis was instead accelerated when expression of the R2TP complex was silenced. This result was surprising, but our further analyses demonstrated that the R2TP complex has a regulatory or suppressive function on RNA synthesis of the paramyxovirus L protein.

Precise control of viral RNA synthesis is critically important for paramyxoviruses. The L protein exhibits all the major catalytic activities required for RNA synthesis, and the RNP complex is the minimum viral component required for viral RNA synthesis. Many paramyxoviruses encode nonstructural proteins, such as the V and C proteins. These proteins are nonessential for viral replication, but have a regulatory or suppressive role on viral RNA synthesis. By finely tuning viral RNA synthesis and preventing the synthesis of aberrant RNA species, these proteins indirectly contribute to inhibition of host anti-viral responses [34]. In many cases, excessive viral RNA synthesis due to mutations or deletions in nonstructural proteins or transcriptional regulatory regions results in induction of type I IFNs and proinflammatory cytokines. The evidence indicates that optimal control of RNA synthesis is a major strategy of paramyxovirus evasion of the host immune response. In addition, these nonstructural proteins directly function to circumvent host antiviral responses by interfering with the induction of antiviral responses or IFN signaling pathways. Our data demonstrated that suppression of RPAP3 modulated MuV RNA synthesis, causing enhanced transcription and in turn strongly stimulating host innate immune responses. Although RuvBL1 interacted with both MuV L and V proteins, the effect of RPAP3-knockdown was evident in the V protein-deficient MuV. Moreover, knockdown of RPAP3 showed little effect on V protein function. Thus, the R2TP complex is a host factor interacting with the L protein, but not the V protein, to control MuV propagation.

MeV RNA synthesis was also greatly enhanced by RPAP3 suppression. Interestingly, patterns of MuV RNA synthesis in RPAP3-knockdown cells were very similar to those of C protein-knockout MeV. In the absence of the C protein, MeV transcription is also enhanced [35]. Growth of the C protein-knockout MeV is strongly inhibited in cells due to stimulation of host innate immune responses [36,37]. By contrast, MuV does not encode a C protein or a homologue. Our data thus demonstrated that in A549 cells, the host R2TP complex functions as a host factor, precisely regulating MuV RNA synthesis, just as the C protein does for MeV RNA synthesis.

We demonstrated that suppression of RPAP3 increased the amounts of viral RNAs in MuV- and MeV-infected cells. Increased polymerase activity is one possible explanation for this observation. However, there remain other possible explanations. We confirmed that RPAP3 knockdown did not affect the formation of inclusion bodies and the cellular localization of viral RNPs. These data suggested that the R2TP complex does not contribute to RNP transport. In addition, the increased levels of viral RNA and protein were shown by one-step growth experiments and a minigenome assay, further supporting a role of the R2TP complex in the process of RNA synthesis. However, it remains possible that the increased viral RNA levels are due to increased viral RNA stability, not higher viral polymerase activity. Further investigation is required to clarify the molecular mechanisms through which the R2TP complex regulates paramyxovirus infection.

The paramyxovirus L protein showed a unique interaction with the R2TP complex. RPAP3 acts as a scaffold that assembles the Hsp90/R2TP complex [38]. RPAP3 interacts with Hps90, PIH1D1 and RuvBL2 via its N-terminal TPR domain, its central domain and its C-terminal domain, respectively. Many client proteins are recruited to the R2TP complex through interactions with the N-terminal domain of PIH1D1 [26]. By contrast, the paramyxovirus L protein was associated with the R2TP complex through interaction with both RPAP3 and the C-terminal region of PIH1D1, independent of Hsp90. Both Hsp90 and the R2TP complex interacted with the L protein, but our data using the RPAP3 N321E mutant showed that the interaction between Hsp90 and R2TP complex was also necessary to regulate paramyxovirus RNA synthesis. Maturation of the RdRps of other negative-strand RNA viruses, such as respiratory syncytial virus [39], vesicular stomatitis virus [10], Ebola virus [40] and influenza virus [41,42], also depends on Hsp90 activity. RuvBL1 and RuvBL2 are ATPases associated with various cellar activities [25]. A previous study showed that RuvBL2 binds to the influenza virus N protein and inhibits viral replication by interfering with N protein oligomerization [43]. In the R2TP complex, RuvBL1/RuvBL2 and Hsp90 promote assembly and maturation of protein complexes when client proteins are recruited [16,22,44]. The roles of RPAP3 and PIH1D1 appear to be dedicated to the R2TP complex, while RuvBL1 and RuvBL2 exhibit a variety of functions independently of the R2TP complex [25]. The influenza virus study focused on the role of RuvBL2, and the role of the R2TP complex in influenza virus replication is unclear [43]. Nevertheless, the inhibitory mechanism of RuvBL2 in influenza virus infection was totally different from that proposed for paramyxoviruses in this study. Further investigation will be required to define the roles of the Hsp90/R2TP system in RNA synthesis of diverse RNA viruses.

In conclusion, we have demonstrated that the R2TP complex is a novel host factor that precisely regulates MuV RNA synthesis by interacting with the L protein. This regulation was critical for MuV evasion of host innate immune responses to ensure its replication. The R2TP complex also functioned as a suppressor of MeV RNA synthesis, but its functions were inhibitory and not beneficial to MeV. Further studies are necessary to elucidate the mechanisms underlying the difference between MuV and MeV. However, this initial identification of a host factor regulating paramyxovirus RNA synthesis should shed light on the regulatory mechanisms controlling paramyxovirus RNA synthesis.

## Materials and methods

### Cell culture, viruses and transfection

Vero (African green monkey kidney) cells were provided by the U.S. Food and Drug Administration and were maintained in Dulbecco’s modified Eagle’s medium (DMEM) (Nacalai Tesque, Kyoto, Japan) supplemented with 100 U/mL penicillin and 100 mg/mL streptomycin (P/S) and 10% fetal bovine serum (FBS). A549 (human lung epithelial) (ATCC CCL-185) and 293T (human kidney) (ATCC CRL-3216) cells were purchased from the American Type Culture Collection (Manassas, VA) and cultured in DMEM containing P/S and 10% FBS. Baby hamster kidney (BHK) cells constitutively expressing T7 RNA polymerase (BHK/T7-9) were kindly provided by Naoto Ito [45] and maintained in Glasgow minimal essential medium (Nacalai Tesque) supplemented with 5% FBS and 5% (v/v) tryptose phosphate broth. Vero/hSLAM [46] and A549/hSLAM [47] cells were generated as described previously and maintained in DMEM containing 10% FBS and 0.2 mg of G418. A549/hSLAM cells were transduced with a retroviral vector encoding the SeV C protein (A549/SeV-C cells) and cultured in DMEM containing 10% FBS and 5 μg/mL puromycin. Cell viability was measured using a CellTiter-Glo Luminescent Cell Viability Assay Kit (Promega, Madison, WI).

A recombinant MuV expressing AcGFP (rMuV/AcGFP) [12] and a recombinant MeV expressing eGFP (rMeV/eGFP) [48] were used in this study. Infectious titers of MuV and MeV were determined by focus forming assay using Vero and Vero/SLAM cells, respectively.

Cells were transfected using TransIT-LT1 Reagent (Mirus/TaKaRa Bio Inc, Shiga, Japan) for plasmid DNAs, Lipofectamine RNAiMax (Invitrogen/Thermo Fisher Scientific Inc., Waltham, MA) for siRNAs or DharmaFECT Duo Transfection Reagent (Dharmacon/Horizon Discovery, Lafayette, CO) for cotransfection of plasmid DNAs and siRNAs according to the manufacturer’s instructions.

### Plasmids

Plasmids pCR-N, -P and–L, pCAGGS-V and pCAGGS-HA-MuV-L_Full_ (for expression of N-terminally HA-tagged full-length MuV L protein) were generated as previously described [12,49]. The cDNAs encoding human RuvBL1, RuvBL2, RPAP3, PIH1D1 and Hsp90 were amplified from 293T cells by RT-PCR and cloned into pcDNA3.1-FLAG (for expression of N-terminally FLAG-tagged proteins), resulting in the vectors pcDNA-FLAG-RuvBL1, -RuvBL2, -RPAP3, -PIH1D1 and -Hsp90, respectively. The R2TP cDNAs were also cloned into pGEX-6P-1 (for expression of N-terminally GST-fused proteins in *E*. *coli*) (GE Healthcare, Chicago, IL), resulting in the vectors pGEX-GST-RuvBL1, -RuvBL2, -RPAP3, -PIH1D1, respectively. A series of deletion mutants of HA-tagged MuV L protein and FLAG-tagged PIH1D1 were generated by PCR-based mutagenesis. A fragment of the MuV L protein was amplified from pCAGGS-HA-MuV-L_Full_ by PCR and cloned into pCAGMCS2-FOS [24], resulting in the vector pCAG-MuV-L_1-900_-FOS. A fragment of the MeV L protein was amplified from pCAGGS-HA-MeV-L_Full_ [12] by PCR and cloned into pCAGGS-HA, resulting in the vector pCAGGS-HA-MeV-L_1-900_. Mutants of FLAG-tagged RPAP3 with either or both of Asn^172^ and Asn^321^ replaced by Glu and silent mutants of FLAG-RPAP3 (FLAG-RPAP3_siR) were also generated by PCR-based mutagenesis.

Based on the MeV minigenome expression plasmid, p18MGFLuc01 [50], a plasmid encoding a MeV minigenome, a luciferase reporter flanked by the leader and trailer sequences of MeV, under the control of EF-1α promoter was constructed (pEF-HHR-MGF). The construct contained hammerhead and hepatitis delta ribozyme sequences for correct processing of the 5’ and 3’ termini of the minigenomic RNA. The cDNAs of the MeV IC-B N, P and L proteins were cloned into the pCA7 vector [47], resulting in vectors pCA7-N,—PΔC and–L, respectively.

A full-length cDNA clone of MuV Odate strain (pMuV-Odate) was constructed as previously described [51]. This plasmid was modified to express the V and P proteins from separate transcriptional units by changing the editing site of the V/P gene (GGGGGCC to AGGAGGGCC) for the P protein expression only and by introducing an additional transcriptional unit encoding the V gene between the N and V/P genes (pMuV-Odate/V^+^). The plasmid pMuV-Odate/V^+^ was further modified to abrogate expression of the V protein by introducing two stop codons in the V gene (pMuV-Odate/V^-^).

Sequences of all plasmids were confirmed using an ABI Prism 3130*xl* genetic analyzer (Applied Biosystems/Thermo Fisher Scientific Inc.).

### Virus rescue

BHK/T7-9 cells were co-transfected with the plasmids pMuV-Odate/V^+^ or pMuV-Odate/V^-^ (5 μg) and pCR-N (100 ng), pCR-P (100 ng) and pCR-L (500 ng) using TransIT-LT1 Reagent. At 48 h post-transfection, the transfected BHK/T7-9 cells were mixed 1:1 with Vero cells. At the height of the cytopathic effect, the supernatants were collected, and the full-length genome sequences of the rescued viruses were determined.

### Antibodies

Anti-MuV V/P (T61) and L (L17) and anti-MeV P rabbit polyclonal antibodies (PAbs) were described previously [37,49,52]. Anti-MuV N rabbit PAb was provided from DENKA SEIKEN (Niigata, Japan). Anti-FLAG (M2) and anti-β-actin (AC-15) mouse monoclonal antibodies (MAbs) were purchased from Sigma (St. Louis, MO). Anti-STAT1 (sc-346) and anti-STAT2 (sc-476) rabbit PAbs were purchased from Santa Cruz Biotechnology (Dallas, TX). Anti-HA (HA11), anti-PIH1D1 (ab57512), anti-Hsp90 (Hyb-K41009) and anti-IRF3 (SL-12.1) mouse MAbs and anti-MeV N (NB100-1856), anti-RuvBL1 (#12300) and anti-RPAP3 (A304-854A) rabbit PAbs were purchased from BioLegend Inc. (San Diego, CA), Abcam (Cambridge, UK), StressMarq Bioscience Inc. (Victoria, Canada), BD Pharmingen/BD Bioscience (San Jose, CA), Novus Biologicals (Littleton, CO), Cell Signaling Technology (Danvers, MA) and Bethyl Laboratories Inc. (Montgomery, TX), respectively.

### Cell extracts, immunoblotting and immunoprecipitation

For preparation of cell extracts, cells were washed twice with cold phosphate-buffered saline (PBS) and then lysed in cell lysis buffer (20 mM Tris-HCl, pH 7.5, containing 135 mM NaCl, 1% Triton-X 100, and protease inhibitor cocktail [Complete Mini; Roche, Mannheim, Germany]). For immunoblotting, the cell lysate was boiled in SDS sample buffer and subjected to SDS-PAGE. The proteins were transferred to polyvinylidene difluoride membranes (Millipore, Bedford, MA) and incubated with the appropriate antibodies. Each protein was visualized using SuperSignal West Femto Maximum Sensitivity Substrate (Thermo Fisher Scientific Inc.) and detected using of an LAS-3000 image analyzer system (Fuji Film, Tokyo, Japan). For immunoprecipitation, the cell lysate was pre-cleaned with protein G-sepharose (GE Healthcare). Antibody-protein complexes were purified with protein G beads and washed with cell lysis buffer three times. After boiling in SDS sample buffer, the proteins were separated by SDS-PAGE and processed for immunoblotting. Preparation and immunoprecipitation of each cell extract were performed at least three times, and representative data are shown.

### Pull-down assay

GST-RuvBL1, -RuvBL2, -PIH1D1 and -RPAP3 were expressed in *E*. *coli* Rosetta-gami 2 (DE3) cells (Novagen/Merck Millipore, Darmstadt, Germany) transformed with pGEX-GST-RuvBL1, -RuvBL2, -PIH1D1 and -RPAP3, respectively. For preparation of GST-fused R2TP components, bacteria were lysed in bacteria lysis buffer (50 mM Tris-HCl, pH 7.5, containing 150 mM NaCl, 1 mM EDTA, 1% Triton-X 100, and protease inhibitor cocktail) and centrifuged at 15,000 x *g* for 20 min. The supernatants were mixed with glutathione-sepharose 4B beads (GE Healthcare) for 2 h at 4°C and washed with bacteria lysis buffer three times. HA-MuV-L_1-900_ was expressed in a wheat germ cell-free expression system using the Premiun PLUS Expression Kit (CellFree Science, Ehime, Japan). A solution containing HA-MuV-L_1-900_ was mixed with each purified GST-fused protein for 2 h at 4°C, and then the beads were washed with bacteria lysis buffer three times and suspended in SDS-PAGE sample buffer. The experiment was performed at least three times, and representative data are shown.

### Quantitative RT-PCR (qRT-PCR)

Total RNA was extracted using the RNeasy Mini Kit (Qiagen, Hilden, Germany) and first-strand cDNA was synthesized using PrimeScript RTase (Takara Bio Inc.). The reverse transcription reaction was performed using the following primers: 5’-ACCAAGGGGAAAATGGAGATG-3’ (complementary to nucleotides 1 to 21 of the MuV genome) for MuV genomic RNA, 5’-ACCAAACAAAGTTGGGTAAGG-3’ (complementary to nucleotides 1 to 21 of the MeV genome) for MeV genomic RNA and an oligo(dT) primer for viral and cellular mRNAs. The amount of each cDNA was measured using the Universal ProbeLibrary and the LightCycler 480 system (Roche) according to the manufacturer’s instructions. The levels of each RNA were normalized to that of hypoxanthine phosphoribosyltransferase 1 (HPRT1) mRNA. The qPCR for the MuV N and HPRT1 genes was performed as described previously [12]. The qPCR for the MuV V/P gene was performed using a Universal ProbeLibrary Probe #134 (Roche) and the following primers: 5’-GGCCCATCATAGTCTCATCC-3’ and 5’-GAAAAGGGGCTCAGGAATCT-3’, specific for a fragment consisting of nucleotides 2208 to 2272 of the MuV genome. The qPCR for the MeV N gene was performed using a Universal ProbeLibrary Probe #85 (Roche) and the following primers: 5’-TCACATGATGATCCAAGCAGT-3’ and 5’-TTTCCTTGTTCTCGAACCATC-3’, specific for a fragment consisting of nucleotides 394 to 454 in the MeV genome. The qPCR for the IFNβ gene was performed using a Universal ProbeLibrary Probe #20 (Roche) and the following primers: 5’-CTTTGCTATTTTCAGACAAGATTCA-3’ and 5’-GCCAGGAGGTTCTCAACAAT-3’, specific for a fragment consisting of nucleotides 355 to 423 of the IFNβ gene. The qPCR for the OASL gene was performed using a Universal ProbeLibrary Probe #68 (Roche) and the following primers: 5’-ATGTTGGACGAAGGCTTCAC-3’ and 5’-TTGGTCCAGTAGATACAGATGACTTC-3’, specific for a fragment consisting of nucleotides 763 to 836 of the OASL gene. The Experiments were performed at least three times, and representative data are shown.

### Minigenome assay

293T cells in 24-well plates were transfected with 200 ng of pCA7-N, 50 ng of pCA7-PΔC, 200 ng of pCA7-L, 100 ng of pEF-HHR-MGF, 25 ng of pRL-TK (Promega) and 500 ng of pcDNA-FLAG-RPAP3_siR or empty vector along with 10 pmol of siRNA using DharmaFECT Duo Transfection Reagent. After 72 h, enzymatic activities of firefly and *Renilla* luciferases were measured using a Dual-Luciferase Reporter Assay System (Promega) and a GloMax 20/20 Luminometer (Promega). Relative luciferase activity was defined as the ratio of firefly to *Renilla* luciferase activity. The experiment was performed at least three times, and representative data are shown.

### Luciferase reporter assay

293T cells in 24-well plates were transfected with 100 ng of pISRE-luc13-p92, 25 ng of pRL-TK and 300 ng of pCAGGS-V or empty vector along with 10 pmol of siRNA using DharmaFECT Duo Transfection Reagent. The cells were treated with 500 U/mL of recombinant human IFNβ (Toray Industries Inc,. Tokyo, Japan) starting at 72 h post-transfection. After 6 h of incubation, the cells were lysed, and enzymatic activities of firefly and *Renilla* luciferases were measured using a Dual-Luciferase Reporter Assay System and a GloMax 20/20 Luminometer. Relative luciferase activity was defined as the ratio of firefly to *Renilla* luciferase activity. The experiment was performed at least three times, and representative data are shown.

### Gene silencing

The siRNAs siRuvBL1 (5’-AGAGCAUGUCGAAGAGAUC-3’), siPIH1D1 (5’-CCCGCUGCAGAUCAACUCUCA-3’) and siRPAP3 (5’-UUGAAGGAUAGUUCUGUCGAA-3’) were used to silence endogenous RuvBL1, PIH1D1 and RPAP3 expression, respectively [53,54]. A non-targeting siRNA pool (siNC) (Dharmacon/Horizon Discovery) was used as a negative control.

### Purification of FOS-tagged proteins and mass spectrometry

293T cells were transfected with pCAGGS-L_1-900_-FOS or empty vector. Cells were harvested at 24 h post-transfection, washed twice with ice-cold PBS, suspended in cell lysis buffer, and centrifuged at 14,000 x *g* for 20 min at 4°C. The supernatant was pulled down using 50 μL of STrEP-Tactin Sepharose (IBA, Gottingen, Germany) equilibrated with cell lysis buffer for 2 h at 4°C. The affinity beads were washed three times with cell lysis buffer and suspended in 2x SDS-PAGE sample buffer. The proteins were separated by SDS-PAGE, followed by oriole staining using Oriole Fluorescent Gel Stain (BioRad, Hercules, CA). The gel was divided into 21 pieces per lane, and each piece was trypsinized and subjected to liquid chromatography-tandem mass spectrometry (LC-MS/MS) analysis to identify coimmunoprecipitated proteins. All proteins in the gel were identified comprehensively, and proteins detected in cells transfected with pCAGGS-L_1-900_-FOS but not in cells transfected with empty vector were regarded as candidate binding partners of the polymerase region of MuV L protein.

### Immunofluorescence microscopy

Cells were fixed in PBS containing 4% paraformaldehyde for 15 min at room temperature. The cells were permeabilized with PBS containing 0.2% Triton X-100 for 10 min, blocked with PBS containing 2% bovine serum albumin for 30 min at room temperature, and incubated with the indicated antibodies. Nuclei were stained with 4’, 6-diamidino-2-phenylindole (DAPI). Samples were examined under a FV1000D confocal laser-scanning microscope (Olympus, Tokyo, Japan). The experiments were performed at least three times, and representative data are shown.

### RNA-Seq

Total RNA was isolated in duplicate using the RNeasy Mini Kit with DNase I treatment and depleted of rRNA using the NEBNext rRNA Depletion Kit (New England Biolabs (NEB), Ipswich, MA). RNA-Seq libraries were generated using the NEBNext Ultra II Directional RNA Library Prep Kit for Illumina (NEB) and pooled in equimolar amounts using the QuantiFluor dsDNA system (Promega). The quality and quantity of each pool were assessed using the 2100 analyzer (Agilent Technologies, Santa Clara, CA). Sequencing was performed on one lane of an Illumina HiSeq X Ten instrument (Illumina, SanDiego, CA), generating 2 x 150 bp paired-end reads. After quality control, the sequences were mapped to human genome sequences (Genome Reference Consortium Human Reference 38 [GRCh38/hg38] [GCA_000001405.33]) using the RNA-Seq analysis tool in the CLC Genomics Workbench 9.0.1 (Qiagen). RNA-Seq analyses using the human genome were performed with GRCh38.91 annotation data. Differentially expressed genes between infected and uninfected cells were identified at combined cut-offs of as a false discovery rate <0.01 and |log_2_ fold change|>1. Statistics related to overrepresentation of functional categories were calculated using DAVID [55]. A *p*-value <0.01 was considered statistically significant. The sequence data described in this study have been submitted to the DDBJ Sequence Read Archive (DRA) under accession number DRA007268.

### Statistical analysis

Differences between groups were evaluated using unpaired Student’s *t* tests. Error bars indicate the standard deviations of triplicate measurements. Statistical significance was assumed at **p<0.01. n.s. = not significant.

## Supporting information

S1 Fig Hsp90 interacts with the N-terminal region of MuV L protein. A series of truncated mutants of HA-tagged MuV L protein were expressed in 293T cells, immunoprecipitated with anti-HA antibody, and immunoblotted with anti-HA and anti-Hsp90 antibodies. Asterisks indicate the bands corresponding to each mutant.
(TIF)

S2 Fig MuV L protein interacts with the C-terminal region of PIH1D1. FLAG-PIH1D1 and its truncated mutants were co-expressed with HA-MuV-L_1-900_ in 293T cells, immunoprecipitated with anti-FLAG antibody and immunoblotted with the indicated antibodies.
(TIF)

S3 Fig RPAP3 knockdown does not affect the localization of viral nucleocapsid. At 48 h post-transfection with either siRPAP3 or siNC, A549 and A549/hSLAM cells were infected with rMuV/AcGFP and rMeV/EGFP, respectively, at an MOI of 0.05. At 48 hpi, cells were stained with rabbit anti-MuV N or mouse anti-MeV N antibody and AF594-conjugated anti-rabbit or -mouse IgG. The cell nuclei were stained with DAPI (blue).
(TIF)

S4 Fig The TPR2 region of RPAP3 is important for interaction with Hsp90. FLAG-RPAP3 and its mutants were co-expressed with HA-MuV-L_1-900_ in 293T cells, immunoprecipitated with anti-FLAG antibody and immunoblotted with the indicated antibodies.
(TIF)

S5 Fig siRNA treatment does not induce IFNβ gene expression. The levels of IFNβ mRNA in A549 cells at 48 h post-transfection with either siRPAP3 or siNC were measured relative to their expression in untransfected cells and normalized to levels of HPRT1 mRNA. Error bars indicate the standard deviations of triplicate wells. The significance of differences between means was determined using the Student’s *t*-test. n.s. = not significant.
(TIF)

S6 Fig Transcriptome analysis of A549/SeV-C and A549/Ctrl cells following MuV infection. At 48 h post-transfection with either siRPAP3 or siNC, A549/SeV-C and A549/Ctrl cells were infected with rMuV/AcGFP at an MOI of 0.05. (**A**) Scatter plots of differential gene expression following MuV infection at 48 hpi in RPAP3-knockdown and control cells. Each dot represents the mean expression value (log2-fold change of MuV-infected versus mock cells). x-axis: control cells, y-axis: RPAP3-knockdown cells. Genes with altered expression (>4-fold change) are colored in red and blue for upregulation and downregulation, respectively. (**B**) GO enrichment analysis of genes with altered expression (>4-fold change) between RPAP3-knockdown and control cells involved in the indicated biological processes. GO categories associated with immunity and inflammation are colored in red.
(TIF)

S1 Table List of differentially expressed genes in MuV-infected and uninfected RPAP3-knockdown A549 cells. (PDF)

S2 Table List of differentially expressed genes in MuV-infected and uninfected control A549 cells. (PDF)

S3 Table List of genes upregulated or downregulated following MuV infection in RPAP3-knockdown A549 cells. (PDF)

S4 Table List of differentially expressed genes in mock-infected RPAP3-knockdown and control A549 cells. (PDF)

S5 Table List of differentially expressed genes in MuV-infected and uninfected RPAP3-knockdown A549/SeV-C cells. (PDF)

S6 Table List of differentially expressed genes in MuV-infected and uninfected control A549/SeV-C cells. (PDF)

S7 Table List of differentially expressed genes in MuV-infected and uninfected RPAP3-knockdown A549/Ctrl cells. (PDF)

S8 Table List of differentially expressed genes in MuV-infected and uninfected control A549/Ctrl cells. (PDF)

S9 Table List of genes upregulated or downregulated following MuV infection in RPAP3-knockdown A549/SeV-C cells. (PDF)

S10 Table List of genes upregulated or downregulated following MuV infection in RPAP3-knockdown A549/Ctrl cells. (PDF)
