# Supplementary figures and images for: The R2TP complex regulates paramyxovirus RNA synthesis
Source: PLoS Pathog. 2019 May 23;15(5):e1007749. doi: 10.1371/journal.ppat.1007749 (PMC6532945; doi:10.1371/journal.ppat.1007749)

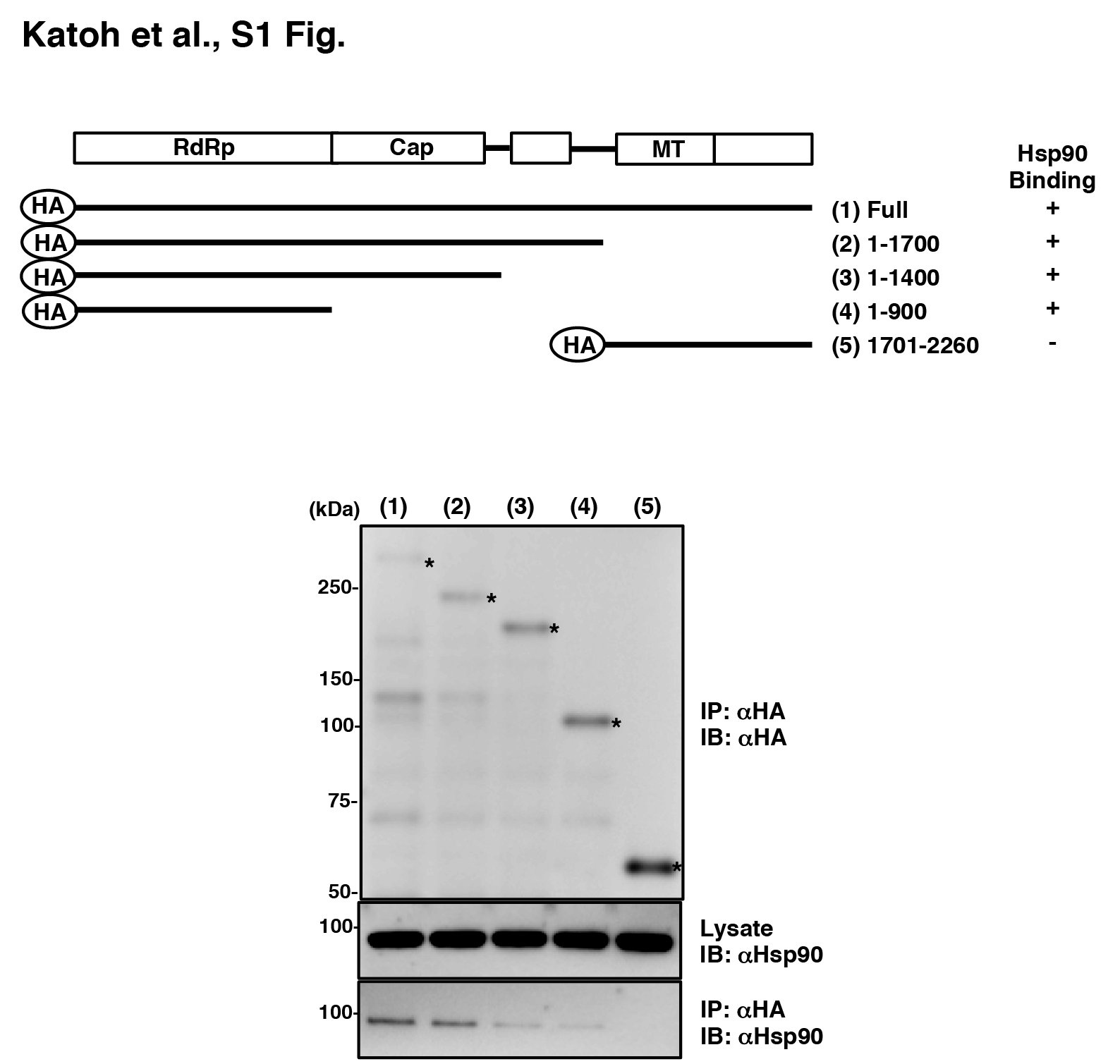

Supplement: S1 Fig — A series of truncated mutants of HA-tagged MuV L protein were expressed in 293T cells, immunoprecipitated with anti-HA antibody, and immunoblotted with anti-HA and anti-Hsp90 antibodies. Asterisks indicate the bands corresponding to each mutant. (TIF) [file ppat.1007749.s001.tif]

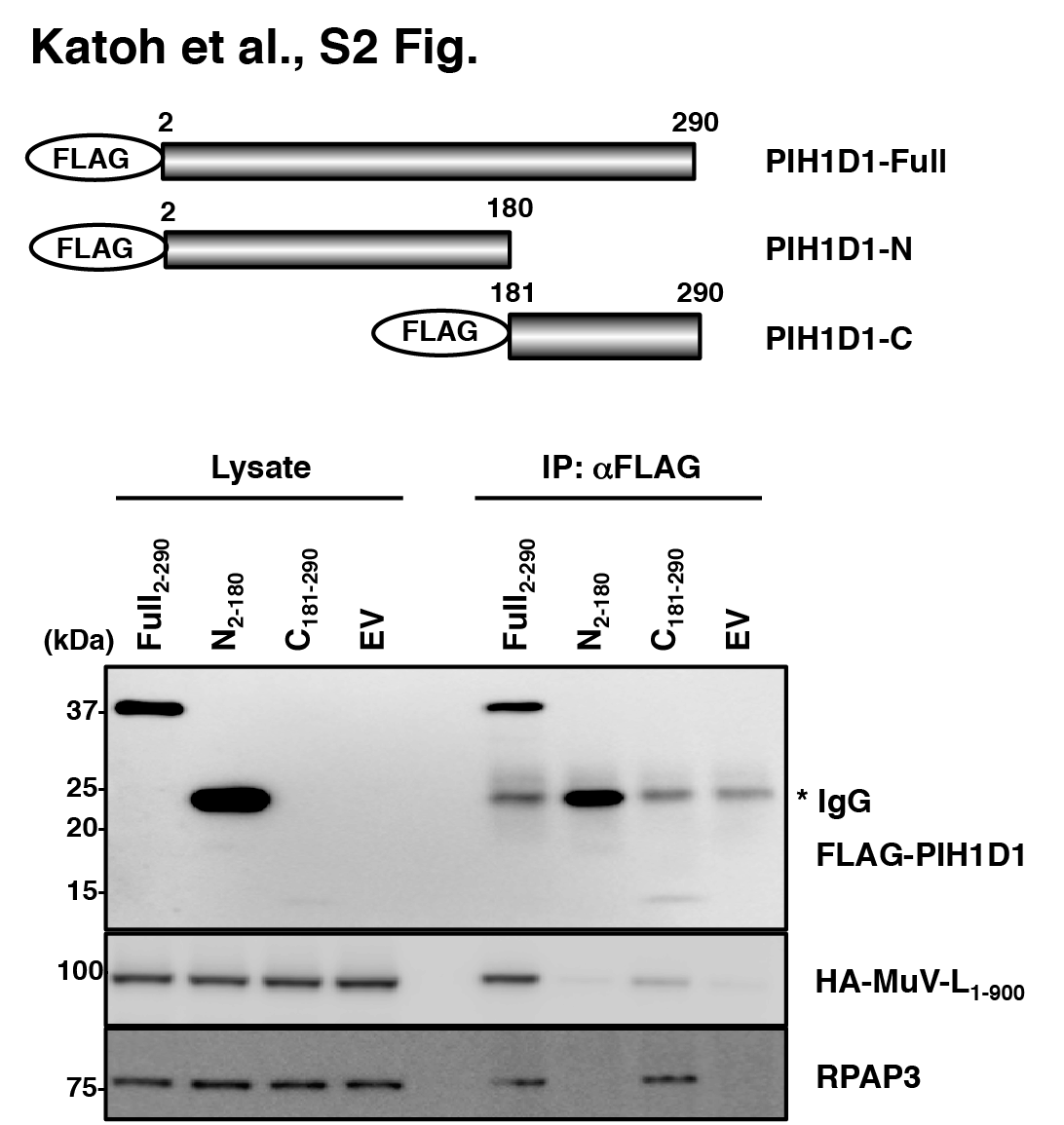

Supplement: S2 Fig — FLAG-PIH1D1 and its truncated mutants were co-expressed with HA-MuV-L1-900 in 293T cells, immunoprecipitated with anti-FLAG antibody and immunoblotted with the indicated antibodies. (TIF) [file ppat.1007749.s002.tif]

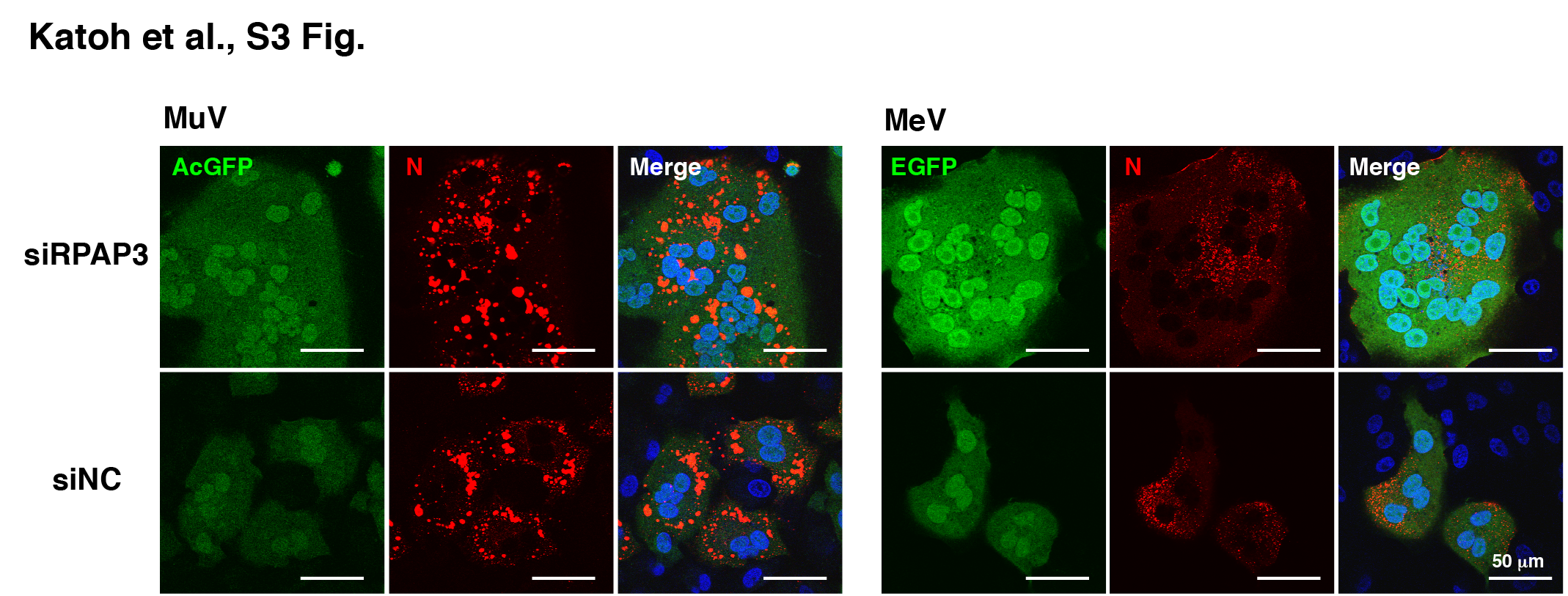

Supplement: S3 Fig — At 48 h post-transfection with either siRPAP3 or siNC, A549 and A549/hSLAM cells were infected with rMuV/AcGFP and rMeV/EGFP, respectively, at an MOI of 0.05. At 48 hpi, cells were stained with rabbit anti-MuV N or mouse anti-MeV N antibody and AF594-conjugated anti-rabbit or -mouse IgG. The cell nuclei were stained with DAPI (blue). (TIF) [file ppat.1007749.s003.tif]

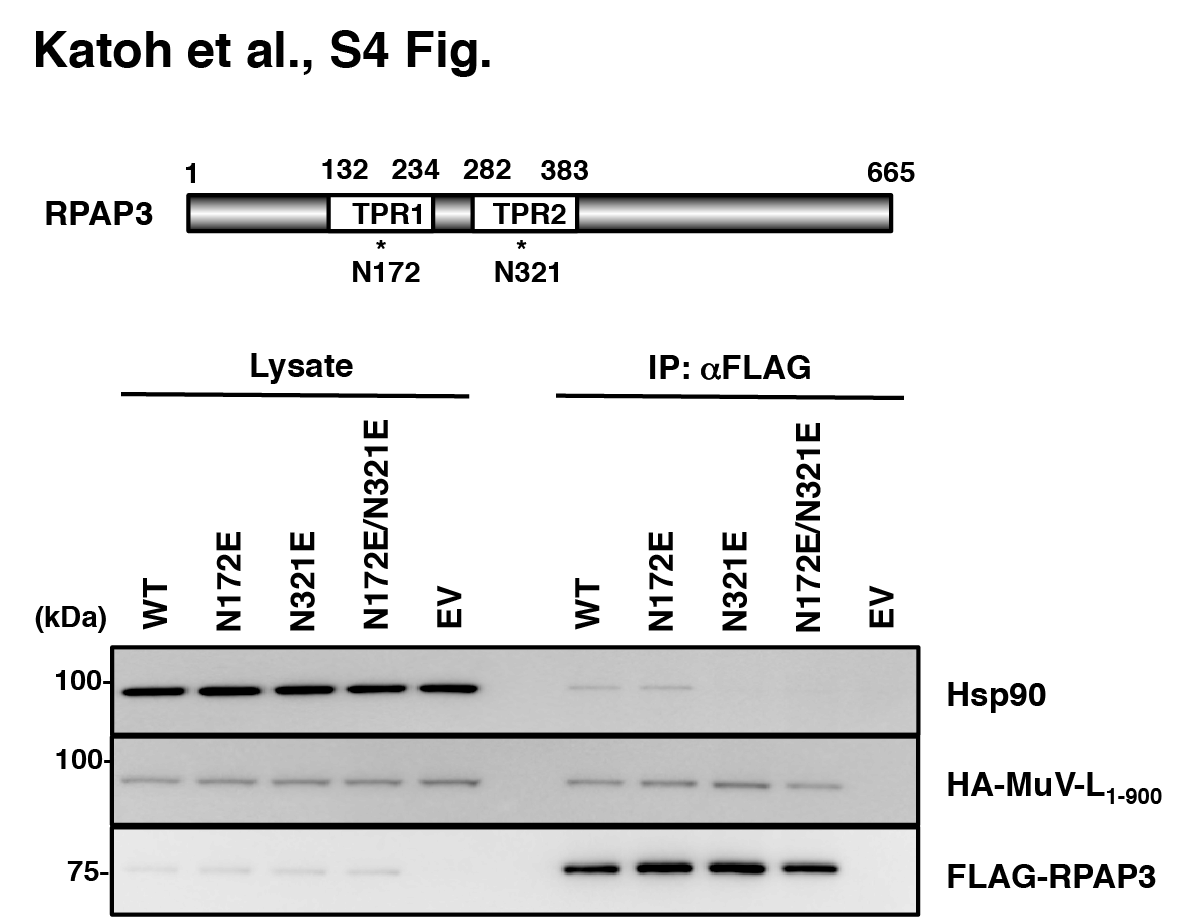

Supplement: S4 Fig — FLAG-RPAP3 and its mutants were co-expressed with HA-MuV-L1-900 in 293T cells, immunoprecipitated with anti-FLAG antibody and immunoblotted with the indicated antibodies. (TIF) [file ppat.1007749.s004.tif]

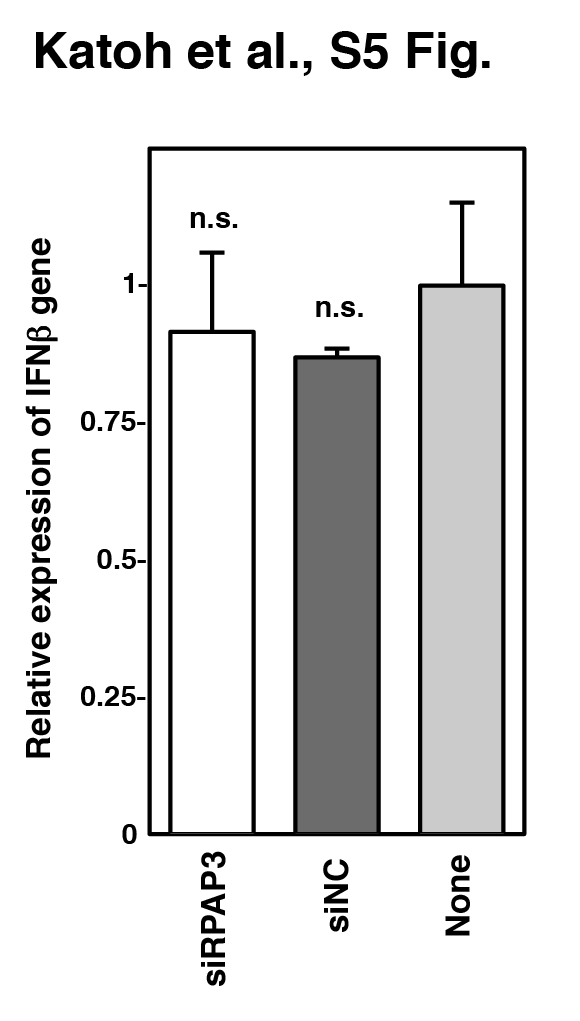

Supplement: S5 Fig — The levels of IFNβ mRNA in A549 cells at 48 h post-transfection with either siRPAP3 or siNC were measured relative to their expression in untransfected cells and normalized to levels of HPRT1 mRNA. Error bars indicate the standard deviations of triplicate wells. The significance of differences between means was determined using the Student’s t-test. n.s. = not significant. (TIF) [file ppat.1007749.s005.tif]

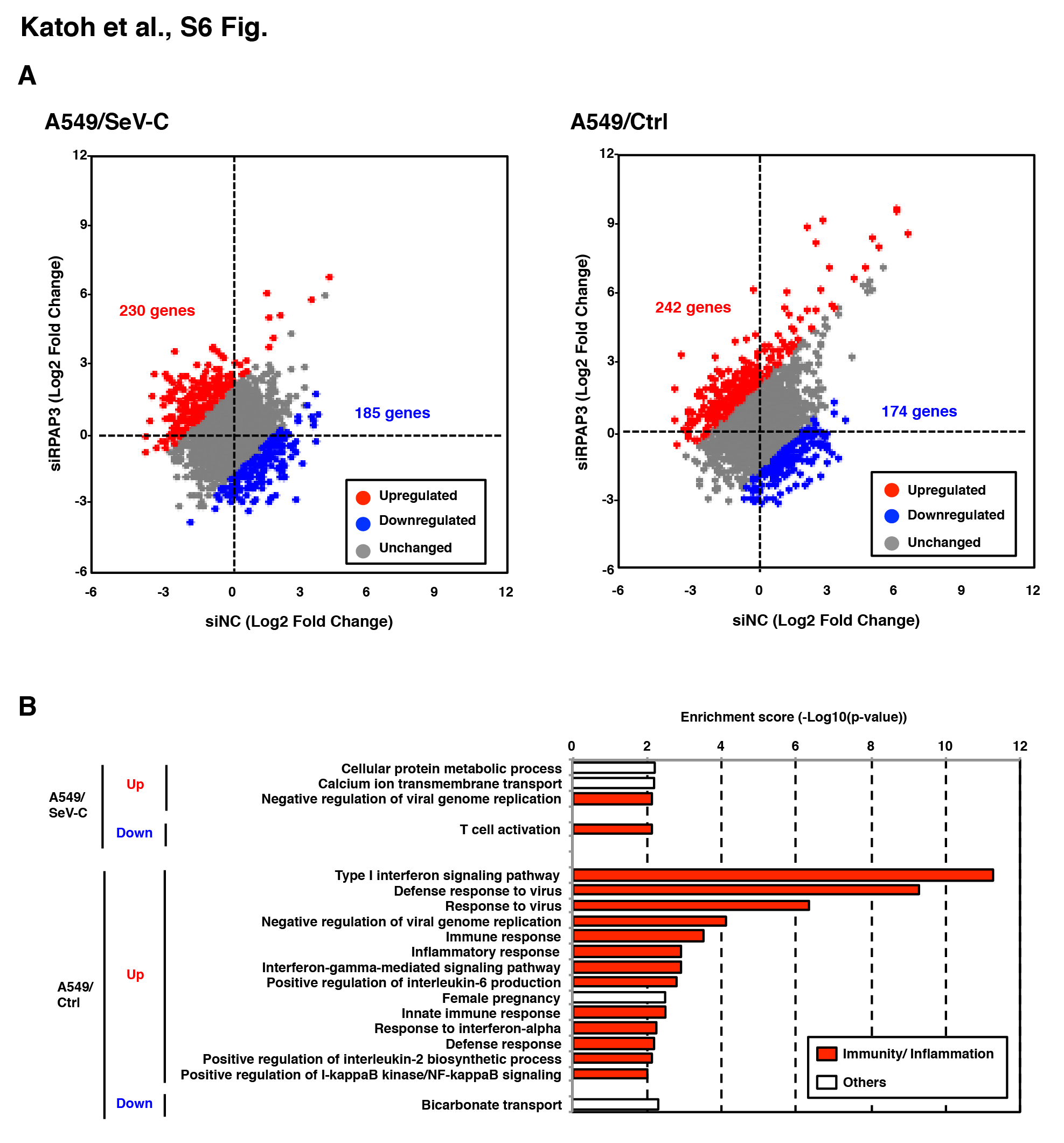

Supplement: S6 Fig — At 48 h post-transfection with either siRPAP3 or siNC, A549/SeV-C and A549/Ctrl cells were infected with rMuV/AcGFP at an MOI of 0.05. (A) Scatter plots of differential gene expression following MuV infection at 48 hpi in RPAP3-knockdown and control cells. Each dot represents the mean expression value (log2-fold change of MuV-infected versus mock cells). x-axis: control cells, y-axis: RPAP3-knockdown cells. Genes with altered expression (>4-fold change) are colored in red and blue for upregulation and downregulation, respectively. (B) GO enrichment analysis of genes with altered expression (>4-fold change) between RPAP3-knockdown and control cells involved in the indicated biological processes. GO categories associated with immunity and inflammation are colored in red. (TIF) [file ppat.1007749.s006.tif]
