# Supplementary material for: The R2TP complex regulates paramyxovirus RNA synthesis
Source: PLoS Pathog. 2019 May 23;15(5):e1007749. doi: 10.1371/journal.ppat.1007749 (PMC6532945; doi:10.1371/journal.ppat.1007749)
Supplement: S1 Table — (PDF) [file ppat.1007749.s007.pdf]

S1 Table. List of differentially expressed genes between MuV-infected and uninfected RPAP3-knockdown A549 cells

Gene name	Fold Change	FDR
AC090360.1	#DIV/0!	0.009511932
CSAG1	#DIV/0!	0.007020838
NAT8	#DIV/0!	0.003670199
RIPOR2	#DIV/0!	0.001511902
CLEC17A	#DIV/0!	4.7885E-05
RAET1L	#DIV/0!	1.38795E-05
IRF4	#DIV/0!	7.54993E-06
IL1RN	#DIV/0!	4.45263E-06
IFNL2	#DIV/0!	1.57019E-08
IFNB1	#DIV/0!	1.8282E-13
ZBP1	#DIV/0!	1.35282E-13
CXCL10	#DIV/0!	0
RTP4	#DIV/0!	0
XAF1	1117.723404	0
IFIT1	922.3819095	0
IFIT2	843.611465	0
MX2	703.1296296	0
IDO1	539.1914894	0
RSAD2	433.6024096	0
IFIT3	419.6507177	0
OAS2	399.8915929	0
IFI27	241.1197605	0
GBP4	231.9519231	0
CMPK2	196.0588235	0
IFITM1	194.5734266	2.32287E-07
SLC15A3	193.7307692	0
MX1	183.3452621	0
IFNL1	180.1764706	8.11015E-11
CCL5	161.8430657	0
CXCL11	151.1315789	0
IFNL3	134.7058824	0
OASL	116.2774529	0
GBP5	112.5555556	0
LAMP3	97.26842105	0
ACKR4	92.22222222	0
APOL3	84.09090909	0
ISG15	82.15704225	0
IFI44L	62.29475983	0
IFI6	60.53776529	0
RUFY4	55.33333333	1.44781E-06
IFI44	54.48872951	0
DHX58	48.86184211	0
TNFSF13B	47.03367003	0
EPSTI1	46.4939759	0
TNFSF10	46.45027125	0
TRIM22	43.74305556	0
BST2	42.67924528	0
BATF2	41.91226321	0
TAC3	39.55555556	1.78733E-07
CLEC7A	38.34146341	3.00842E-13
THEMIS2	37.2716763	0
IL7	36.22222222	1.10427E-06
PARP10	35.13292254	0

HSH2D	34.30487805	0
SERPING1	33.64864865	2.04358E-12
SAMD9L	33.51830331	0
LMO2	32.7875	0
IFIH1	32.30975143	0
PLEKHA4	32.21813031	0
HERC5	32.11988425	0
IRF7	31.37837838	0
IFNL4	29.77777778	1.53608E-05
LGALS9	28.18238213	0
DDX58	27.75009563	0
ETV7	24.76	1.10046E-12
SMTNL1	24.7027027	0
CH25H	24.03125	8.1926E-10
UBA7	23.93897638	0
IL6	23.52941176	1.74241E-07
HELZ2	22.88785543	0
SAMD9	22.05043647	0
DAPP1	21.66666667	0.000611138
OR52K1	21.33333333	0.000979921
CD274	21.14149444	0
PDZD2	20.16071429	0
ANGPTL1	20	1.07408E-07
SOCS1	19.6744186	0.003899314
SLFN12	19.66666667	0.001143526
GBP1	18.64685004	0
DDX60	17.44259078	0
IFITM3	17.10994475	0
APOL1	16.77637691	0
HLA-F	16.65595716	0
OAS3	16.60328889	0
RBM11	16.3	3.54757E-06
USP18	15.97769417	0
TRANK1	15.97587131	0
RARRES3	15.95238095	0
BTK	15.77777778	0.005363981
CARD16	15.77777778	0.005363981
ISG20	15.45179063	0
SP110	15.42598823	0
OAS1	15.03851612	0
CASP1	14.18253968	0
RASGRP3	13.96827411	0
TMEM140	13.87072243	0
CYP2J2	13.65420561	0
IFI16	13.27643361	0
TNFRSF14	13.05882353	0.003255031
KLK10	12.59183673	0.000358529
PDCD1LG2	12.53333333	3.10226E-09
DDX60L	12.42955801	0
GMPR	12.35443038	0
MMP13	12.2962963	1.4888E-06
APOL6	11.87709237	0
CD74	11.87037037	4.91172E-10
NLRC5	11.85627155	0
RET	11.82080925	0

HRASLS2	11.6	1.66793E-12
PARP12	11.36370132	0
PARP14	11.29415654	0
FNDC11	10.65957447	2.18527E-07
STAT2	10.50234632	0
IRF9	10.49759846	0
TREX1	10.42936288	0
IFI35	10.2302287	0
TLR3	10.22494514	0
PPM1K	10.12150943	0
CACNA1I	10.11330049	0
HLA-B	10.09342505	0
HCAR3	10.01360544	0
ACE2	9.945454545	8.32187E-09
PARP9	9.877995016	0
CFB	9.86625	4.66311E-13
C5orf56	9.85	0
APOBEC3H	9.352941176	0.008586987
ATP4A	9.285714286	0.00021113
HERC6	9.099392206	0
IQSEC3	8.576923077	0.007532881
DTX3L	8.559987615	0
STAT1	8.4621555	0
PIK3AP1	8.419733118	0
C19orf66	8.208147895	0
GBP3	7.976889745	0
TNFRSF1B	7.948905109	4.56182E-08
NT5C3A	7.935234562	0
IRF1	7.760188564	0
NOD2	7.75	0.001311196
UBE2L6	7.740030522	0
CX3CL1	7.653688525	1.34249E-11
GLI3	7.638297872	1.65316E-05
ACAN	7.633333333	0.000974889
TRIM21	7.567471144	0
CAMK4	7.441558442	2.56578E-08
C12orf74	7.397727273	2.24821E-06
C3AR1	7.3	3.81276E-07
HCAR2	7.267692308	0
PML	7.151351351	0
PLEKHG7	7.151162791	0.004747729
PATL2	7.063829787	5.79781E-05
LAP3	7.050617947	0
GIMAP2	6.888429752	0
IL15RA	6.844990548	0
DUOX2	6.818181818	2.46857E-06
BCL2L14	6.666666667	0.00038852
CEACAM1	6.516951581	0
SAMHD1	6.504540024	0
FRMD3	6.484375	0
SP100	6.388309275	0
CD34	6.321428571	0.007841801
PLSCR1	6.30703125	0
ADAMTS20	6.266666667	1.84239E-06
SYNE3	6.264	1.70739E-10

LAPTM5	6.2	0.000918172
TRIM14	6.19799415	0
PNPT1	6.052016985	0
DDO	6.038961039	3.49626E-09
NMI	5.926165803	0
PSMB9	5.840242669	0
TAP1	5.821734187	0
APOL2	5.799380141	0
IFIT5	5.776248781	0
EXOC3L1	5.747663551	4.71724E-08
TDRD7	5.683945416	0
SPTA1	5.657894737	0.003551925
MYD88	5.583565783	0
RNF213	5.541634315	0
NCF2	5.495934959	0
CD68	5.406963891	0
ODF3B	5.255882353	0
ZNFX1	5.250024127	0
IFITM2	5.231143249	0
NCCRP1	5.162162162	0.007832188
IL4I1	5.155165536	0
TAP2	5.060360692	0
BLNK	4.941558442	5.75574E-09
TRIM5	4.830071174	0
UBD	4.811369509	0
IL18BP	4.8	6.62944E-15
APOBEC3F	4.791364003	0
BTN3A3	4.714369847	0
SIDT1	4.706309324	0
PIGR	4.694444444	0.001051396
ID4	4.666666667	0.000125496
HLA-E	4.622286254	0
PRDM16	4.537974684	1.25462E-06
WARS	4.506299158	0
ATP10A	4.504990758	0
C4A	4.48773842	5.97109E-11
FLT3LG	4.466666667	0.000765759
SEC16B	4.369623656	1.66058E-10
C1R	4.362039417	5.67878E-12
IL22RA1	4.296057924	0
EIF2AK2	4.275998217	0
ATF3	4.236231884	0
TRIM25	4.162238941	0
TRIM38	4.144321196	0
CDK18	4.110411622	0
SSTR2	4.049180328	3.07523E-05
TGM2	4.005197953	0
GPM6A	4	4.67265E-07
MAP2	3.966518255	0
PHF11	3.965588438	0
ACSL5	3.943396226	4.1729E-10
HES4	3.939491917	0
ACY3	3.929906542	0.001107844
TMEM173	3.882160393	4.66201E-08
PI4K2B	3.877151052	0

CDH3	3.833333333	0.006304543
HLA-C	3.82915441	0
ZNF107	3.8248	0
BTC	3.810045662	0
RGS22	3.778501629	5.99008E-11
TMEM229B	3.755454545	0
RHEBL1	3.733041575	6.21822E-08
ICAM1	3.70246085	1.37258E-11
KIAA2012	3.701923077	0.000722092
HMX1	3.701298701	1.54257E-05
TYMP	3.642480211	0
PSMB8	3.632344214	1.94509E-13
MT2A	3.626246362	0
IL2RG	3.605839416	0.000949728
ERAP2	3.590661479	0
C4B	3.550077042	9.99303E-07
CD38	3.5105763	0
TRIM69	3.492829205	0
OGFR	3.3960047	0
GCNT4	3.357963875	0.002688866
NCOA7	3.357027263	0
AC006978.2	3.325892857	0.004443628
BLZF1	3.305832148	0
SP140L	3.304837238	0
ADAR	3.295648036	0
KLF4	3.282962963	0
PLA1A	3.241029641	0
PMAIP1	3.23365929	0
PSMB10	3.216807368	0
C2CD4A	3.159793814	0.000352123
DHRS2	3.137690355	0
B2M	3.123637573	0
FAM71F2	3.123076923	0.00861744
ADGRE1	3.115460993	0
JAK2	3.108093574	1.04493E-10
TRAF1	3.076368876	0
FYB1	3.073132104	0
SLC25A28	3.06972973	0
C8orf46	3.0625	0.008850544
CMTR1	3.051549809	0
AL669918.1	3.034213945	0.000282235
AC134772.2	3.015389447	0
SEMA3D	3.005003574	1.30228E-14
SSC5D	3	0.003762603
EBF4	2.996784566	1.32995E-06
MUC16	2.990338164	8.17747E-05
BTN3A1	2.968835023	0
EHD4	2.950853443	0
IFI30	2.934924623	0.00216989
TRIM31	2.922163588	1.30972E-07
PLAUR	2.916258795	0
EGR1	2.912524851	0
CRYBG1	2.892624729	1.29186E-08
ACVRL1	2.87804878	3.28231E-05
OPTN	2.871486805	0

TMEM106A	2.853556485	4.34318E-05
RNF19B	2.851042139	0
CTSS	2.847124452	0
NFE2L3	2.846059808	0
DPP4	2.844660194	0.000989558
RICTOR	2.841646384	0
BCO1	2.822198657	0
FZD4	2.817777778	0.000240923
STARD5	2.8	0.00050692
LY6E	2.775366457	0
XDH	2.766763848	0.000688023
IRF2	2.762736803	0
PCDH17	2.730769231	0.001240463
NUPR1	2.725280326	0
MASTL	2.724376283	0
TRAFD1	2.718175182	0
CASP4	2.7145615	0
FAM122C	2.7	0.000387569
VCAM1	2.698324022	0.000280729
ZNF804A	2.692482916	2.35231E-08
GTPBP1	2.674604966	0
MLKL	2.674000537	0
FAM46A	2.673007246	8.37616E-12
C15orf48	2.626415094	0
MUC13	2.624419096	0.001579289
SLC8A2	2.620754717	8.84024E-06
LHX9	2.609137056	0.000403057
RPH3A	2.596339114	5.11729E-09
TMEM62	2.595431098	0
KCNT2	2.593415008	5.05E-14
LGALS3BP	2.562770513	0
PARP8	2.547224927	6.62944E-15
C1QL1	2.541357673	1.85386E-06
TMEM92	2.537596627	0
TTC39B	2.533659066	3.80265E-14
LIFR	2.526608464	0
PCDH1	2.520076482	0
CASP7	2.513716922	0
PDGFRL	2.507518797	0
MXD1	2.501116902	0
C4orf33	2.493757803	0
HIP1R	2.470930233	0
ZCCHC2	2.467072496	0
XRN1	2.452873563	0
TRIM26	2.440349345	0
FGF2	2.437266202	0
PSME2	2.435477515	0
CACNG6	2.433827422	7.83945E-12
MDK	2.428959276	1.34309E-06
C1S	2.418167255	0
NKX3-1	2.417354009	0
SPATS2L	2.41410038	0
CLEC2B	2.411380597	0
TXNIP	2.404736276	0.001305783
CHSY3	2.4	0.006769297

REC8	2.395789474	0.000264777
HLA-A	2.395356621	0
ARHGEF6	2.388392857	0.002929526
NAPA	2.381408938	0
FST	2.379920889	0
HYAL4	2.364253394	2.89302E-05
ZC3HAV1	2.363376635	0
CNP	2.362744584	0
RBM43	2.355651548	0
PCDHAC2	2.349862259	0.000102481
PRKD2	2.346918971	0
CASZ1	2.343347639	1.35282E-13
AGBL2	2.338709677	1.54767E-05
PRR15	2.334926471	0
CADPS2	2.331809872	0.003418471
KIAA1217	2.325312574	0
NR4A2	2.314132619	0.000199183
SCIN	2.311904762	8.50373E-09
ST8SIA4	2.296168268	5.39882E-13
ASPHD2	2.290843806	0.000552168
MDGA1	2.269458988	0
CSF1	2.269195861	0
CACNG8	2.264787752	0
HRH2	2.262948207	3.38825E-08
STARD4	2.262073599	0
AL136295.5	2.259509918	0
SCO2_2	2.256944444	2.59261E-06
ENDOD1	2.252964427	0
STS	2.243816794	0
ERAP1	2.236047031	8.77405E-09
TAPBPL	2.216720488	0.000104206
TNFAIP3	2.216568993	0
ARL14	2.209076175	0.00780583
TRIM56	2.196172778	0
TMC8	2.192678227	0.002762155
BCL2L13	2.189527521	0
SIX1	2.188644689	0
NUB1	2.187184009	0
PRDM1	2.184848485	1.96755E-07
CAVIN2	2.181802692	0
ETS2	2.172181676	0
KLF6	2.168675601	0
BANK1	2.159863946	7.82892E-11
CEL	2.158273381	0.004454737
CHMP5	2.15672174	0
MOV10	2.155239327	0
CLDN4	2.154801325	4.40383E-07
C17orf67	2.153846154	7.33495E-07
CSRNP1	2.152965171	0
SLFN5	2.143247529	0
HEG1	2.142936685	0
NTNG2	2.134556575	2.36877E-07
JADE2	2.133630359	0
GABRE	2.128735632	0
ZFYVE26	2.124915916	0

HDAC9	2.121813871	0
GNB4	2.118793865	0
MAPK8IP2	2.117421008	0
FAM135A	2.109825561	0
CASP10	2.105932203	0
RNF114	2.102910072	0
ANKFY1	2.094519264	0
RUBCN	2.094057601	0
AC073111.5	2.086894587	4.34546E-06
SQOR	2.085755658	0
DSP	2.079366874	0
ZBTB32	2.078817734	0.001173138
MSX1	2.078584283	2.06402E-13
SERPINE1	2.076814337	0
VASH2	2.072908672	0.000109784
FMR1	2.052194678	0
HNF4G	2.044732195	0.009822721
TCF4	2.044498382	0
ZFP36L2	2.033050306	2.60342E-10
PROX1	2.03195963	4.92247E-05
GBP2	2.020646021	0
DCP1A	2.020278271	1.30228E-14
HTR1F	2.014204545	0.004029866
AC008695.1	2.010460251	0.003883337
PSME1	2.002629017	0
CCDC157	-2	0.004023505
SLC27A3	-2	0.00174762
RPGRIP1	-2	0.007097371
MEIOB	-2.021857923	0.001594683
HIST1H4L	-2.030523256	0.003372656
CMTM4	-2.048869681	4.37822E-22
CT45A3	-2.056206089	3.96135E-07
ODC1	-2.064213862	4.7937E-183
RTEL1-TNFRSF6B	-2.103603604	2.73355E-06
GPATCH11	-2.113624679	1.74671E-17
AC004922.1	-2.113861386	1.06525E-15
AC124312.1	-2.127408994	9.2159E-09
NIPAL4	-2.159541189	2.94734E-09
NUP62	-2.168198395	6.83623E-16
SLC4A8	-2.189759036	3.46012E-07
CASTOR2	-2.195134062	1.0461E-21
MAGEA3	-2.23255814	0.006528239
PNPLA3	-2.233108108	0.000986992
NDUFC2-KCTD14	-2.251968504	0.003796607
HSPE1-MOB4	-2.255874674	0.002781898
ACO1	-2.256123919	1.61476E-92
RFLNB	-2.264496718	1.93356E-28
CLN8	-2.265639652	1.20401E-82
FAM172A	-2.271989614	2.58146E-31
LONRF2	-2.370286576	6.60335E-39
TMEM170B	-2.412572856	2.22536E-30
HIST1H2AH	-2.412694013	4.66311E-13
MGAT4C	-2.413043478	0.002797949
ATRIP	-2.443316413	0.002822723
FAM171A1	-2.478305785	5.78674E-60

HIST2H3D	-2.581735694	1.04301E-10
CERK	-2.596866451	1.91275E-64
ANKRD2	-2.611464968	0.00373951
AL136531.2	-2.632432432	0.001175629
HIST2H2AA3	-2.705985915	0.000644062
KCNAB1	-2.712643678	1.34045E-06
HIST1H2AI	-2.743869698	2.82213E-29
NPBWR1	-2.774319066	0.001086756
NT5DC2	-2.807153966	3.57613E-88
NUPR2	-2.885964912	0.005279191
C21orf33	-2.892241379	7.30337E-06
FAM173B	-2.94312455	1.21019E-07
AC004754.1	-3.25	0.003723506
CDK14	-3.277108434	7.25514E-05
AC105001.2	-3.284313725	0.002578744
HIST1H4B	-3.299604296	2.91801E-64
MAP1LC3B2	-3.37037037	0.005772214
TNFRSF10D	-3.513200258	7.95524E-81
CYP26A1	-3.561643836	0.007044345
ZMAT3	-3.976365816	9.1239E-236
GCOM1	-4.015503876	2.89553E-05
FOXD4	-4.036144578	0.00978334
BEST1	-4.264150943	0.00516954
PAGR1	-4.363963964	2.22222E-27
MISP3	-6.212121212	7.40217E-05
UGT2A3	-11.27272727	0.00931714
KCNJ3	-13.77777778	0.00931714
GFRA2	-16.11111111	0.003669487
