# Supplementary material for: The R2TP complex regulates paramyxovirus RNA synthesis
Source: PLoS Pathog. 2019 May 23;15(5):e1007749. doi: 10.1371/journal.ppat.1007749 (PMC6532945; doi:10.1371/journal.ppat.1007749)
Supplement: S2 Table — (PDF) [file ppat.1007749.s008.pdf]

S2 Table. List of differentially expressed genes between MuV-infected and uni

Gene name	Fold Change	FDR
IFNL2	#DIV/0!	0.002963657
MX2	#DIV/0!	0
XAF1	388.7	0
OAS2	163	0
MX1	150.7529976	0
BST2	102.8	0
RSAD2	84.6	0.006394263
IFI44L	68.38194444	0
IFIT1	65.96085409	2.92562E-07
IFITM1	61.36734694	0
CMPK2	49.30128205	0
IFI44	38.40569395	7.15498E-12
IFI6	38.15058236	0
OASL	29.47826087	0
CCL5	28.14285714	0
IFIT3	27.94329897	8.13453E-12
GBP4	26.26315789	4.41884E-07
IFNL1	24.10526316	2.53932E-06
ISG15	23.61281337	2.34808E-06
IRF7	18.93163539	0
IFIT2	13.66367713	0
LAMP3	11.73390558	2.40668E-08
PARP10	10.97236181	0.000152715
DDX58	10.94764279	0
BATF2	10.94408602	0
HELZ2	9.334731544	1.61794E-10
IFIH1	8.774224806	0
OAS1	8.237070547	0
SP110	7.802476415	0
DHX58	7.629310345	7.93347E-07
EPSTI1	7.629310345	2.78723E-08
SAMD9L	7.461461461	0
LMO2	7.448275862	0.001029165
DHRS2	7.176470588	0
PARP9	7.135963581	0
IDO1	6.944444444	1.62727E-09

HSH2D	6.911764706	9.25439E-10
SAMD9	6.722039674	0
DDX60	6.420707255	0
IRF9	5.996411483	0
PARP12	5.427876429	0
OAS3	5.400982563	0
DTX3L	5.26649367	0
IFI16	5.1839585	0
HERC5	4.814903272	0
REC8	4.745257453	0.000973266
NLRC5	4.398411957	0
C19orf66	4.278516058	0
LGALS9	4.243076923	3.15676E-07
TRANK1	4.080364428	0
TRIM22	3.667221298	9.48381E-06
USP18	3.653339718	0
DDX60L	3.613108995	0
GBP1	3.592970522	0
STAT2	3.44364768	0
PPM1K	3.371134021	7.76158E-08
PARP14	3.346059866	0
TRIM14	3.340502355	0
IFI35	3.277123373	1.17202E-09
APOL6	3.247795993	0
PLEKHA4	3.226111637	3.51012E-05
TRIM21	3.191977077	0
AC074143.1	3.18879056	1.03484E-05
HLA-F	3.14893617	6.78007E-06
HERC6	3.145219124	0
TNFSF10	2.965669014	9.80246E-08
IFIT5	2.901075986	0
SAMHD1	2.883967322	0
PML	2.752123271	0
STAT1	2.736244929	0
EIF2AK2	2.723247897	0
TLR3	2.613783534	0
APOL1	2.594715296	2.05254E-08
IFITM3	2.564498346	0.000266021

SP100	2.537380383	0
TRIM25	2.519433985	0
PIK3AP1	2.45645585	0
PLSCR1	2.411508045	0
ZNFX1	2.392871816	0
AC092718.3	2.336520076	0.005612371
UBE2L6	2.326741464	1.05132E-10
PNPT1	2.314231192	3.08309E-08
PHF11	2.308383234	0.001793255
RNF213	2.141561249	0
CEACAM1	2.135227731	0
IL22RA1	2.047475509	4.99752E-07
MYD88	2.045167029	0
LAP3	2.037811118	0
AL139011.2	2.029090909	0.000119231
AC138811.2	-2.380053908	0.003393093
TLR10	-2.646666667	0.000225404

nfected control A549 cells
