# Supplementary material for: The R2TP complex regulates paramyxovirus RNA synthesis
Source: PLoS Pathog. 2019 May 23;15(5):e1007749. doi: 10.1371/journal.ppat.1007749 (PMC6532945; doi:10.1371/journal.ppat.1007749)
Supplement: S3 Table — (PDF) [file ppat.1007749.s009.pdf]

S3 Table. List of genes upregulated or downregulated following MuV infection in RPAP3-knockdown A549 cells

Gene name	Fold Change (MuV vs Mock)		Fold Change (siRPA3 vs siNC)
	siNC	siRPAP3	
ACKR4	0.71428571	92.2222222	129.111111
GBP5	1	112.555556	112.555556
IDO1	6.94444444	539.191489	77.6435745
IFIT2	13.6636771	843.611465	61.7411738
IL6	0.40816327	23.5294118	57.6470588
SOCS1	0.36764706	19.6744186	53.5144186
TNFSF13B	1.17448405	47.03367	40.0462398
CLEC7A	1.06896552	38.3414634	35.8678206
TMEM200C	0.20833333	7.11764706	34.1647059
OVOL3	0.17241379	5.54545455	32.1636364
IFNL4	1	29.7777778	29.7777778
POU3F3	0.3125	9.22222222	29.5111111
ENPP3	0.16949153	5	29.5
NPFFR2	0.10416667	3.02222222	29.0133333
HOXA9	0.14018692	4	28.5333333
GLDN	0.5	13.6666667	27.3333333
OR52K1	0.78947368	21.3333333	27.0222222
SAA1	0.33333333	8.72727273	26.1818182
APOBEC3H	0.36764706	9.35294118	25.44
AC068547.1	0.17391304	4.14705882	23.8455882
TICAM2	0.51724138	12.1428571	23.4761905
APOL3	3.67241379	84.0909091	22.897994
SLFN12	0.89690722	19.6666667	21.9272031
CD34	0.28971963	6.32142857	21.8191244
TP53TG3B	0.12121212	2.60714286	21.5089286
CXCL11	7.1	151.131579	21.2861379
PTH1R	0.12820513	2.72727273	21.2727273
NOXO1	0.20408163	4.23529412	20.7529412
RHBDL2	0.12987013	2.55319149	19.6595745
APOBEC3A	0.14925373	2.90909091	19.4909091
XIRP2	0.25641026	4.90909091	19.1454545
GSDMC	0.12820513	2.39473684	18.6789474
PATE4	0.2238806	3.90909091	17.4606061
IPCEF1	0.51948052	8.88888889	17.1111111
PDZD2	1.19852941	20.1607143	16.8212095
ZNF790	0.25641026	4.09090909	15.9545455
TNFSF10	2.96566901	46.4502712	15.662662
IFIT3	27.943299	419.650718	15.0179375
CXCL17	0.17241379	2.55555556	14.8222222
ERVW-1	0.14705882	2.12658228	14.4607595
CD74	0.82352941	11.8703704	14.4140212
IL7	2.55	36.2222222	14.204793
IFIT1	65.9608541	922.38191	13.9837775
THEMIS2	2.70229008	37.2716763	13.792626
C5orf56	0.73931624	9.85	13.3231214
WDR49	0.56349206	7.35	13.043662
NPB	0.22058824	2.76190476	12.5206349
DAPP1	1.75	21.6666667	12.3809524
ADAMTS20	0.51470588	6.26666667	12.1752381
SHISA8	0.36458333	4.4	12.0685714
NUGGC	0.64102564	7.64705882	11.9294118
TRIM22	3.6672213	43.7430556	11.92812

DDO	0.51020408	6.03896104	11.8363636
HRASLS2	1	11.6	11.6
PURG	0.09345794	1.08163265	11.5734694
CCL3L3	1	11.5555556	11.5555556
SLC15A3	16.8	193.730769	11.5315934
POU3F2	0.3125	3.6	11.52
TNFSF18	1	11.3636364	11.3636364
ADAM29	1	11.1818182	11.1818182
MYLK4	0.14367816	1.59375	11.0925
RARRES3	1.45534151	15.952381	10.961263
FLT1	0.82352941	9	10.9285714
GMPR	1.14375	12.3544304	10.8016878
BICDL2	0.36764706	3.76470588	10.24
CFAP58	0.12820513	1.30120482	10.1493976
VAMP5	0.83333333	8.44444444	10.1333333
PLEKHA4	3.22611164	32.2181303	9.98667558
FGF17	0.20833333	2.04761905	9.82857143
ISG20	1.59762587	15.4517906	9.67172035
CD274	2.19230769	21.1414944	9.64348869
SLC26A4	0.20833333	2	9.6
PCDHB7	0.78947368	7.54545455	9.55757576
PATL2	0.75	7.06382979	9.41843972
KIAA1456	0.22058824	2.06976744	9.38294574
CAPN3	0.36764706	3.42857143	9.32571429
ATP12A	0.11494253	1.07142857	9.32142857
EDAR	0.38461538	3.54545455	9.21818182
HCAR3	1.08965517	10.0136054	9.1897012
IZUMO4	0.64102564	5.88888889	9.18666667
SEMA4A	0.44871795	4.07142857	9.07346939
CCNI2	0.53448276	4.76470588	8.91461101
B3GNT8	0.36787565	3.26923077	8.88678223
ACP4	0.17241379	1.52380952	8.83809524
GBP4	26.2631579	231.951923	8.83183675
ATP4A	1.05172414	9.28571429	8.82903981
FZD4	0.32270916	2.81777778	8.73163237
SYNE3	0.71891892	6.264	8.71308271
WISP1	0.76923077	6.7	8.71
AC007998.2	0.20833333	1.8125	8.7
FXYD3	0.60344828	5.20930233	8.63255814
SPATA9	0.26315789	2.22222222	8.44444444
AC093525.1	0.11494253	0.96226415	8.37169811
ABAT	0.14705882	1.22666667	8.34133333
LAMP3	11.7339056	97.2684211	8.28951796
BHLHE22	1	8.27272727	8.27272727
RNASE1	0.5	4.10714286	8.21428571
GRM4	0.25641026	2.07246377	8.0826087
UBA7	2.96221662	23.9389764	8.08144015
CPA6	0.20408163	1.64864865	8.07837838
ADAM20	0.72916667	5.88888889	8.07619048
THBS4	0.52631579	4.22222222	8.02222222
SSPN	0.51282051	4.05263158	7.90263158
CARD16	2	15.7777778	7.88888889
RASGRP3	1.78370625	13.9682741	7.83103949
AC068775.2	0.34482759	2.7	7.83
LRRN3	0.64102564	5	7.8

CLC	1	7.77777778	7.77777778
EGR3	1.05263158	8.09090909	7.68636364
C20orf141	1	7.52941176	7.52941176
IFNL1	24.1052632	180.176471	7.47456974
ID4	0.62580645	4.66666667	7.45704467
DLEC1	0.5	3.71428571	7.42857143
ANGPTL1	2.7	20	7.40740741
PLEKHG7	0.96551724	7.15116279	7.40656146
SERPING1	4.55882353	33.6486486	7.3809939
TREX1	1.42748092	10.4293629	7.30613122
AC091167.7	0.25423729	1.82352941	7.17254902
ZCWPW2	0.20618557	1.47272727	7.14272727
PRF1	0.5	3.55555556	7.11111111
AL049844.1	0.20408163	1.42857143	7
TACR1	0.64102564	4.45	6.942
ALOX15	1.16666667	8.09090909	6.93506494
TNFRSF8	1	6.88888889	6.88888889
IGSF9	0.53448276	3.63157895	6.79456706
GUCA1B	0.20618557	1.4	6.79
IFITM3	2.56449835	17.1099448	6.67184862
HERC5	4.81490327	32.1198842	6.67093033
NPY4R2	0.14705882	0.98101266	6.67088608
MYL2	0.5	3.33333333	6.66666667
FOXD4L3	0.25641026	1.70588235	6.65294118
LGALS9	4.24307692	28.1823821	6.64196823
ERP27	0.51724138	3.4	6.57333333
PDGFRB	0.51282051	3.33333333	6.5
INMT-MINDY4	0.52743902	3.42741935	6.4982286
SECTM1	0.72916667	4.73170732	6.48919861
APOL1	2.5947153	16.7763769	6.46559449
CARD11	0.87931034	5.66666667	6.44444444
CACNA1I	1.57419355	10.1133005	6.42443269
C9orf152	0.34482759	2.20930233	6.40697674
DHX58	7.62931034	48.8618421	6.40449004
NKX2-3	0.52631579	3.33333333	6.33333333
BTK	2.5	15.7777778	6.31111111
SLC35D3	0.5	3.11111111	6.22222222
SP8	0.35632184	2.20353982	6.18412789
SOX7	0.11111111	0.68674699	6.18072289
NOS3	0.68041237	4.2	6.17272727
MMP13	2	12.2962963	6.14814815
NCAM1	0.34482759	2.11111111	6.12222222
EPSTI1	7.62931034	46.4939759	6.09412566
NRSN1	0.51282051	3.11111111	6.06666667
TLR2	0.35869565	2.16883117	6.04643841
NPFF	1	6	6
RFPL4A	1	5.88888889	5.88888889
SH2D1B	1	5.88888889	5.88888889
TMEM140	2.35824742	13.8707224	5.88179268
C11orf52	0.25773196	1.5	5.82
HCAR2	1.24953096	7.26769231	5.81633634
C9orf84	0.39655172	2.29411765	5.78516624
CDKL4	0.22988506	1.32885906	5.78053691
SDS	0.52272727	3.01162791	5.76137513
KIAA2012	0.64367816	3.70192308	5.75120192

CCL5	28.1428571	161.843066	5.75076883
MTTP	0.78947368	4.46666667	5.65777778
IL2RG	0.64102564	3.60583942	5.62510949
RET	2.10344828	11.8208092	5.61972899
FAM71F2	0.55747126	3.12307692	5.60222046
C8orf46	0.54679803	3.0625	5.60078829
TP53TG3C	0.68965517	3.81818182	5.53636364
MME	0.5	2.72727273	5.45454545
SDCBP2	0.57142857	3.1	5.425
EYS	0.82075472	4.38461538	5.34217507
SDR42E1	0.26910299	1.43269231	5.32395537
NCCRP1	0.97058824	5.16216216	5.31859132
CCDC153	0.63703704	3.38461538	5.31305903
KIAA0408	0.43231441	2.29124236	5.29994445
HLA-F	3.14893617	16.6559572	5.2893918
PRSS22	0.57731959	3.05	5.28303571
CDRT15	0.64102564	3.33333333	5.2
SATB1	0.83333333	4.33333333	5.2
UGT1A7	0.83333333	4.33333333	5.2
FAM186B	0.46728972	2.42857143	5.19714286
ABCA6	0.2991453	1.55319149	5.19209726
GBP1	3.59297052	18.64685	5.18981437
KCTD14	1.6875	8.72727273	5.17171717
ATP1A2	0.25641026	1.32432432	5.16486486
RSAD2	84.6	433.60241	5.12532399
SLC6A11	0.34482759	1.76470588	5.11764706
ONECUT3	0.52631579	2.68421053	5.1
ABCA9	0.34482759	1.75609756	5.09268293
GLI3	1.5	7.63829787	5.09219858
B4GALNT2	0.53043478	2.69090909	5.07302534
NAP1L3	0.20098039	1.01769912	5.06367365
OCA2	0.4742268	2.4	5.06086957
LY9	0.34482759	1.74193548	5.0516129
VWA2	0.42268041	2.125	5.02743902
ABI3BP	0.78735632	3.95266272	5.02017017
CX3CL1	1.52883263	7.65368852	5.00623049
AOC1	1	5	5
HLA-B	2.02173913	10.0934251	4.9924468
HSH2D	6.91176471	34.304878	4.96325895
LRP2	1.28205128	6.35	4.953
ARL10	0.51020408	2.50632911	4.91240506
MAPK15	0.72916667	3.54545455	4.86233766
NXPH1	0.22058824	1.07142857	4.85714286
ANKUB1	0.52631579	2.55555556	4.85555556
BCL2L14	1.375	6.66666667	4.84848485
PAX5	1.31578947	6.36842105	4.84
JPH1	0.64935065	3.11764706	4.80117647
CDH3	0.79901961	3.83333333	4.79754601
NOS2	0.51546392	2.47115385	4.79403846
FUT4	0.27720207	1.32167832	4.76792367
MYO3A	0.32258065	1.53191489	4.74893617
CACNB2	0.31034483	1.47317073	4.74688347
ARIH2OS	0.48630137	2.3	4.72957746
CTCFL	0.52238806	2.46153846	4.71208791
GAD2	1	4.66666667	4.66666667

CLEC18C	0.91025641	4.22222222	4.63849765
CFAP126	0.58823529	2.72727273	4.63636364
HHLA2	0.5974026	2.76666667	4.63115942
UBE2QL1	1	4.60606061	4.60606061
CCDC110	0.63265306	2.90909091	4.59824047
HK2	0.72727273	3.32467532	4.57142857
CCL28	0.68041237	3.10638298	4.56544165
CAMK2N2	0.28	1.27272727	4.54545455
MAFB	1	4.51219512	4.51219512
SAMD9L	7.46146146	33.5183033	4.4921901
DCHS1	0.76623377	3.44055944	4.49022164
EXOC3L1	1.28205128	5.74766355	4.48317757
NEK5	0.32467532	1.45454545	4.48
ADAMTS14	0.5	2.234375	4.46875
SEC14L5	0.25862069	1.15384615	4.46153846
DUOX2	1.53333333	6.81818182	4.44664032
SLC24A3	0.5	2.22222222	4.44444444
NCF2	1.23735409	5.49593496	4.44168328
NT5C3A	1.78731068	7.93523456	4.43976228
NEFH	0.32051282	1.41666667	4.42
TMEM232	0.20408163	0.89855072	4.40289855
LMO2	7.44827586	32.7875	4.40202546
NR2E1	0.43103448	1.89473684	4.39578947
ITK	0.34482759	1.51219512	4.38536585
KIF6	0.64102564	2.80487805	4.37560976
USP18	3.65333972	15.9776942	4.37344879
NOTCH4	0.51470588	2.23076923	4.33406593
TEKT2	1	4.33333333	4.33333333
KNG1	2	8.55555556	4.27777778
MS4A15	0.52631579	2.25	4.275
BEND4	0.51724138	2.2	4.25333333
INHBA	0.72727273	3.08219178	4.2380137
SCTR	0.5	2.11111111	4.22222222
MYO18B	0.78947368	3.33333333	4.22222222
BRINP2	0.20408163	0.86111111	4.21944444
GABRG3	0.33333333	1.40625	4.21875
GBP3	1.9017301	7.97688974	4.19454355
WARS	1.07636669	4.50629916	4.18658361
IRF1	1.85857337	7.76018856	4.1753469
STON1-GTF2	0.42900302	1.78873239	4.16951002
ZNF385D	0.19480519	0.81081081	4.16216216
AC005837.2	0.17241379	0.71428571	4.14285714
STPG2	0.86206897	3.55555556	4.12444444
PEX5L	0.55747126	2.295	4.11680412
IL15RA	1.67311828	6.84499055	4.09115759
PSMB9	1.43437892	5.84024267	4.07161775
TMEM163	0.38317757	1.55675676	4.06275544
TFEC	0.55434783	2.25174825	4.06197724
C2CD4A	0.77966102	3.15979381	4.05277902
C4A	1.10837438	4.48773842	4.04893733
PIGR	1.16666667	4.69444444	4.02380952
ANKRD20A2	0.59146341	2.37654321	4.01807306
LEFTY1	0.20408163	0.81818182	4.00909091
CHRNG	0.57471264	0.14354067	0.24976077
SYPL2	1.53333333	0.38235294	0.24936061

DLGAP1	1.33793103	0.33333333	0.24914089
CATSPER3	1.22413793	0.30496454	0.24912596
SYNPO2L	1.07894737	0.26760563	0.24802473
TM6SF2	2.41237113	0.59493671	0.24661906
ANKRD34A	1.35294118	0.33333333	0.24637681
MYOM1	1.73913043	0.42538976	0.24459911
DRAXIN	2	0.48888889	0.24444444
ARPP21	2.15789474	0.52631579	0.24390244
NTRK1	1	0.24324324	0.24324324
AC005702.2	0.79310345	0.18965517	0.23913043
PCDHAC1	1.60416667	0.38356164	0.23910336
NRG3	1	0.23684211	0.23684211
ZC3H11B	1.27941176	0.30246914	0.23641266
TG	1.7755102	0.41880342	0.23587779
CCDC36	1	0.23404255	0.23404255
PCBP3	1.87356322	0.43783784	0.23369259
ADGRB3	2.81034483	0.65517241	0.23312883
B3GNT6	0.66086957	0.15217391	0.23026316
PKHD1	1	0.23	0.23
ASPDH	1.5	0.34375	0.22916667
CFAP53	2	0.45794393	0.22897196
DCAF4L1	2.22916667	0.50961538	0.22861251
CT45A8	2.23376623	0.51	0.22831395
AC245748.1	1.94871795	0.44318182	0.22742225
TEX35	3	0.68085106	0.22695035
PAGR1	1.01371653	0.22914946	0.22604886
TFF1	6.1	1.37748344	0.22581696
FGF1	5.05263158	1.13953488	0.22553295
ASIP	2.48979592	0.56	0.22491803
C8G	1	0.2238806	0.2238806
DIRAS3	1.29878049	0.28947368	0.22288115
AC034102.2	5.27586207	1.17241379	0.22222222
AC008073.3	2.5	0.55	0.22
RBM44	1.05747126	0.23255814	0.21991911
CYP2C9	1	0.2195122	0.2195122
CSDC2	1.23780488	0.27142857	0.2192822
EFHC2	1	0.2183908	0.2183908
ASB5	4.6	1	0.2173913
HIGD1B	1.84482759	0.4	0.21682243
ZNF649	4	0.86486486	0.21621622
LRRC32	5.6	1.20979021	0.21603397
UGT1A10	2.19318182	0.47252747	0.21545294
C11orf16	3.68965517	0.78947368	0.2139695
PPP1R3E	1.12413793	0.23966942	0.21320286
PTPRC	4.05263158	0.86363636	0.21310508
MATN4	2.5	0.52941176	0.21176471
DLX2	4.65517241	0.96309963	0.20688807
FAM107A	4	0.81818182	0.20454545
ALOX5	2.20833333	0.45098039	0.20421754
FAM83C	3.05	0.62222222	0.20400729
SCX	2.3877551	0.484375	0.20285791
CFAP73	1.56410256	0.31666667	0.20245902
DNALI1	1.84482759	0.37209302	0.20169528
RNF225	2.44827586	0.49074074	0.2004434
CD5	1.5	0.3	0.2

FOXH1	6.04166667	1.20512821	0.1994695
MAPK13	2.13157895	0.42222222	0.19807956
NUTM1	1.11494253	0.2195122	0.19688207
ADCYAP1R1	5.1	1	0.19607843
NDUFC2-KCT	2.27147766	0.44405594	0.1954921
SCHIP1	2	0.3908046	0.1954023
A4GNT	1.12987013	0.21978022	0.19451813
CCDC154	2.65517241	0.51351351	0.19340119
TMEM190	4.3	0.82142857	0.1910299
LSMEM2	2.11688312	0.40384615	0.19077395
HRC	7.7	1.46153846	0.18981019
TMEM45B	4.31578947	0.81818182	0.18957871
ZNF571	1.5	0.27941176	0.18627451
KDR	1.6	0.29589041	0.18493151
MYOZ1	5.43333333	1	0.18404908
AC007375.2	1	0.18367347	0.18367347
GCOM1	1.36082474	0.24903475	0.18300281
CXorf58	1.5862069	0.28947368	0.18249428
KRT12	2.04123711	0.37209302	0.182288
NSG1	1.57894737	0.28571429	0.18095238
FGR	2.5	0.45	0.18
APOBEC3D	4	0.71428571	0.17857143
RDH16	4.35	0.77181208	0.17742806
SHISA3	1.43870968	0.25396825	0.17652502
MYO1H	0.74626866	0.13043478	0.17478261
SNAI2	4.06666667	0.70408163	0.17313483
PABPN1L	7.1	1.22222222	0.17214397
SMLR1	2.5	0.42857143	0.17142857
PRRX1	2.36666667	0.4	0.16901408
ELAVL3	3.51724138	0.58181818	0.16541889
BEST1	1.42962963	0.23451327	0.16403778
KCNN2	4.1	0.66956522	0.16330859
FAT2	5.1	0.82978723	0.16270338
AZU1	0.64125561	0.10344828	0.16132144
AC009163.2	2	0.32258065	0.16129032
AC004754.1	1.92073171	0.30769231	0.16019536
HMCN2	4.6	0.73170732	0.15906681
ASB14	2.5	0.39285714	0.15714286
ALS2CR12	6.1	0.95555556	0.15664845
EVX1	5	0.77659574	0.15531915
MEOX2	2.10344828	0.32142857	0.1528103
LMNTD1	1.03448276	0.15714286	0.15190476
KRT23	7.78947368	1.18085106	0.15159574
AC004080.3	8.1	1.22222222	0.15089163
ZNF713	2.61538462	0.38848921	0.14853999
SH2D7	1.13471503	0.16806723	0.14811404
CD72	1.64367816	0.2406015	0.14637994
SLC38A4	1.63157895	0.23684211	0.14516129
AC010463.1	0.76923077	0.11111111	0.14444444
C2CD6	3.1	0.44186047	0.14253563
DCDC2B	0.68965517	0.09782609	0.14184783
TRPV6	1.4137931	0.2	0.14146341
MYB	3.27941176	0.45238095	0.13794576
GOLGA8O	3.16883117	0.43442623	0.13709352
C17orf105	4.6	0.625	0.13586957

PTPN7	4.05	0.55	0.13580247
NPW	1.17241379	0.15862069	0.13529412
AMHR2	1.31034483	0.17699115	0.13507219
SHBG	2.28358209	0.3046875	0.13342525
B3GAT2	5.1	0.64957265	0.12736719
FAM46C	11.1	1.34754098	0.12140009
SSTR5	2.10526316	0.25388601	0.12059585
PROZ	1.75	0.20930233	0.11960133
ATOH7	3	0.35185185	0.11728395
MSLN	4.6	0.53125	0.11548913
BNIPL	2.8	0.32183908	0.11494253
RAPSN	1.5	0.16981132	0.11320755
IGSF23	6.6	0.74418605	0.11275546
LRRC25	2	0.2244898	0.1122449
LAMA1	9.1	1.00819672	0.11079085
SMPX	3.55	0.39285714	0.11066398
ILDR2	5.89473684	0.64556962	0.10951627
PABPC5	2.06122449	0.2244898	0.10891089
MISP3	1.50119904	0.16097561	0.10723136
SLC16A11	2.69117647	0.28571429	0.10616706
AC002985.1	1.93103448	0.2037037	0.10548942
KRTAP2-3	1.37931034	0.14516129	0.10524194
PRR18	1.82051282	0.19148936	0.1051843
FNDC1	2	0.20930233	0.10465116
CXCR3	3.1	0.32142857	0.10368664
TMEM215	4.6	0.46938776	0.10204082
KCNC2	7.6	0.75675676	0.09957326
MUSK	4	0.39285714	0.09821429
DYNC1I1	2	0.19148936	0.09574468
SERPINA3	6.35	0.60443038	0.09518589
PRR16	2.06349206	0.1958042	0.09488973
CELF3	4.6	0.42857143	0.0931677
PRSS53	2.5	0.2195122	0.08780488
OTP	4.6	0.3943662	0.08573178
CDHR1	7.21052632	0.61347518	0.0850805
KRTCAP3	4.1	0.34615385	0.08442777
CDC42EP5	3.3	0.25555556	0.07744108
PREX2	2.8	0.21568627	0.07703081
FAM49A	4.20689655	0.31967213	0.07598764
TERB1	3.3	0.24468085	0.07414571
MROH2A	4.36666667	0.32142857	0.0736096
GFRA2	0.85849057	0.06206897	0.07230011
AC018512.1	8.1	0.55555556	0.06858711
SCN1A	3	0.2	0.06666667
KCNJ3	1.09859155	0.07258065	0.066067
TBC1D3C	9.2	0.60526316	0.06578947
PLGLB2	4.6	0.3	0.06521739
ENPP5	9.6	0.62162162	0.06475225
IGSF5	2.5	0.15517241	0.06206897
ATG9B	2.15789474	0.12643678	0.05859265
HTRA3	2.5	0.14285714	0.05714286
TCTEX1D4	4.78947368	0.23469388	0.04900202
NMU	4.35	0.20930233	0.04811548
SLC11A1	4.6	0.2183908	0.04747626
DUSP2	4.6	0.19417476	0.0422119

FOXD4L6	8.7	0.359375	0.04130747
TTLL13P	7.15	0.29457364	0.04119911
ESRRB	6.6	0.24324324	0.03685504
MYOZ3	5.6	0.2	0.03571429
C3orf35	16.7	0.59223301	0.03546305
NRXN2	6.1	0.21111111	0.03460838
ANKRD61	4.6	0.14285714	0.0310559
TSPAN11	3.05	0.08737864	0.02864873
