# Supplementary material for: The R2TP complex regulates paramyxovirus RNA synthesis
Source: PLoS Pathog. 2019 May 23;15(5):e1007749. doi: 10.1371/journal.ppat.1007749 (PMC6532945; doi:10.1371/journal.ppat.1007749)
Supplement: S4 Table — (PDF) [file ppat.1007749.s010.pdf]

S4 Table. List of differentially expressed genes between mock-infected RPAP3-knockdown and control A549 cells

Gene name	Fold Change	FDR
SLCO2B1	#DIV/0!	0
PAPPA2	#DIV/0!	3.34576E-11
CALB2	#DIV/0!	3.22966E-12
FGFBP1	#DIV/0!	0
STMN3	#DIV/0!	0
NPY4R2	#DIV/0!	5.17094E-11
CPA4	#DIV/0!	0
CREB3L1	#DIV/0!	3.40983E-14
ECM1	34.3	0
EVA1C	32.9	0
CPLX1	25.58419244	1.93749E-13
SLC16A6	25.4	0
ITGAX	20.6	0
ASB4	17.38613861	0
COL17A1	16.3	0
ALDH3A1	15.2	0
ITGA10	14.7	5.70666E-14
EBI3	14.56666667	0
DHRS2	14.5	0
UNC13A	13.74	2.68704E-12
CYP4F12	13.1	1.14612E-11
TGM2	12.4	0
SERPINE2	12.4	0
ANKRD22	12.05	0
VDR	12.05	0
TNFRSF9	12.03181818	0
DOC2B	10.86666667	0
NNMT	10.43093923	0
SLAMF7	10.13333333	0
TRPC6	9.728571429	5.77275E-11
PLXNA2	9.6	0
TNC	9.545454545	0
TMOD1	9.23125	0
CHST6	8.706060606	6.18305E-12
CNIH3	8.407272727	1.80419E-11
SYT13	8.3	1.34425E-12
SPHK1	8.099099099	0
GPNMB	8.090224277	0
NPTX1	8.05	2.48799E-12
ANK1	7.7	1.8787E-11
SLC6A17	7.688888889	0
TSPAN8	7.666666667	0
NT5E	7.6	0
LAMB3	7.55	0
ABCA8	7.400374181	1.18225E-12
MPZL3	7.366666667	0
BEND6	7.3	1.45138E-11
FOXQ1	7.245454545	0
PHLDA2	7.22420078	0
THBD	7.09102402	0
RRAD	7.066666667	0
LYNX1	7.066666667	8.32745E-14
C1orf116	6.766666667	0
TSPAN7	6.725	3.61743E-15

CTSL	6.277272727	0
MALL	6.2	0
HRH1	6.109677419	0
CDKN1A	6.0625	0
ANXA13	6.039145907	0
ITGA3	5.975	0
SLC51B	5.95	5.38911E-14
PLA1A	5.88	0
SFRP1	5.852459016	0
PLSCR4	5.84	0
AKR1C1	5.757575758	0
GABRA5	5.756756757	0
SLC1A1	5.733333333	2.88074E-11
LHFPL6	5.727272727	2.03298E-13
TGFA	5.725274725	0
PRDM13	5.636363636	6.28986E-11
ANGPT1	5.584158416	2.09468E-13
SLC2A12	5.418604651	0
NAMPT	5.305882353	0
TDO2	5.279545455	2.26206E-12
ANPEP	5.220689655	0
TNFSF15	5.163593698	0
MT2A	5.153258888	0
CHPF	5.070240296	0
SCG2	5.064220183	0
KCNN4	5.06	0
ABLIM3	4.985074627	0
TNFRSF10D	4.969767442	0
PCOLCE2	4.950920245	0
SERPINB8	4.911764706	0
TCN2	4.889502762	0
PLCD4	4.866666667	5.62702E-12
HPCAL1	4.85	0
GATA2	4.801801802	3.15021E-12
FAS	4.775	0
FA2H	4.691275168	0
CDR1	4.677809205	0
ACOT1	4.672268908	1.74883E-14
FLG	4.588679245	9.91544E-13
PLA2G4C	4.560747664	0
AKR1B10	4.554455446	0
STAMBPL1	4.515555556	0
PLA2G16	4.504545455	0
FGFBP3	4.477958237	2.37665E-13
GFOD1	4.471949104	0
SLC9A2	4.468468468	0
ARSG	4.46	0
OAF	4.450723639	0
SDK2	4.430274754	8.19287E-11
ID2	4.388068182	0
SQSTM1	4.361445783	0
SH2D5	4.338461538	0
PDK4	4.306930693	0
EDEM3	4.3	0
FAM102A	4.294491525	0
SERPINB7	4.279069767	3.65709E-11

GPX2	4.266903915	9.30718E-14
SMOX	4.257731959	0
SMPD1	4.24	0
AMPD3	4.203682394	0
PANX2	4.173913043	0
FYCO1	4.17087846	0
PLK3	4.166666667	0
ADGRF4	4.1546875	1.18586E-13
SFR1	4.148247978	0
HTRA1	4.137203166	0
AKR1B1	4.104347826	0
WSCD1	4.038674033	0
TTPAL	4.02244898	0
TMCO1	3.961538462	0
PLXNB3	3.93129771	7.52488E-12
ANTXR1	3.917142857	0
KCNF1	3.915742794	0
SRGN	3.899115044	0
RGS2	3.885714286	0
CYB5B	3.88125	0
CCNG1	3.868217054	0
APOC1	3.862253156	1.93749E-13
SLIT3	3.803571429	0
SPX	3.802469136	0
CEACAM1	3.8	4.1857E-13
SLC35D1	3.769565217	0
GPAT3	3.724206349	0
CXCL2	3.707070707	6.68539E-14
MYEOV	3.704255319	0
ANK2	3.688468158	0
ADI1	3.688235294	0
SLC16A4	3.686609687	0
RPS27L	3.683168317	0
EREG	3.647826087	0
DUSP5	3.632	0
BTBD11	3.624137931	0
ETV1	3.62	8.94892E-12
NFE2L3	3.601421189	0
ALDH2	3.592705167	0
LYSMD2	3.582781457	0
HLA-B	3.580826109	0
KITLG	3.557142857	0
OSGIN1	3.534050179	0
SULF2	3.511450382	0
SRPX2	3.5	0
RSPH3	3.495682211	1.4063E-14
ITGA2	3.488461538	0
ETFB	3.488235294	0
NABP1	3.485714286	1.06497E-14
PODXL2	3.466	4.06555E-14
CXCL5	3.442424242	0
MR1	3.435643564	0
C16orf52	3.402083333	0
RAB3B	3.399071926	0
CDK6	3.3875	0
NAV3	3.380952381	0

L3HYPDH	3.365418895	1.39159E-10
CD22	3.362308219	1.56173E-10
BCAN	3.360313364	1.89379E-10
DHRS9	3.358333333	2.13742E-10
FIBCD1	3.354340071	2.3113E-10
GJB2	3.353787879	2.36474E-10
CORO2B	3.338108883	2.92916E-10
PLXND1	3.325	2.95251E-10
CYP4F11	3.324691358	3.26689E-10
SNCG	3.293103448	5.02878E-10
LRP1B	3.277945619	8.87448E-10
SUSD2	3.273766226	9.20106E-10
GSAP	3.272	1.00675E-09
IFI44	3.26309795	1.03071E-09
CHST2	3.21	1.03866E-09
LAMA4	3.195706028	1.05489E-09
KCNJ6	3.193277311	1.17921E-09
COPZ2	3.191810345	1.40869E-09
TMEM27	3.191301789	1.4097E-09
DGKA	3.115277778	1.4914E-09
F2RL2	3.103370787	2.53103E-09
DIRAS1	3.100775194	4.13333E-09
AK5	3.081578947	5.38682E-09
AKR1B15	3.066666667	5.63469E-09
KRT15	3.066469909	9.23606E-09
PPT2	3.047619048	1.0056E-08
KIAA1324	3.044444444	1.05849E-08
SLCO5A1	3.036697248	1.22458E-08
COL4A6	3.02378774	1.43539E-08
RIMBP3	3.02130642	1.71526E-08
NIPAL4	3.019248396	1.75129E-08
DNM3	3.013986014	1.99963E-08
KIAA1644	3.013289037	2.1281E-08
KCNMA1	3.007237636	2.1281E-08
SNAP25	3	2.49895E-08
CD82	2.990707087	2.53339E-08
CRLF2	2.968571429	3.51536E-08
SUSD5	2.964407459	3.63181E-08
TMEM171	2.959574468	3.99578E-08
GJA3	2.954857704	4.13029E-08
TBXAS1	2.95	5.15763E-08
SMAGP	2.95	5.88613E-08
HHIPL2	2.944655042	6.52111E-08
NPL	2.93129771	6.97281E-08
DTX1	2.92150638	9.4279E-08
CDH10	2.92051756	9.77725E-08
KCNK3	2.915966387	1.24766E-07
KLRG1	2.913333333	1.37246E-07
CBLN3	2.906060606	1.59199E-07
CCK	2.905982906	1.70358E-07
SHC4	2.905335628	1.70358E-07
GCOM1	2.895679481	2.66036E-07
AMDHD1	2.87654321	2.91138E-07
UGT1A6	2.874850657	3.07811E-07
DGAT2	2.874551971	3.55412E-07
CYGB	2.857391304	5.23224E-07

AC068234.1	2.855326269	5.69112E-07
ACOX2	2.846361186	6.17159E-07
ST6GAL2	2.845238095	6.21139E-07
CASKIN1	2.8375	7.46684E-07
TSPAN1	2.827945777	7.94177E-07
NIPAL1	2.818638574	7.98305E-07
KCNC3	2.81641881	9.25646E-07
SORCS2	2.815555556	9.87578E-07
ALOXE3	2.815384615	1.03607E-06
INPP1	2.808219178	1.1024E-06
PTCHD4	2.799653979	1.14392E-06
TRPM8	2.795306802	1.15443E-06
ARC	2.794828744	1.49804E-06
AQP11	2.794621027	1.57088E-06
SMOC1	2.793068298	1.75886E-06
MX1	2.790988736	1.9177E-06
FKBP7	2.788157895	1.98671E-06
CHGA	2.785310734	2.13177E-06
PKHD1L1	2.780952381	2.22697E-06
TRPA1	2.775147929	2.46908E-06
IFI16	2.767075306	2.52185E-06
NECTIN4	2.747058824	3.77191E-06
ADM2	2.735294118	4.39611E-06
MFSD2A	2.73414305	4.82123E-06
RCBTB2	2.7	5.54563E-06
MPZ	2.686842105	6.1335E-06
BST2	2.67826087	6.37234E-06
DCLK1	2.672785166	6.58414E-06
FGF13	2.672176309	6.92009E-06
NTNG2	2.669634026	6.97578E-06
IL7R	2.664530275	8.14028E-06
ENPEP	2.663636364	8.70118E-06
SLC12A8	2.661904762	1.08104E-05
KCNQ5	2.647619048	1.2138E-05
CDHR1	2.639054233	1.27414E-05
AIF1L	2.636658541	1.31336E-05
UNC5A	2.614840989	1.40821E-05
STC1	2.612223393	1.45908E-05
FOXD3	2.602853746	1.6716E-05
IGFBP7	2.599033816	1.75256E-05
GABRA2	2.596	1.92995E-05
BATF2	2.591666667	1.93634E-05
GALNT5	2.587525151	2.51221E-05
SYT9	2.5875	2.73042E-05
COL13A1	2.586956522	2.95606E-05
S100P	2.585023401	3.12519E-05
RNF182	2.582551027	3.59117E-05
PLEKHH1	2.572605561	3.71538E-05
SYK	2.557570263	3.75108E-05
AC083841.5	2.547334924	3.75786E-05
SLC9A9	2.543244507	3.94535E-05
RMDN2	2.541519435	4.33328E-05
IFI6	2.522580645	4.5561E-05
FCGBP	2.518378888	5.14074E-05
SWSAP1	2.518109269	5.57753E-05
C4B	2.506936416	6.03491E-05

SOX18	2.504250797	6.15286E-05
RBFOX3	2.500869565	6.20672E-05
CMPK2	2.495588235	6.74338E-05
SLCO1A2	2.49132948	6.902E-05
L1CAM	2.474006116	7.91797E-05
DLL1	2.466631215	8.94163E-05
BHLHE41	2.459177848	9.84958E-05
SELL	2.455248094	0.000100113
UGT1A1	2.44235083	0.000110678
INPP5D	2.438082161	0.000125907
PADI3	2.433687943	0.000127682
TM4SF19	2.430476028	0.00013156
AGTR1	2.429151382	0.000145225
LAMC3	2.425581395	0.00015513
IL33	2.405281422	0.000169336
GIMAP2	2.404290354	0.000198693
AP3B2	2.403740374	0.000203807
FAM187A	2.399312272	0.000209613
GRID1	2.388548057	0.000211307
PRODH	2.388372093	0.000227138
SLC44A5	2.386792453	0.000229909
SOX8	2.386292835	0.000231225
CGNL1	2.384719405	0.000231794
C15orf59	2.381967213	0.00023291
CBFA2T3	2.380769231	0.000275881
HOXB8	2.38	0.000319078
VGF	2.373653111	0.000346456
AQP4	2.373040752	0.000346859
LGI2	2.363050483	0.000355237
SH3TC2	2.3625	0.000365287
ADGRD1	2.358462676	0.000370631
TGIF2-C20orf24	2.355172414	0.000418277
PLAG1	2.347442681	0.000432514
GON7	2.343623071	0.000445233
DRAXIN	2.342857143	0.000457648
MAP1LC3B2	2.340740741	0.000460427
CSPG5	2.340438129	0.000462965
SLC6A14	2.33882888	0.000466879
AQP3	2.338246907	0.000476827
CACNA1A	2.335203257	0.000493839
KIF1A	2.331618067	0.000496178
TTLL11	2.32967033	0.000578249
CDNF	2.31582319	0.000596929
CPLX2	2.310088805	0.000598447
RPGRIP1	2.307620237	0.000606218
IQGAP2	2.306715064	0.000614845
AOX1	2.298065984	0.000624583
HES7	2.284936479	0.000655668
TAT	2.280672269	0.000697721
MMP1	2.271386431	0.000715252
FSTL4	2.253493891	0.000745238
MAF	2.252173913	0.00077417
TMEM251	2.252083333	0.000817065
DNAH17	2.248837209	0.000831281
APLN	2.245614035	0.000836042
TMEM45A	2.244456463	0.000855985

MAT1A	2.241860465	0.000872421
ADAM32	2.231754161	0.000889307
RGS16	2.230237504	0.000911582
ZNF423	2.227784731	0.000934936
SLC52A1	2.226123596	0.000944257
CXCL3	2.224242424	0.000994932
SPNS2	2.218915614	0.001057559
AL121722.1	2.216666667	0.001076227
DNAH3	2.215081731	0.001132015
GPR156	2.213286713	0.001133079
KAZALD1	2.212389381	0.001141528
AD000671.1	2.211363636	0.0011857
FHDC1	2.209814703	0.001191548
ITGB3	2.208088235	0.001221601
MARCH4	2.207408486	0.001228508
NKX3-2	2.206775701	0.001299336
HSPA6	2.203164681	0.001315748
TFF1	2.198541329	0.001336091
SNAI2	2.192095271	0.001389773
TMSB15B_1	2.189443921	0.001397139
SIDT1	2.186847195	0.001430598
ELMOD1	2.185058259	0.001477276
ARG2	2.185020549	0.001493735
CCL26	2.184615385	0.001705783
PAX2	2.179579466	0.001911264
LHX6	2.177955021	0.001928954
FAM43A	2.174456768	0.001963473
CES1	2.174358974	0.002026487
TNS1	2.171052632	0.002322204
ZNF571	2.169630643	0.002471417
CIDEB	2.168732061	0.002535674
DUSP13	2.165306122	0.002658884
CDYL2	2.163265306	0.002684183
MDK	2.161956522	0.003031652
CHST1	2.157142857	0.003145561
FAM46C	2.156488787	0.003198412
PRUNE2	2.15624786	0.00324872
SCN3B	2.155820776	0.003295328
SPOCD1	2.149090909	0.003417231
C8G	2.147986511	0.003451146
CYP39A1	2.136355158	0.003470049
PCDHGB1	2.134125637	0.00365971
PCDH1	2.128808201	0.003766099
PRSS3	2.127108186	0.003910127
R3HDML	2.123314066	0.004046614
CST1	2.119047619	0.004090115
CDH7	2.117610063	0.004090115
KCNB1	2.114942529	0.004090115
FCRLB	2.108593012	0.004130858
RHEBL1	2.107944732	0.004299141
TCHH	2.105405405	0.00432591
RET	2.105263158	0.004379829
LIPM	2.10505474	0.004688549
UNC5B	2.103701826	0.00479186
MAGEA1	2.103666245	0.004828623
C3orf80	2.097930182	0.004828623

SEPT4	2.095698925	0.004941374
PPM1E	2.094247644	0.005044683
RGS22	2.092307692	0.005153316
AC007906.2	2.0907173	0.005177077
DLX2	2.08556701	0.00520467
TM4SF19-TCTEX1D2	2.084054388	0.005276563
A1CF	2.08259587	0.005715953
AL591806.3	2.080884018	0.006372942
LAMA1	2.070434783	0.006476624
LRRC32	2.068799892	0.006534612
ASB2	2.068745571	0.006664443
KCNQ3	2.067321178	0.006884121
ALDOC	2.062992126	0.007032581
PSORS1C1	2.057660925	0.007320826
VPREB3	2.052578362	0.007394972
TUBAL3	2.045352042	0.007526387
CASP1	2.041176914	0.007557642
BCL2A1	2.040308534	0.007690449
OASL	2.032451923	0.007986497
BCL11B	2.031685678	0.008355858
MYB	2.026744186	0.008530259
SLC16A11	2.025490196	0.008540089
RGMA	2.023188406	0.009052943
AOAH	2.022244692	0.009070765
KCNN2	2.018171806	0.009070765
C9orf43	2.016735387	0.009284084
C10orf143	2.014113749	0.009355846
CEMIP	2.008333333	0.009798682
TRIB2	2.006874742	0.009813199
CCDC38	2.004081633	0.009821917
HRNR	2.001187648	0.009935362
ACPP	2.000647878	0.009992147
MZT2B	-2.011209054	1.54274E-07
HABP2	-2.012048193	0.000398269
HOOK1	-2.012796504	2.17324E-05
HES4	-2.013722127	1.04606E-06
CCDC184	-2.016756433	1.6392E-20
ASL	-2.019832525	2.41079E-08
MYO1B	-2.021122585	0
SOCS2	-2.025177026	1.28611E-30
COMMD3-BMI1	-2.025287356	0.000800737
KCND2	-2.025991792	2.33572E-06
MYLK	-2.026233223	1.2662E-179
SELENOP	-2.02753978	2.58596E-07
ECHDC3	-2.034458994	2.1053E-09
MPP2	-2.047381546	9.74628E-24
UCN2	-2.04954955	0.004668075
PLD2	-2.049736247	7.82905E-43
ELOVL2	-2.049844237	2.18776E-55
FBXO43	-2.049910873	0.00699036
HIST1H4I	-2.050185109	7.47468E-39
HIST1H2BI	-2.053643725	1.90903E-13
PAQR6	-2.054224464	0.000172908
ERBB2	-2.054869684	1.80286E-60
PCDHB5	-2.061078622	2.05039E-06
SNX4	-2.061952191	2.8385E-34

ZFP36L2	-2.064123755	5.22716E-13
SEMA3D	-2.065029861	1.10436E-05
PRSS23	-2.067543476	1.89232E-42
NYAP1	-2.068322981	0.000103246
ITGAV	-2.078640868	1.7244E-175
DNAH5	-2.084420881	1.51452E-16
MXD3	-2.091577162	8.38497E-07
MMP24OS	-2.092892024	3.71707E-25
CX3CL1	-2.093244529	5.34664E-07
AVPR1A	-2.104210526	5.12561E-09
DSCAML1	-2.106358382	0.001278935
B4GALT5	-2.113270637	8.2938E-123
CRISPLD2	-2.114311431	1.74715E-10
C8orf88	-2.117355372	8.26093E-05
RASGRP3	-2.11778563	0.000223324
TCF7L1	-2.12588513	2.40332E-10
HS3ST1	-2.126602564	1.80227E-13
INCA1	-2.126696833	0.009673888
SYNGR3	-2.127024723	1.35198E-21
CEP68	-2.128165771	0.000245743
SBK1	-2.131953428	0.009676714
TMEM130	-2.133247089	1.54275E-07
ZNF25	-2.133671743	7.61456E-08
SRSF12	-2.133757962	2.45291E-09
UBE2B	-2.13923605	8.01583E-13
HECA	-2.139830508	5.35461E-07
GNG2	-2.140258497	1.21396E-19
UGCG	-2.141735095	8.66575E-76
RASGRP1	-2.151202749	9.91123E-06
MSI1	-2.151515152	1.39061E-06
AC099489.1	-2.152416357	0.002692771
FFAR4	-2.160493827	0.000944488
DZIP1	-2.160495341	4.44655E-77
NECAB1	-2.164009112	7.64753E-05
ZNF704	-2.164375716	7.06193E-07
AMN1	-2.166088632	1.60636E-19
SERPINA6	-2.171270718	0.000367045
FAM126B	-2.171417415	1.23516E-13
SPON2	-2.175475687	2.76629E-05
LLGL2	-2.175653595	3.74317E-38
SRGAP1	-2.17943609	1.3031E-258
GRB7	-2.184047351	1.67615E-09
KRT80	-2.187193974	1.5109E-260
SPTB	-2.196988708	1.41452E-13
FAM71E1	-2.197771588	0.004701116
SPC24	-2.198106592	1.67566E-23
PGM2L1	-2.200040741	2.8526E-197
HMGCLL1	-2.203742204	0.000597799
SORL1	-2.204868795	1.18309E-34
C12orf75	-2.221081837	0
SEC61A2	-2.222672749	8.47479E-34
COL1A1	-2.222954489	1.74736E-33
PPL	-2.223174603	1.83748E-13
VASP	-2.224968279	7.38483E-96
CCDC150	-2.229122056	1.7476E-05
SLC6A16	-2.230072464	0.009045274

AHRR	-2.230273752	3.78333E-06
LCN2	-2.233449477	0.001830534
TM4SF4	-2.235384419	2.81661E-10
CD37	-2.236096537	1.07532E-08
KLC3	-2.236842105	0.003703046
ERFE	-2.241333333	1.50043E-08
HDAC5	-2.244617669	7.94697E-31
IGSF11	-2.24556213	0.000993093
WEE1	-2.25209477	2.1421E-32
C5AR1	-2.256774194	6.98509E-09
SLITRK3	-2.258196721	0.002506996
OLFML2A	-2.258231627	7.68906E-26
SLCO4C1	-2.259481038	0.00832089
SP4	-2.260940562	2.08927E-24
KIF5C	-2.262086514	0.001670478
FAM167A	-2.268628382	1.22585E-19
SERPINF1	-2.275142315	1.54915E-06
FNBP1L	-2.275637154	4.5341E-35
MUC13	-2.283640643	7.18264E-20
AATK	-2.300291545	0.000333502
IL17RD	-2.30878553	3.64559E-27
ENPP1	-2.320227694	4.30234E-21
SLC2A3	-2.326308747	1.24616E-09
LCMT1	-2.327172414	1.35214E-15
CYR61	-2.331376083	1.15791E-68
RAB26	-2.337142857	7.83363E-46
PRODH2	-2.341463415	0.003674626
EPCAM	-2.346118721	3.3501E-14
LPP	-2.347894606	0
KIF21B	-2.352179837	2.27372E-06
VWA7	-2.352708058	3.17374E-14
GRIN3A	-2.36453202	0.007108778
ANXA3	-2.366765972	2.75911E-75
USP43	-2.371263765	3.59103E-17
FTH1	-2.37223741	1.8146E-179
DOT1L	-2.382898629	6.40491E-33
SLC2A13	-2.38447176	2.64543E-28
KLB	-2.385938669	3.55514E-34
CTSK	-2.388888889	0.005101771
DAPK1	-2.393913779	7.05398E-72
CACNG7	-2.396073903	1.93721E-22
DNAJC25-GNG10	-2.400203666	0.000247769
FES	-2.405345212	1.34057E-06
RAP1GAP2	-2.406116285	2.07415E-27
DNAJC15	-2.409482759	0.000998736
EDIL3	-2.411461022	1.7763E-25
TGFB2	-2.414446928	0
MYH10	-2.415478716	6.1545E-262
SIM2	-2.422861711	1.06936E-31
GRHL3	-2.4238653	2.03839E-11
MCRIP1	-2.43133964	2.17693E-38
NMUR2	-2.44152431	0.000504464
CTSV	-2.441651706	0.000264292
ALDH1A3	-2.447550201	6.41017E-71
MALRD1	-2.461538462	0.002139838
SPARC	-2.472765181	2.01189E-45

SUCNR1	-2.501686025	2.05325E-14
NPY1R	-2.511169514	1.12578E-22
CDS1	-2.512486428	6.19231E-21
ADRA1D	-2.513370474	6.54553E-52
AKAP12	-2.514022191	0
C2orf82	-2.524475524	0.008390144
CCDC80	-2.530431649	8.32102E-66
RAPGEFL1	-2.531400966	2.26826E-07
ERLIN1	-2.537199819	2.80317E-71
NOVA2	-2.537900875	2.70736E-08
ADSSL1	-2.540473225	8.18496E-08
CAPN12	-2.541747573	2.61777E-08
CLDN7	-2.543478261	1.88226E-07
OCLN	-2.545096554	6.611E-196
COL12A1	-2.546893181	3.91391E-39
USP44	-2.548245614	0.000370631
INHBB	-2.557137641	3.95511E-27
CPN1	-2.563176895	0.004055561
CHAF1B	-2.570122822	5.19938E-39
TNFRSF21	-2.586790787	0
PRICKLE1	-2.599160546	4.84979E-11
MPP7	-2.623574144	9.17115E-05
SSC5D	-2.641509434	0.007972389
SLC3A1	-2.646440129	7.66053E-16
TGM3	-2.665467626	0.000586801
NCALD	-2.667054038	8.75839E-24
NPY5R	-2.67539267	0.000866442
DMBX1	-2.686746988	1.236E-10
LCLAT1	-2.702888752	2.51434E-56
EGR1	-2.710332103	8.01E-11
NRTN	-2.714285714	1.21785E-05
RND1	-2.715155616	1.72516E-16
LIMD2	-2.72027972	4.16015E-14
FGB	-2.733123666	7.14374E-07
BRINP1	-2.755813953	0.001221601
B3GNT5	-2.763001975	1.01384E-06
SDR42E1	-2.767857143	0.009242754
RFX6	-2.777777778	0.001452861
DUSP8	-2.80332829	5.28024E-24
TNFRSF11B	-2.808988764	9.61592E-06
VIL1	-2.817427386	0.00307696
PCDH17	-2.820512821	0.000194421
DOCK2	-2.82208589	0.000824431
TNXB	-2.836512262	9.47186E-08
C1orf228	-2.843283582	0.0024086
TRIM6-TRIM34	-2.845794393	0.003113786
NTS	-2.853303471	1.75256E-05
SHH	-2.873271889	7.01915E-26
CNFN	-2.909178744	4.52165E-19
CREB5	-2.92605042	6.36699E-13
GK	-2.932135729	5.15696E-06
SBSPON	-2.933962264	0.005847826
FAM133A	-2.939297125	1.35332E-06
SFRP4	-2.95727198	1.61502E-15
ARHGAP19	-2.96460177	0.000199802
PQLC3	-2.977943062	2.06946E-85

MYOCD	-2.978494624	0.000619547
ZNF608	-2.991641663	2.92758E-32
MGAT4C	-3.013377926	5.42927E-13
BNC1	-3.059701493	0.000793154
DCDC1	-3.074935401	5.99931E-06
CORO2A	-3.076286221	3.62635E-11
REEP2	-3.079796265	4.50935E-11
CRYBG2	-3.097087379	0.003504709
CNTD1	-3.103092784	0.004695894
KRT4	-3.103343465	3.42634E-08
KRTAP3-1	-3.137254902	3.11356E-09
DACT1	-3.14516129	0.000907638
SCNN1A	-3.241598306	1.52964E-24
COL26A1	-3.264864865	1.88393E-16
TRIM31	-3.270501836	3.31098E-07
KCNJ16	-3.312637882	4.36441E-35
MUC16	-3.31838565	1.01979E-06
IL11	-3.324574961	6.83691E-10
TGFBR1	-3.388378124	5.193E-242
ADAM28	-3.400222965	3.57524E-26
DENND2A	-3.439490446	0.000797455
PELI1	-3.456028369	4.99104E-35
SLC4A8	-3.459770115	2.19551E-46
NPR1	-3.493196334	7.4842E-110
PDIA6	-3.503539373	1.1277E-250
ATF3	-3.506056528	1.72652E-42
UNC13D	-3.519000797	1.0786E-201
C1QL1	-3.531374106	7.50996E-16
TMEM52B	-3.546511628	5.084E-06
PDE5A	-3.607218684	6.86148E-22
LRRC36	-3.676470588	0.001306439
CTNND2	-3.679794521	1.14849E-16
SULT2B1	-3.699411765	9.95586E-24
VIPR1	-3.701174743	8.61431E-32
PIANP	-3.731343284	2.46448E-05
CGN	-3.738251041	3.02831E-25
DMKN	-3.813466788	2.28002E-29
MGAM	-3.823414634	1.32123E-43
KRT20	-3.87195122	3.69794E-35
CHML	-3.911591356	1.04946E-19
TLR5	-3.936651584	1.48961E-08
HCAR1	-4.057971014	0.002351691
YPEL1	-4.057971014	0.002396815
FBLN7	-4.117647059	0.007376412
PADI2	-4.210818308	1.3752E-115
SCG5	-4.246428571	3.12548E-10
UPK3B	-4.253708634	6.7489E-155
ESAM	-4.285100287	1.60774E-18
TNNC1	-4.347826087	6.4612E-05
LIPC	-4.366197183	0.000561411
GNG10	-4.480625	1.90725E-82
BMF	-4.484605087	1.77434E-06
SBK2	-4.487804878	8.96649E-07
BPIFB1	-4.625	2.2752E-05
FLRT3	-4.629152013	6.15714E-62
CALHM5	-4.664	7.10168E-20

ADRA2A	-4.976945245	7.78059E-05
SLC16A12	-4.983870968	0.000247992
FAT3	-5.33599574	2.5498E-122
DEFB1	-5.396103896	0.000121371
GREM1	-5.534937888	1.09594E-89
DRC7	-5.629032258	0.000444243
PDGFB	-5.791324736	2.60297E-61
COL20A1	-5.853658537	0.00085507
TP53TG3B	-5.862068966	0.0071014
TINAGL1	-6.024096386	3.41815E-07
UNC13C	-6.116504854	2.56368E-05
OLR1	-6.127208481	2.56092E-60
SYT11	-6.514285714	5.15637E-16
GNG7	-6.5625	0.003648197
FOXJ1	-6.65802269	1.01317E-21
B3GNT8	-7.142857143	0.005332513
IL32	-7.560240964	3.72632E-34
NID1	-8.272962484	1.92509E-22
NKAIN4	-8.460526316	1.43629E-25
SUSD4	-9.088461538	4.1923E-08
CDH5	-9.859649123	2.22897E-21
RPAP3	-19.7589658	0
