# Supplementary material for: The R2TP complex regulates paramyxovirus RNA synthesis
Source: PLoS Pathog. 2019 May 23;15(5):e1007749. doi: 10.1371/journal.ppat.1007749 (PMC6532945; doi:10.1371/journal.ppat.1007749)
Supplement: S5 Table — (PDF) [file ppat.1007749.s011.pdf]

S5 Table. List of differentially expressed genes between MuV-infected and unin

Gene name	Fold Change	FDR
CXCL10	#DIV/0!	0.001001468
CXCL11	#DIV/0!	0.000282499
IFNB1	#DIV/0!	6.22702E-06
IFNL3	#DIV/0!	2.8008E-06
IFNL2	#DIV/0!	4.79404E-07
IFNL1	#DIV/0!	6.5933E-12
CH25H	#DIV/0!	0
RSAD2	111.9	0
CCL5	106.177305	0
OAS2	67.8125	0
IFIT2	62.06451613	0
OASL	54.00900901	0
IFIT1	34.3875	0
HCAR2	32.3	1.6265E-05
GBP4	20.30508475	0
ISG15	17.91	0
APOL3	11.93333333	1.44614E-05
RARRES3	7.463687151	2.37889E-09
DHRS2	7.440677966	1.7245E-05
IFI44	6.689265537	0
HERC5	6.527620397	0
IFIH1	5.951320132	0
PLEKHA4	5.321782178	2.78642E-12
DDX60	4.59530262	0
PARP10	3.905405405	0.000221967
C21orf33	3.385416667	2.2112E-07
ISG20	3.30952381	0
TNFSF10	3.265895954	0.003943123
DDX58	3.132326821	0
FST	2.877068558	1.80612E-06
KLF4	2.553540082	0
SAMD9L	2.52166065	0.006356376
APOL1	2.433053469	6.08619E-09
APOL6	2.420465726	0
SEMA3D	2.313501144	0.000952131
IFI6	2.208938547	6.4873E-08

PMAIP1	2.13452728	0.000684272
RGPD6	2.107509881	5.03697E-09
ITPRIPL2	2.100801833	0.000369078
MXD1	2.045077106	3.48576E-12
IRF1	2.00270027	9.90993E-11
XAGE1A	-2.017817372	0.007766771
DNAJC25-GNG10	-2.331223629	0.003179512
MTRNR2L1	-2.940785247	0.002805364
OR2C3	-3.513368984	6.56033E-05
F8A3	-3.602316602	0.004420072
AC092143.1	-3.731117825	2.03773E-07
MTRNR2L8	-4.530296403	2.25946E-37

nfected RPAP3-knockdown A549/SeV-C cells
