# Supplementary material for: The R2TP complex regulates paramyxovirus RNA synthesis
Source: PLoS Pathog. 2019 May 23;15(5):e1007749. doi: 10.1371/journal.ppat.1007749 (PMC6532945; doi:10.1371/journal.ppat.1007749)
Supplement: S6 Table — (PDF) [file ppat.1007749.s012.pdf]

S6 Table. List of differentially expressed genes between MuV-infected and uninfected control A549/SeV-C cells

Gene name	Fold Change	FDR
CCL5	19.71428571	2.26224E-05
OASL	11.71493213	0
DHRS2	9.577464789	0
GDPGP1	4.082089552	0.004006418
ZNF419	3.330232558	0.000164463
TCTEX1D2	2.337236534	0.001174782
DDX60	2.068204614	0.001133946
