# Supplementary material for: The R2TP complex regulates paramyxovirus RNA synthesis
Source: PLoS Pathog. 2019 May 23;15(5):e1007749. doi: 10.1371/journal.ppat.1007749 (PMC6532945; doi:10.1371/journal.ppat.1007749)
Supplement: S7 Table — (PDF) [file ppat.1007749.s013.pdf]

S7 Table. List of differentially expressed genes between MuV-infected and uninfected RPAP3-knockdown A54[illegible]

Gene name	Fold Change	FDR
HCAR2	#DIV/0!	0.002111993
IFNL2	#DIV/0!	0.000690459
RAET1L	#DIV/0!	4.79598E-05
GBP5	#DIV/0!	4.03987E-05
SLC15A3	#DIV/0!	1.922E-06
CH25H	#DIV/0!	6.01467E-07
DHX58	#DIV/0!	2.05672E-13
RSAD2	#DIV/0!	0
XAF1	#DIV/0!	0
OAS2	782.1724138	0
IFIT1	760.9264706	0
IFIT3	579.0769231	0
GBP4	467.2222222	0
MX1	388.0381944	0
IFITM1	331.05	0
IFIT2	292.8823529	4.99279E-06
CMPK2	245.3697479	0
ISG15	138.627907	0
MX2	134.974359	0
BST2	133.45	0
OASL	133.1075697	0
CCL5	99.08974359	0
IFI44L	92.83673469	0
IFI44	79.49044586	0
IFI6	77.75324675	0
IFNL1	77.27272727	5.16028E-08
IFI27	71.37313433	0
APOL3	70.55555556	0.00040785
LAMP3	67.97222222	0
THEMIS2	65.14285714	0
PARP10	63.65142857	0
IFNL3	50	0.000324205
HELZ2	42.3089934	0
IRF7	41.13129496	0
HSH2D	40.42857143	0
TMEM140	39.5	1.87696E-10
LMO2	37.95	0
SMTNL1	36.63636364	2.49384E-08
BATF2	32.69463869	0
UBA7	31.925	0.000999198
TRIM22	28.89759036	0
IFIH1	28.08662026	0
DDX60	24.07330827	0
DDX58	22.23934018	0
SAMD9L	21.96226415	0
PLEKHA4	21.69142857	0
SAMD9	20.20087099	0
APOL6	20.16973913	0
HERC5	19.8265896	0

SP110	18.78721174	0
GBP1	18.35421687	0
EYS	17.66666667	0.005173862
PARP9	17.33298283	0
TNFSF10	15.91280654	0
OAS3	14.19165518	0
TRANK1	13.61950018	0
NLRC5	13.38442822	0
SERPING1	13.13333333	6.52113E-07
PARP12	12.93943384	0
OAS1	12.56922258	0
IFI35	12.50542942	0
CX3CL1	12.36842105	0.001146932
DTX3L	12.32791995	0
USP18	12.29432133	0
HERC6	11.52902574	0
LGALS9	11.39411765	2.40163E-08
AL669918.1	11.02597403	0
CD274	10.94148936	5.56006E-12
C5orf56	10.88135593	2.4308E-09
DDX60L	10.38222427	0
CYP2J2	10.38095238	0.002404059
PARP14	10.29590989	0
IRF9	9.221458047	4.70625E-14
DHRS2	9.103092784	4.35993E-11
ISG20	8.83655536	0
DAPP1	8.607142857	0.004832256
IFI16	8.517388652	0
PLSCR1	8.468250235	0
IFIT5	7.353072004	0
PSMB9	7.254728878	3.52365E-11
STAT2	7.058750311	0
TAP1	6.892113565	0
C19orf66	6.87191461	0
PPM1K	6.861867704	0
RASGRP3	6.598890943	0
SAMHD1	6.582033687	0
UBE2L6	6.576752269	0
PML	6.471468425	0
TRIM21	6.409494232	0
IRF1	6.39022261	0
IFITM3	6.375191424	0
AL136295.5	6.321974965	0
STAT1	6.318485897	0
APOL1	6.181818182	0
MDK	6.166666667	0.000232672
HLA-F	6.142608696	1.18275E-12
GMPR	6.118644068	0.007177217
TRIM14	5.944940476	0
TMC4	5.705882353	0.001123378
TLR3	5.69047619	0

CFB	5.679662803	1.3774E-11
REC8	5.608247423	2.26348E-13
SP100	5.498659004	0
STX11	5.279411765	0.000454244
TRIM25	5.206817487	0
EIF2AK2	5.192388463	0
PHF11	4.93	0
C4A	4.839622642	0.000781343
ODF3B	4.790697674	2.93598E-09
PNPT1	4.680120009	0
TYMP	4.665517241	0
MYD88	4.592684321	0
ZNFX1	4.5854419	0
HLA-B	4.375109553	0
NDUFC2-KCTD14	4.344537815	4.91535E-05
NMI	4.283413849	0
NT5C3A	4.124350595	0
TDRD7	4.066702625	0
IL15RA	4.013793103	0.003348967
TMEM229B	4.007751938	6.20633E-05
LAP3	3.987665601	0
PSMB8	3.885613726	4.85849E-11
APOL2	3.832752613	0
LGALS3BP	3.705646417	0
PIK3AP1	3.678220682	0
TREX1	3.670807453	0.000212496
CEACAM1	3.655669023	0
MSH5-SAPCD1	3.612244898	9.11478E-07
TRIM5	3.581734273	0
GBP3	3.510409189	0
TAP2	3.445278023	0
OGFR	3.43187251	0
EBF4	3.315789474	9.40377E-05
IFITM2	3.312911726	0
TRIM38	3.269753513	0
CD68	3.254237288	0
SEC16B	3.253658537	0.00056518
HLA-E	3.253474831	0
ID4	3.25	0.001259625
ADAR	3.171290317	0
SIDT1	3.145124717	0.00344928
SP140L	3.117073171	2.37665E-14
HES4	3.093410109	0
AC026954.2	2.96039604	0.000276805
AC134772.2	2.926580922	0
CCDC184	2.891304348	0.000142492
BTN3A1	2.871615721	8.3854E-06
RBM43	2.837237978	0
APOBEC3F	2.793030623	0
ATP10A	2.775112444	0
FAM46A	2.65819378	0

CDK18	2.651898734	0.00093745
AP003419.1	2.63190184	4.87859E-05
CMTR1	2.619252188	0
RNF19B	2.616118421	0
C1R	2.605815637	0
HLA-C	2.52985782	0
TTC39B	2.479591837	1.519E-06
BTN3A3	2.45751634	5.22231E-08
TRIM56	2.455240175	0
KIAA1217	2.451693852	0
MAP2	2.439765347	0
ERAP2	2.409461664	0.001445734
PRKD2	2.397413606	0
SLC25A28	2.373097991	0.000184582
NCOA7	2.342736164	0
CRYBG1	2.328638498	8.66223E-05
CASP7	2.295803184	0
PLA1A	2.289032258	3.18858E-05
HDX	2.281430219	1.78064E-07
TRIM69	2.277456647	7.03313E-05
CTSO	2.27680798	0.000233892
RNF213	2.275578349	0
BLZF1	2.268026831	0
AC104662.2	2.242222222	0.000205347
B2M	2.237654618	0
ATF3	2.237472767	0.003013129
CNP	2.223931453	0
PSME2	2.211211837	4.19946E-05
TNFAIP3	2.176020408	0.000290283
PI4K2B	2.147237489	0
IRF2	2.128803668	9.3759E-10
GTPBP1	2.113073338	0
FBXO6	2.102760736	2.27761E-05
BCO1	2.086868687	0.000550716
ADGRE1	2.085959885	5.18983E-09
PPAN-P2RY11	2.07797271	0.000439813
ZCCHC2	2.068897248	0
PPP1R15A	2.058003889	0
CLDN4	2.050955414	0.001417981
EXT1	2.036312849	0
KLF4	2.006934813	0.000191444
ST20-MTHFS	-2.00877193	0.000389958
CLN8	-2.040118139	6.5275E-14
TMEM110	-2.046961326	3.05698E-08
OSR1	-2.189440994	0.003879026
CDKL2	-2.225	0.002959353
REEP1	-2.2578125	0.009835863
METTL25	-2.331096197	0.000380443
SOGA3	-2.36119403	0.000752454
ZMAT3	-2.380766926	3.62896E-83
GPATCH11	-2.401119403	4.19946E-05

AC138811.2	-3.099526066	8.57801E-05
TNFRSF10D	-3.232675262	1.6543E-69
C15orf65	-4.063157895	0.000932935

9/Ctrl cells
