# Supplementary material for: The R2TP complex regulates paramyxovirus RNA synthesis
Source: PLoS Pathog. 2019 May 23;15(5):e1007749. doi: 10.1371/journal.ppat.1007749 (PMC6532945; doi:10.1371/journal.ppat.1007749)
Supplement: S8 Table — (PDF) [file ppat.1007749.s014.pdf]

S8 Table. List of differentially expressed genes between MuV-infected and uninfected control A549/Ctrl cells

Gene name	Fold Change	FDR
BST2	#DIV/0!	4.26699E-06
RSAD2	#DIV/0!	2.24067E-09
MX1	93.76530612	0.000143035
IFIT1	67.24657534	0
OASL	45.11827957	0
CMPK2	38.90163934	2.97966E-07
IFITM1	31.87096774	0
IFI27	31.66666667	8.80242E-13
IFI6	27.32901554	5.00357E-05
ISG15	25.92763158	0.000248171
CCL5	18.0625	2.29239E-09
DHRS2	16.8490566	1.85916E-05
BATF2	11.37967914	2.66154E-05
HSH2D	11.23809524	0.001642006
IRF7	10.24543947	7.08234E-06
HELZ2	9.603505591	7.49319E-05
DDX58	8.453515809	4.14985E-05
DDX60	7.798165138	0.003827371
IFIH1	7.581270183	0.003806238
PARP9	7.31795302	0
IFIT3	6.968888889	0
LAMP3	6.705882353	0.000268802
OAS1	6.611806224	0
IRF9	6.401036269	0
SP110	6.055627426	0
SAMD9L	5.274509804	0
SAMD9	5.134526854	0
DTX3L	4.454438664	0
OAS3	3.935459662	0
CYGB	3.838709677	0.006053339
TRANK1	3.715051362	0
PARP12	3.606334842	0
HERC6	3.488742449	0
TLR3	3.467532468	1.24642E-08
PLSCR1	3.410065238	0
C19orf66	3.279022403	0
AL136295.5	3.136783734	0.000942232
IFI35	3.094588449	0
PPM1K	3.076923077	3.42776E-05
APOL6	3.030130756	0
PARP14	2.975964226	0.002669955
IFI16	2.967910708	0.000330445
TRIM14	2.960182192	0
IFIT5	2.930765993	0
PLEKHA4	2.840148699	0.000131851
NLRC5	2.741965974	0
USP18	2.64238806	0
SP100	2.629299678	0.003452076
TRIM21	2.592690678	0

TRIM34	2.583690987	0.004703762
EIF2AK2	2.498117982	0
PML	2.294431732	0
TRIM25	2.237018096	0
STAT1	2.232145157	0
RAB4B-EGLN2	2.191011236	7.5225E-09
IFITM3	2.119122257	6.5994E-05
HIST2H2AA3	2.103538663	6.17082E-06
ZNFX1	2.039017583	0
UBE2L6	2.024174202	0
ZNF550	-2.027164686	0.000869653
UBE2W	-2.250820441	1.80659E-08
NICN1	-2.962732919	2.02641E-07
MRPL53	-3.287305122	6.31576E-09
