# Supplementary material for: The R2TP complex regulates paramyxovirus RNA synthesis
Source: PLoS Pathog. 2019 May 23;15(5):e1007749. doi: 10.1371/journal.ppat.1007749 (PMC6532945; doi:10.1371/journal.ppat.1007749)
Supplement: S9 Table — (PDF) [file ppat.1007749.s015.pdf]

S9 Table. List of genes upregulated or downregulated following MuV infection in RPAP3-knockdown A549/S

Gene name	Fold Change (MuV vs Mock)		Fold Change (siRPA3 vs siNC)
	siNC	siRPAP3	
CT45A3	0.19512195	11.4	58.425
HIST1H2AK	0.10526316	6.09090909	57.8636364
HSPB7	0.14285714	6.15789474	43.1052632
LRRC31	0.15238095	5.2	34.125
ICAM1	0.1682243	5.3968254	32.0811287
NRXN2	0.09411765	2.96774194	31.5322581
MEF2B	0.17241379	4.61904762	26.7904762
ZNF860	0.22727273	5.5	24.2
SELPLG	0.58064516	13.1	22.5611111
STPG3	0.25806452	5.8	22.475
OAS2	3.05714286	67.8125	22.1816589
RDH16	0.21276596	4.57894737	21.5210526
ACPP	0.25806452	5.54545455	21.4886364
AC098650.1	0.17021277	3.6	21.15
LINC00854	0.37179487	7.81818182	21.0282132
CORO2B	0.59722222	12.5	20.9302326
BARHL1	0.13513514	2.8	20.72
NCCRP1	0.18518519	3.6	19.44
RET	0.20408163	3.9	19.11
IMPG2	0.16326531	3	18.375
PPP1R32	0.15238095	2.8	18.375
SV2A	0.22727273	4.09090909	18
APOL3	0.66666667	11.9333333	17.9
HDC	0.13559322	2.32258065	17.1290323
APOBR	0.16161616	2.72727273	16.875
CRACR2A	0.08988764	1.46666667	16.3166667
AC068547.1	0.24242424	3.8125	15.7265625
IL1A	0.5106383	8	15.6666667
NKX2-1	0.24390244	3.78947368	15.5368421
CATSPER1	0.18181818	2.78947368	15.3421053
KCNQ4	0.33009709	4.76190476	14.4257703
FOXD4L5	0.77966102	10.6	13.5956522
GAL3ST2	0.48684211	6.27272727	12.8845209
OTOGL	0.21621622	2.76190476	12.7738095
THEG	0.37179487	4.72727273	12.7147335
AC004832.3	0.32947977	4	12.1403509
INCA1	0.44888889	5.42857143	12.0933522
FAM156B	0.46153846	5.49019608	11.8954248
TAS2R5	0.45138889	5.36842105	11.8931174
SDR16C5	0.07920792	0.93589744	11.8157051
TYRP1	0.25806452	2.94736842	11.4210526
CYP2W1	0.83333333	9.5	11.4
CCDC65	0.14159292	1.57894737	11.1513158
CIDEB	0.30588235	3.34482759	10.9350133
RASGRP2	0.43333333	4.72727273	10.9090909
H2BFM	0.64	6.9	10.78125
NDUFC2-KCT	0.46420323	5	10.7711443
AL109811.4	0.36631016	3.92964824	10.7276529

TACR2	0.23684211	2.52380952	10.6560847
AC011448.1	0.30693069	3.25	10.5887097
ALS2CR12	0.43243243	4.54545455	10.5113636
SH2D6	0.23684211	2.48275862	10.4827586
GPM6A	0.31818182	3.3	10.3714286
ENDOU	0.16666667	1.72727273	10.3636364
TMEM171	0.38297872	3.83333333	10.0092593
CAMK2N2	0.21052632	2.09375	9.9453125
HCAR2	3.27272727	32.3	9.86944444
ADGRG3	0.28571429	2.8	9.8
SHISA7	0.25806452	2.5	9.6875
ELOVL3	0.25806452	2.47619048	9.5952381
GOLGA8K	0.2625	2.5	9.52380952
MAK	0.18181818	1.7	9.35
TPH1	0.16326531	1.51612903	9.28629032
ATP4A	0.31372549	2.89655172	9.23275862
B3GNT8	0.33050847	3.04761905	9.22100122
CCDC154	0.57407407	5.27272727	9.18475073
NAPSA	0.44285714	4	9.03225806
CABP1	0.74285714	6.7	9.01923077
NANOS3	0.70454545	6.1	8.65806452
C17orf107	0.25806452	2.21052632	8.56578947
NUP210L	0.18181818	1.54545455	8.5
LRRC36	0.2421875	2.04081633	8.42659645
AL022318.4	0.52631579	4.4	8.36
DUSP26	0.36363636	3	8.25
ASPRV1	0.24786325	2.03225806	8.19911012
CD74	0.43333333	3.54545455	8.18181818
TXLNB	0.52631579	4.23809524	8.05238095
HOXC8	0.48507463	3.88235294	8.00361991
PSG9	0.40909091	3.19047619	7.7989418
SERPINB4	0.45588235	3.54545455	7.7771261
LGI4	0.25	1.93333333	7.73333333
ALK	0.22580645	1.71428571	7.59183673
IFIT1	4.59090909	34.3875	7.49034653
FAM184A	0.34065934	2.53333333	7.43655914
AC005697.1	0.67741935	5	7.38095238
COL20A1	0.40789474	3	7.35483871
IL20RB	0.74603175	5.47368421	7.33706607
AC023055.1	0.6212766	4.55	7.32363014
FAM71F2	0.0776699	0.56666667	7.29583333
GSDMC	0.72727273	5.3	7.2875
CRB2	1.14814815	8.36363636	7.28445748
PRKN	0.25	1.7826087	7.13043478
AC002985.1	0.57407407	4.09090909	7.12609971
EYS	0.27586207	1.95238095	7.07738095
MYO16	0.47727273	3.375	7.07142857
KISS1	0.19607843	1.36666667	6.97
KCNIP2	0.51461988	3.57142857	6.93993506
CRABP1	0.40625	2.8	6.89230769
LRGUK	0.28571429	1.96774194	6.88709677

ADORA2A	0.53571429	3.675	6.86
UQCRHL	0.28571429	1.95238095	6.83333333
IGFL4	0.35294118	2.38596491	6.76023392
PLG	0.17821782	1.19047619	6.67989418
TLR6	0.21176471	1.4137931	6.67624521
ICAM2	0.42105263	2.8	6.65
CYP26C1	0.23300971	1.54545455	6.63257576
MSS51	0.69306931	4.54545455	6.55844156
CILP2	0.62962963	4.09090909	6.4973262
CACNA1I	0.73076923	4.72727273	6.46889952
ADGRV1	0.67123288	4.31034483	6.42153413
DCN	0.34482759	2.20408163	6.39183673
AC002310.4	0.88571429	5.63636364	6.36363636
HEY2	0.20118343	1.28	6.36235294
CALHM5	0.65656566	4.16981132	6.3509434
ERV3-1	0.12676056	0.79661017	6.28436911
SLC8A3	0.32	2	6.25
A4GNT	0.10810811	0.66666667	6.16666667
ATOH7	0.45454545	2.8	6.16
GRAP	0.66666667	4.1	6.15
SMIM32	0.66101695	4.05	6.12692308
SCX	0.23076923	1.4137931	6.12643678
ENO4	0.25806452	1.5625	6.0546875
ANKRD34B	0.24390244	1.47368421	6.04210526
IGF1	0.16666667	1	6
C9orf84	0.32	1.9	5.9375
CATSPER3	0.93548387	5.54545455	5.92789969
C4B	0.97297297	5.725	5.88402778
DACT1	1.04255319	6.1	5.85102041
KCTD19	1.3373494	7.81818182	5.84602785
CT45A1	0.32	1.85714286	5.80357143
AP000350.4	0.32506887	1.85207101	5.69747267
CRIP3	0.34558824	1.95238095	5.64944276
RTL9	0.65957447	3.71428571	5.63133641
RIMBP3C	1.58974359	8.90909091	5.60410557
SNAP91	0.35227273	1.97183099	5.5974557
GALNT3	0.33333333	1.85714286	5.57142857
C11orf42	0.72340426	4	5.52941176
AL603832.3	0.775	4.27272727	5.51319648
GPR87	0.14814815	0.80952381	5.46428571
BHMG1	0.31578947	1.725	5.4625
FAM19A2	0.34042553	1.85714286	5.45535714
SPOCK2	0.18823529	1.025	5.4453125
KRT19	0.55172414	3	5.4375
VMO1	0.70731707	3.84	5.42896552
PRSS36	0.20833333	1.125	5.4
CCL5	19.7142857	106.177305	5.38580532
MASP1	0.42105263	2.25	5.34375
CCDC188	0.40909091	2.17647059	5.32026144
AC090360.1	0.17021277	0.9047619	5.31547619
AC091167.8	0.34823091	1.84913793	5.31009128

KIAA1324L	0.40625	2.15625	5.30769231
AKR7L	0.31818182	1.675	5.26428571
PRRX2	0.68421053	3.58064516	5.23325062
CD160	0.78421053	4.0952381	5.22211569
KPNA7	0.87012987	4.52380952	5.19900498
SAP25	0.36363636	1.87096774	5.14516129
LYG2	0.8	4.09090909	5.11363636
CDH12	0.36363636	1.85714286	5.10714286
TINAGL1	0.66908213	3.39215686	5.06986621
AGBL3	0.21969697	1.1097561	5.05130362
HHIPL1	0.17241379	0.86885246	5.03934426
TMX2-CTNND	0.45454545	2.28	5.016
TMSB15B_2	0.2	1	5
DLL4	0.30693069	1.52873563	4.98071932
MYLK3	0.28125	1.4	4.97777778
RPEL1	0.18181818	0.89473684	4.92105263
C2orf88	0.23076923	1.13559322	4.92090395
SMC1B	0.32	1.57142857	4.91071429
ISG15	3.6542811	17.91	4.9011008
PCDHGB5	0.72727273	3.54545455	4.875
TTC23L	0.42105263	2.05263158	4.875
CATSPERE	0.61702128	3	4.86206897
LCA5L	0.41176471	2	4.85714286
TTR	0.88235294	4.27272727	4.84242424
SLC5A9	0.65909091	3.19047619	4.8407225
EDAR	0.45454545	2.2	4.84
PCDHB7	0.27586207	1.33333333	4.83333333
CREB3L3	0.3902439	1.86666667	4.78333333
CSTF2T	0.36904762	1.76237624	4.7754711
BEST1	0.36363636	1.72	4.73
IFFO1	0.55714286	2.59322034	4.65449804
NXNL2	0.42105263	1.95238095	4.63690476
C21orf33	0.73084112	3.38541667	4.63221974
CRB1	0.21621622	1	4.625
DLEU7	0.21621622	1	4.625
LRRC39	0.78723404	3.63157895	4.61308677
OASL	11.7149321	54.009009	4.61027076
ABAT	0.52564103	2.41935484	4.60267506
ZNF354C	0.7027027	3.23333333	4.60128205
AC012254.2	0.88888889	4.08474576	4.59533898
RNASEK-C17	1.11111111	5.04761905	4.54285714
PTGER4	0.83950617	3.8	4.52647059
LSMEM2	0.41052632	1.85714286	4.52380952
KIF1A	0.40659341	1.83606557	4.51572884
PRSS53	0.38297872	1.72727273	4.51010101
FXYD3	0.52631579	2.36842105	4.5
GOLGA8R	0.14285714	0.63333333	4.43333333
AQP1	0.36448598	1.6146789	4.43001647
WDR93	0.22727273	1	4.4
BCL2L14	0.62068966	2.72727273	4.39393939
SLC6A12	3.1	13.6	4.38709677

AC026464.1	0.22857143	1	4.375
DNAH9	0.22857143	1	4.375
TPTE	0.22857143	1	4.375
RASIP1	0.38157895	1.66666667	4.36781609
GJC2	0.26666667	1.16129032	4.35483871
RAD9B	0.37362637	1.61016949	4.30957129
PDE4C	0.53703704	2.30952381	4.30049261
LRG1	1.01694915	4.36666667	4.29388889
PCDH12	0.53125	2.2804878	4.29268293
IFIH1	1.3920068	5.95132013	4.27535276
ZNF648	1	4.27272727	4.27272727
SLC47A2	0.32876712	1.40350877	4.26900585
AP000275.2	0.36363636	1.55035971	4.26348921
AC110275.1	0.25543478	1.08396947	4.24362514
RASGRF1	0.22857143	0.96721311	4.23155738
RUNDC3A	0.21276596	0.9	4.23
BEST4	0.34482759	1.45	4.205
B3GNT6	1	4.2	4.2
CENPVL3	1	4.2	4.2
TF	0.51428571	2.15	4.18055556
DRD4	1.58108108	6.54545455	4.13986014
HERC5	1.5795053	6.5276204	4.13269927
AC022335.1	0.42105263	1.70967742	4.06048387
ELL3	0.185	0.75	4.05405405
RYR3	0.42176871	1.7037037	4.03942652
GPR151	0.37179487	1.5	4.03448276
SAMD13	0.76326531	3.06451613	4.01500776
TXK	0.36363636	1.46	4.015
MSANTD1	0.54166667	2.17241379	4.01061008
FAM131B	2.55555556	0.63716814	0.24932666
SLC37A2	1.04255319	0.25806452	0.24753127
FOXJ1	13.25	3.27272727	0.24699828
PPP4R4	3.46153846	0.85	0.24555556
RNF208	2.96078431	0.72666667	0.24543046
HSPB3	4.1	1	0.24390244
USH2A	1.93617021	0.47142857	0.24348509
AP002990.1	1.52205221	0.36876764	0.24228317
SEMA7A	2.64473684	0.64052288	0.24218775
C7orf61	3.7	0.89473684	0.24182077
ACSBG1	3.45384615	0.83168317	0.24079914
ALDH1L1	4.58333333	1.1	0.24
TRIM54	0.83333333	0.2	0.24
ZNF559	1.21938776	0.29152542	0.23907524
ADH4	0.78082192	0.18656716	0.23893689
FAM9C	3.4	0.80952381	0.23809524
EXD1	0.8	0.19047619	0.23809524
LRP1B	0.8	0.19047619	0.23809524
S100A1	1.77142857	0.42105263	0.237691
TMPRSS5	2.95454545	0.7	0.23692308
PCDHB10	0.67741935	0.16	0.23619048
ANO9	3.1	0.72727273	0.23460411

TICAM2	3.1	0.72727273	0.23460411
TMEM121B	1.17241379	0.275	0.23455882
CDH10	2.1	0.49180328	0.23419204
CST2	1.75	0.40983607	0.23419204
SOWAHA	3.75862069	0.875	0.23279817
ZNF10	0.30693069	0.07142857	0.23271889
RLN2	1.34285714	0.31147541	0.23194977
TMEM140	10.1666667	2.35714286	0.23185012
FGF1	1.48	0.34246575	0.23139578
NRN1L	1.5	0.34693878	0.23129252
IRF8	3.48	0.8	0.22988506
RGPD1	0.83333333	0.19047619	0.22857143
NSMCE3	4.5	1.02222222	0.22716049
ZGLP1	1.44444444	0.32673267	0.22619954
ZNF525	1.24137931	0.27868852	0.22449909
RDH12	3.1	0.69491525	0.22416621
AC068946.1	1.36162362	0.30434783	0.22351832
PEX5L	1.77419355	0.39655172	0.22351097
FP565260.3	2.29411765	0.51170569	0.2230512
AC137834.1	3.15116279	0.7	0.22214022
TTLL9	7.8	1.71428571	0.21978022
SLC44A4	1.19512195	0.26190476	0.2191448
NAT6	1.47706422	0.3236715	0.21913164
SCG5	0.83939394	0.18232044	0.21720486
LRRC26	4.36363636	0.94505495	0.21657509
SH2D7	2	0.43262411	0.21631206
MYH7B	1.20618557	0.25954198	0.21517583
HSPE1-MOB4	4.25490196	0.90675991	0.21310947
ACVR1C	4.1	0.87323944	0.21298523
AGRP	4.7	1	0.21276596
CPA6	1.93617021	0.40983607	0.21167357
SLC6A4	1	0.21052632	0.21052632
MESP2	1.17142857	0.24637681	0.21032167
MROH7-TTC4	4.37837838	0.91935484	0.20997611
IFI44L	3.18181818	0.66666667	0.20952381
F7	1.53030303	0.31884058	0.20835127
OR2C3	1.37692308	0.28462709	0.20671241
AS3MT	1.31124498	0.2640264	0.20135551
WNT11	3.55172414	0.70754717	0.19921231
ACHE	5.41666667	1.07758621	0.19893899
CATIP	2.41666667	0.48076923	0.19893899
PRICKLE4	1.20491803	0.23611111	0.19595616
DCST2	0.70731707	0.1375	0.19439655
HOXA6	2.36842105	0.45901639	0.19380692
BRINP3	7.6	1.47169811	0.19364449
C7orf55-LUC7	3.83823529	0.74166667	0.19323116
IGFBP5	1.24	0.2375	0.19153226
CYP3A7	1	0.19047619	0.19047619
CLEC2B	7.68421053	1.46341463	0.19044437
HOXB2	4.14457831	0.78698225	0.18988234
ART1	2.9	0.55	0.18965517

AC010463.1	1.05714286	0.2	0.18918919
HSPB6	1.4950495	0.28282828	0.18917653
NOS3	2.77419355	0.52380952	0.18881506
BSPRY	1.47727273	0.27868852	0.18865069
GAL3ST4	1.17647059	0.22	0.187
KCNH4	4.63157895	0.86440678	0.18663328
INSL3	1.88235294	0.35	0.1859375
ZFP91	2.98544233	0.55382883	0.18550981
CYP46A1	1.02857143	0.19047619	0.18518519
GDPGP1	4.08208955	0.75384615	0.18467164
ENHO	1.59090909	0.29126214	0.18307906
NETO1	2.1	0.38095238	0.1814059
PCDHA13	2.1	0.37931034	0.18062397
FOXH1	0.87179487	0.15625	0.17922794
CYP7B1	5.5	0.98571429	0.17922078
TBC1D3C	1	0.17460317	0.17460317
ADGRD2	0.94736842	0.16326531	0.1723356
HIST1H2BH	1.62857143	0.27777778	0.1705653
PXT1	2.47368421	0.42105263	0.17021277
AL672142.1	3.08333333	0.52380952	0.16988417
CARMIL2	3.34090909	0.56521739	0.16918072
GPR182	1.84313725	0.31147541	0.16899198
NFATC4	0.64197531	0.10810811	0.16839917
HFM1	1.71428571	0.28813559	0.1680791
CA14	2.83333333	0.475	0.16764706
MUC4	6	1	0.16666667
GPSM3	1.09677419	0.18032787	0.16441659
TBC1D28	4.58333333	0.75	0.16363636
PLEKHS1	5.22727273	0.85148515	0.16289281
ART5	2.56578947	0.41584158	0.16207159
AL136295.3	2.36842105	0.38095238	0.16084656
WDR38	1.19354839	0.19047619	0.15958816
DMBX1	1.1225	0.17886179	0.15934235
TLL2	4.2972973	0.68449198	0.1592843
CCRL2	2.04878049	0.32369942	0.15799615
C1orf54	2.41666667	0.38095238	0.15763547
ADRB2	3.4516129	0.53846154	0.15600288
HTR1B	4.63157895	0.72	0.15545455
C9orf66	1.05714286	0.1641791	0.15530456
AL117348.2	2.93548387	0.4556962	0.15523717
USP44	1.14356436	0.17592593	0.15383999
AL513523.10	1.24	0.19047619	0.15360983
KRTAP3-1	2.74285714	0.42105263	0.15350877
MMP10	3.41666667	0.52380952	0.1533101
FAM13C	2.10416667	0.3220339	0.15304581
DSG4	1.32258065	0.2	0.15121951
UPK3BL1	1.83783784	0.275	0.14963235
CBLN2	3.42105263	0.50847458	0.14863103
SYCE1L	4.82926829	0.71428571	0.14790765
FAM86B2	3.21276596	0.46082949	0.14343699
GALNTL6	1.19512195	0.17117117	0.14322486

CNTN5	11.5	1.64516129	0.1430575
IRF6	1.14285714	0.16326531	0.14285714
COX6B2	3.96774194	0.5631068	0.14192123
AL513165.2	1.17204301	0.16	0.13651376
HIST2H2AA3	1.71557562	0.23242737	0.13548069
PALM3	1.37209302	0.18478261	0.13467207
ASGR1	4.33333333	0.57894737	0.13360324
SCTR	2.73684211	0.36065574	0.13177806
CDHR2	1	0.13114754	0.13114754
CFAP61	4.33333333	0.56666667	0.13076923
SP8	14.1	1.79310345	0.12717046
GLB1L3	1.6	0.2	0.125
CAMK2A	5.7	0.69387755	0.1217329
AC008560.1	2.29310345	0.27868852	0.12153334
TBC1D3H	2.59090909	0.31147541	0.12021858
CD86	4.75	0.56944444	0.11988304
GPR68	2.72	0.32432432	0.11923688
TMEM253	3.9	0.45238095	0.11599512
NPIPA5	7.10344828	0.82068966	0.11553398
SLC52A1	12.5	1.43786982	0.11502959
AL049697.1	2.36842105	0.27027027	0.11411411
ALOX12B	1.703125	0.19191919	0.1126865
ARHGEF33	6.5	0.72727273	0.11188811
BUB1B-PAK6	3.4137931	0.38095238	0.11159211
SEC14L5	2.4	0.25806452	0.10752688
HIGD2B	2.6	0.275	0.10576923
ZNF91	2.06896552	0.21794872	0.10534188
CFAP65	2	0.21052632	0.10526316
AC091167.7	1.32258065	0.1375	0.10396341
GNAT2	2.6	0.26666667	0.1025641
NPY2R	12.3	1.24137931	0.10092515
Z84492.1	2.05263158	0.2	0.0974359
GNG7	1.65306122	0.15942029	0.09643943
AL358075.4	1.38888889	0.13297872	0.09574468
MFSD7	2.5	0.23611111	0.09444444
SLC16A11	6.8	0.62857143	0.09243697
HAS1	2.19354839	0.20238095	0.0922619
AC093227.2	4.75	0.42307692	0.08906883
OLFML2B	2.65853659	0.23170732	0.08715596
FOXP3	4.18181818	0.35643564	0.08523461
ITIH4	4.5	0.37931034	0.08429119
RGMA	6.13636364	0.4950495	0.08067473
TM4SF5	7.2	0.5631068	0.07820928
AC007998.2	4.9	0.38095238	0.07774538
CLDN16	2.6	0.2	0.07692308
SLA2	3.27272727	0.24590164	0.07513661
ZC3H10	4.59090909	0.34	0.07405941
NOTCH4	5.2	0.38095238	0.07326007
ACADL	4.86363636	0.34146341	0.07020743
S100A5	1.94736842	0.13414634	0.06888596
MGAM2	2.16666667	0.14035088	0.06477733

ALKAL1	4.36363636	0.26666667	0.06111111
RBMXL1	13.2	0.8	0.06060606
FAM9B	1.7037037	0.1	0.05869565
HAVCR2	5.16666667	0.27868852	0.05393971
ADIRF	5.21052632	0.27472527	0.05272505
FNDC5	8.9	0.41666667	0.04681648
ZNF695	3.48333333	0.14503817	0.04163775
PCDH20	3.9	0.15740741	0.04036087
NODAL	3.18181818	0.12698413	0.0399093
TUBA8	7.16666667	0.13559322	0.01891998

eV-C cells
