# Supplementary material for: The R2TP complex regulates paramyxovirus RNA synthesis
Source: PLoS Pathog. 2019 May 23;15(5):e1007749. doi: 10.1371/journal.ppat.1007749 (PMC6532945; doi:10.1371/journal.ppat.1007749)
Supplement: S10 Table — (PDF) [file ppat.1007749.s016.pdf]

S10 Table. List of genes upregulated or downregulated following MuV infection in RPAP3-knockdown A549/(

Gene name	Fold Change (MuV vs Mock)		Fold Change (siRPA3 vs siNC)
	siNC	siRPAP3	
GBP4	4.58064516	467.222222	101.999218
NEURL3	0.10638298	9.81818182	92.2909091
IFIT3	6.96888889	579.076923	83.094584
APOL3	0.9	70.5555556	78.3950617
IFIT2	5.91764706	292.882353	49.4930417
C11orf65	0.08653846	3.61904762	41.8201058
AC018512.1	0.28712871	9.22222222	32.1187739
AC011511.4	0.52702703	15	28.4615385
THEMIS2	2.36842105	65.1428571	27.5047619
ZNF830	0.72580645	16	22.0444444
MCTP2	0.3	6.22222222	20.7407407
TICAM2	0.25	4.88888889	19.5555556
ALOX5AP	0.17647059	3.22222222	18.2592593
EYS	0.96774194	17.6666667	18.2555556
TMEM140	2.21428571	39.5	17.8387097
PRSS53	0.16666667	2.90909091	17.4545455
FP565260.3	0.28373702	4.66666667	16.4471545
TACR2	0.21686747	3.52631579	16.2602339
BLNK	0.17307692	2.81034483	16.2375479
CILP2	0.35365854	5.63636364	15.9373041
CLDN2	0.08490566	1.31623932	15.5023742
MX2	8.95238095	134.974359	15.0769231
POU1F1	0.35365854	5.11111111	14.4521073
VTCN1	0.125	1.8	14.4
PTGER4	0.42253521	6	14.2
PRIMA1	0.24390244	3.45454545	14.1636364
CCL28	0.26923077	3.71428571	13.7959184
PPP1R32	0.2	2.75	13.75
IL1A	0.625	8.22222222	13.1555556
ESPNL	0.12676056	1.66666667	13.1481481
UBA7	2.54098361	31.925	12.5640323
TECTA	0.13888889	1.73469388	12.4897959
TMPPE	0.29032258	3.54545455	12.2121212
OAS2	64.5686275	782.172414	12.1138151
PWP2	0.27906977	3.2244898	11.5544218
IFIT1	67.2465753	760.926471	11.315468
RP1L1	0.14516129	1.62921348	11.2234707
OPN1SW	0.36538462	4	10.9473684
AC034102.2	0.74324324	8	10.7636364
RAB7B	0.19607843	2.09375	10.678125
IRF4	1	10.5555556	10.5555556
TMC4	0.54492188	5.70588235	10.4710099
IFITM1	31.8709677	331.05	10.3871964
ZNF420	1.26923077	13.1111111	10.3299663
DERL3	0.37864078	3.84210526	10.1470985
LAMP3	6.70588235	67.9722222	10.1362086
FAM129C	0.42857143	4.33333333	10.1111111
ADAT3	0.50704225	5.11111111	10.0802469

HOXA4	0.1369863	1.375	10.0375
CCER2	0.20430108	2	9.78947368
SPTBN5	0.24390244	2.38095238	9.76190476
GBP1	1.88267148	18.3542169	9.74902794
BHLHA15	0.34615385	3.28571429	9.49206349
SLC2A14	0.31292517	2.94736842	9.4187643
TLR6	0.19230769	1.775	9.23
LAYN	0.10843373	1	9.22222222
HESX1	0.68292683	6.27272727	9.18506494
ST14	0.12676056	1.15730337	9.1298377
ANKRD34B	0.42857143	3.88888889	9.07407407
AKR7L	0.7311828	6.55555556	8.96568627
TMEM130	0.50980392	4.55555556	8.93589744
LRP2	1.01587302	8.88888889	8.75
PLEKHS1	0.17307692	1.5	8.66666667
AL359736.1	0.40797546	3.52941176	8.65103936
SPEF1	0.14516129	1.25	8.61111111
AL669918.1	1.28091603	11.025974	8.60788199
SEMA6A	1.45	12.3636364	8.52664577
PTGES3L	0.12345679	1.05	8.505
MAK	0.85714286	7.27272727	8.48484848
LMO2	4.48387097	37.95	8.46366906
AC005837.2	0.32258065	2.72727273	8.45454545
PIPOX	0.32258065	2.72727273	8.45454545
IL34	0.48148148	4.05263158	8.41700405
GSDMC	0.45238095	3.8	8.4
MSANTD1	0.27777778	2.31578947	8.33684211
HIST1H2AK	0.46774194	3.89473684	8.32667877
AC009779.3	0.69105691	5.75	8.32058824
AC091167.7	0.16666667	1.35714286	8.14285714
AC003002.3	0.14754098	1.17948718	7.99430199
TRIM22	3.65816327	28.8975904	7.89948077
TMPRSS7	0.12676056	1	7.88888889
CPNE9	0.24390244	1.90909091	7.82727273
BEST4	0.59756098	4.66666667	7.80952381
CDK3	0.27878788	2.14516129	7.69460028
PLEKHA4	2.8401487	21.6914286	7.63742708
DCC	0.66666667	5.09090909	7.63636364
AL117348.2	0.775	5.86666667	7.56989247
STX11	0.71568627	5.27941176	7.37671233
TINAG	0.31967213	2.31404959	7.23882178
C2orf70	0.3	2.16666667	7.22222222
FBP2	0.81818182	5.88888889	7.19753086
S1PR2	0.0877193	0.63095238	7.19285714
AL353588.1	1	7.11111111	7.11111111
GRIK2	0.52777778	3.72727273	7.06220096
SOWAHA	0.2173913	1.52631579	7.02105263
TMEM253	0.125	0.87719298	7.01754386
ATP2C2	0.79569892	5.57894737	7.0113798
PTPRO	0.43902439	3.07692308	7.00854701
PLXNA4	0.28846154	2	6.93333333

CD274	1.58064516	10.9414894	6.92216674
KCNV2	2.07317073	14.3333333	6.91372549
DHDH	0.67073171	4.63157895	6.90526316
GOLGA8Q	0.26388889	1.82142857	6.90225564
NLRC4	0.24691358	1.675	6.78375
MINOS1-NBL	0.23885918	1.61983471	6.78154681
CD33	0.23809524	1.6	6.72
APOL6	3.03013076	20.1697391	6.65639233
B3GALT1	0.36538462	2.42105263	6.62603878
HERC5	2.99623494	19.8265896	6.61716788
IGFBP5	0.26548673	1.75	6.59166667
ALKAL2	0.9	5.88888889	6.54320988
LGALS9C	1	6.47368421	6.47368421
C10orf10	0.5177665	3.35	6.47009804
CMPK2	38.9016393	245.369748	6.30743979
NOXRED1	0.12337662	0.7755102	6.28571429
CLIC3	0.27777778	1.74074074	6.26666667
FAM229A	0.37254902	2.33333333	6.26315789
SLC25A34	0.37254902	2.33333333	6.26315789
S1PR4	0.42682927	2.66666667	6.24761905
LZTS1	0.90909091	5.66666667	6.23333333
SMTNL1	5.9047619	36.6363636	6.20454545
TLR2	0.5625	3.45454545	6.14141414
CCDC169	0.225	1.37209302	6.09819121
AL049697.1	0.63709677	3.85	6.04303797
DQX1	0.24390244	1.46774194	6.01774194
MAT1A	0.22891566	1.37078652	5.98817268
CABP1	0.48780488	2.89473684	5.93421053
RASL10A	0.32258065	1.90909091	5.91818182
TRIM15	1.4	8.27272727	5.90909091
SERPING1	2.22580645	13.1333333	5.90048309
FSBP	0.36121673	2.12222222	5.87520468
FAM57B	0.34951456	2.05263158	5.87280702
LHX6	0.3452381	2.02631579	5.86932849
ISG20	1.50563204	8.83655536	5.86900061
SPON2	0.97435897	5.7	5.85
AMN	0.87368421	5.09090909	5.82694414
ELAVL2	0.9	5.2	5.77777778
TMEM229B	0.69565217	4.00775194	5.76114341
GPM6A	0.61290323	3.52631579	5.7534626
AC011448.1	0.29015544	1.66666667	5.74404762
NDUFC2-KCT	0.7592068	4.34453782	5.72246958
LGALS9	1.99484536	11.3941176	5.71177991
C5orf56	1.91891892	10.8813559	5.67056577
SFTPB	0.56862745	3.22222222	5.66666667
SLC15A2	1.52439024	8.55555556	5.61244444
KCNIP3	0.45098039	2.50574713	5.55622189
ALOX12	0.3	1.66666667	5.55555556
CX3CL1	2.24193548	12.3684211	5.51684968
CCL5	18.0625	99.0897436	5.48593736
TTC23L	0.5	2.72727273	5.45454545

SLC5A5	0.59375	3.22222222	5.42690058
FAM109B	0.90909091	4.88888889	5.37777778
AL603832.3	0.37254902	2	5.36842105
KCTD16	0.69918699	3.75	5.36337209
GLT8D2	0.45238095	2.42105263	5.35180055
ISG15	25.9276316	138.627907	5.34672465
CD86	0.29032258	1.55	5.33888889
HSPA6	0.55555556	2.95744681	5.32340426
PTPRN	0.47619048	2.53333333	5.32
RABGEF1_2	0.70212766	3.73333333	5.31717172
HRASLS2	1	5.3125	5.3125
CFAP221	0.79518072	4.22222222	5.30976431
ST6GALNAC1	0.27272727	1.44067797	5.28248588
TEX19	0.38754325	2.03225806	5.24395161
SCG2	0.42741935	2.24	5.24075472
FERMT3	0.5	2.56666667	5.13333333
SOX8	0.3125	1.60185185	5.12592593
CDH9	1	5.11111111	5.11111111
FAM122C	1.11805556	5.68333333	5.08322981
TRPV2	0.36538462	1.85714286	5.08270677
UPK3BL1	0.61290323	3.10526316	5.06648199
ZNF426	0.72864322	3.66666667	5.03218391
CRABP2	0.57633588	2.89830508	5.02884723
WDR86	1.30952381	6.55555556	5.00606061
PGR	0.67391304	3.36842105	4.99830221
IL9R	0.37790698	1.88333333	4.98358974
HS3ST6	0.62647059	3.11290323	4.96895351
SP8	0.41044776	2.02040816	4.92244898
ZNF540	0.225	1.10526316	4.9122807
IZUMO4	0.32258065	1.57894737	4.89473684
NLRC5	2.74196597	13.3844282	4.88132543
ART1	0.46341463	2.23333333	4.81929825
GRM1	0.15322581	0.73333333	4.78596491
BHMG1	0.55769231	2.66666667	4.7816092
MYLK4	0.65972222	3.14285714	4.76390977
RARRES3	1.33766234	6.35714286	4.75242718
HMX1	0.48507463	2.30337079	4.74848747
RNF39	0.56016598	2.65441176	4.73861656
MRPL53	0.30420054	1.43969849	4.73272823
HSD11B2	0.32098765	1.50980392	4.70361991
TNFSF10	3.39111111	15.9128065	4.6925052
C17orf105	0.21428571	1	4.66666667
CALHM3	0.21428571	1	4.66666667
LDLRAD2	0.42857143	2	4.66666667
LRRTM4	0.42857143	2	4.66666667
RBPJL	0.21428571	1	4.66666667
USP18	2.64238806	12.2943213	4.65273119
CHRNA10	0.58510638	2.71428571	4.63896104
CLDN16	0.61290323	2.80952381	4.5839599
IYD	0.42857143	1.95238095	4.55555556
USP44	1.07638889	4.86538462	4.52009926

MDK	1.39215686	6.16666667	4.42957746
CDHR4	0.29032258	1.28571429	4.42857143
CRB2	0.9047619	4	4.42105263
HELZ2	9.60350559	42.3089934	4.40557805
CCDC38	0.42857143	1.87804878	4.38211382
IRF1	1.46718803	6.39022261	4.35542171
RLN2	0.81818182	3.55555556	4.34567901
AASS	0.83185841	3.60784314	4.33708803
USH2A	2.05660377	8.88888889	4.32212029
KCNE4	0.4375	1.89	4.32
UPB1	0.41935484	1.80952381	4.31501832
RBP7	0.8852459	3.81818182	4.31313131
ADGRD1	0.52777778	2.25581395	4.27417381
RGPD4	0.19607843	0.83673469	4.26734694
PAQR8	0.17073171	0.72727273	4.25974026
ISM1	0.21052632	0.89393939	4.24621212
PSMB9	1.70864198	7.25472888	4.24590346
MTTP	0.46341463	1.96666667	4.24385965
GAS7	0.44578313	1.89171975	4.24358754
AC005833.2	1.06451613	4.48979592	4.21768707
MSH5-SAPCD	0.85741627	3.6122449	4.21294187
HRASLS	0.4	1.68292683	4.20731707
PSG9	0.4516129	1.9	4.20714286
LRG1	1.01470588	4.26530612	4.20349009
SAMD9L	5.2745098	21.9622642	4.16384934
C19orf57	0.77419355	3.22222222	4.16203704
AC068547.1	0.28125	1.16666667	4.14814815
MX1	93.7653061	388.038194	4.13839842
PSG1	0.2195122	0.9	4.1
FAM153B	0.64516129	2.63636364	4.08636364
MED26	0.55952381	2.27522936	4.06636736
FUT4	0.36567164	1.48623853	4.06440741
NGFR	1.85714286	7.54545455	4.06293706
TMEM86A	0.62765957	2.5483871	4.06014215
TBX6	0.91558442	3.71428571	4.05673759
IFI35	3.09458845	12.5054294	4.04106382
AL080251.1	2.6557377	10.7272727	4.03928171
AVPR1A	0.95408163	3.84615385	4.03126285
ARHGAP20	1.03478261	4.1627907	4.02286496
HFM1	1.33333333	5.36363636	4.02272727
IRF7	10.2454395	41.131295	4.01459548
SCEL	2.29761905	0.57086614	0.24845988
AC068790.8	0.90909091	0.225	0.2475
RNF224	1.59756098	0.39506173	0.24729055
C4orf19	1.15686275	0.28571429	0.24697337
CATSPERE	0.70422535	0.17355372	0.24644628
CPA4	1.21126761	0.29705882	0.24524624
ACSBG1	1.26548673	0.31034483	0.24523752
HAVCR2	3.95238095	0.96666667	0.24457831
CDKN1C	1.93442623	0.47280335	0.24441529
CLCNKA	1.58064516	0.38461538	0.2433281

HRG	2.36363636	0.56862745	0.24057315
SYNGR4	2.07920792	0.5	0.24047619
GPSM3	1.26595745	0.30337079	0.23963743
CYGB	3.83870968	0.91881919	0.23935626
MMP25	5.55	1.32773109	0.23923083
WNT5B	0.91803279	0.2195122	0.2391115
PCDHGC3	1.93442623	0.4609375	0.23828125
SCN4A	1.8	0.42857143	0.23809524
C5orf49	1.28571429	0.3	0.23333333
MYZAP	2.61538462	0.60869565	0.23273657
KCNA7	10.0909091	2.34693878	0.23257952
HOXA5	3	0.69747899	0.232493
GIPC2	1	0.23076923	0.23076923
TCF21	1.91666667	0.43918919	0.22914219
CDHR1	2.8	0.63888889	0.2281746
KCNC1	2.54545455	0.57894737	0.22744361
PXDNL	1.30769231	0.29487179	0.2254902
CCDC154	2.57142857	0.57894737	0.2251462
HSPB2-C11or	1.33333333	0.3	0.225
INSL3	2.25	0.50617284	0.22496571
DOLK	1.76923077	0.39792388	0.22491349
DHRS9	1.86666667	0.4173913	0.22360248
CYP26C1	2.9	0.64285714	0.22167488
GAL3ST4	4.54545455	1	0.22
MYH11	2.45	0.53846154	0.21978022
SIRT4	3.3902439	0.73333333	0.21630695
ZBED1	2.3164557	0.49817518	0.21505923
SCN5A	1.5	0.32258065	0.21505376
PBX4	1.66666667	0.35802469	0.21481481
PCDHA3	3.16129032	0.66473988	0.21027486
TAT	3.9	0.81944444	0.21011396
STON1	2.70992366	0.56796117	0.20958567
LIPI	4.8	1	0.20833333
LRRC66	1.63461538	0.33884298	0.20729217
SLC26A10	2.57692308	0.53333333	0.20696517
SMIM11A	2.09349593	0.4317032	0.20621163
ALB	2.38095238	0.48780488	0.20487805
TSPAN11	3.95121951	0.8	0.20246914
COLQ	1.98387097	0.4015748	0.20241982
C15orf65	1.22131148	0.24611399	0.20151615
RASL12	1.4516129	0.29166667	0.20092593
FRMPD3	2.63636364	0.52941176	0.20081136
ELOVL3	3.8	0.75	0.19736842
C8orf46	2	0.39285714	0.19642857
GJC2	6	1.17142857	0.1952381
MEF2C	4.9	0.95238095	0.19436346
LIPT1	0.83333333	0.16176471	0.19411765
UBE2Q2L	2.31147541	0.4469697	0.19336987
AC233724.16	2.45	0.47368421	0.19334049
TMEM215	1.9	0.36734694	0.19334049
TMEM217	1.9	0.36666667	0.19298246

BEST1	1.71428571	0.3253012	0.18975904
SKOR1	4.8	0.9	0.1875
PRH1	4.61290323	0.86440678	0.18738888
SLC16A8	2.34146341	0.43678161	0.18654215
AL162231.3	0.93333333	0.17307692	0.18543956
FOXE3	5.4	1	0.18518519
APLN	0.68292683	0.12643678	0.18513957
GFI1	1.63636364	0.3	0.18333333
CA14	5.5	1	0.18181818
NINJ2	2.09677419	0.38	0.18123077
TMEM178A	3.8	0.66666667	0.1754386
ADORA2A	2.49019608	0.43548387	0.17487935
SH2D2A	1.66019417	0.29	0.17467836
AFF3	0.89189189	0.15517241	0.17398119
KCP	1.85714286	0.32142857	0.17307692
PTPRE	1.67231638	0.28888889	0.17274775
USP41	3.96078431	0.68372093	0.17262261
KRBA2	1.60895522	0.27640156	0.17178947
FOXD4L4	2.72580645	0.46774194	0.17159763
LCT	4.04761905	0.69	0.17047059
VAMP5	5.6	0.95238095	0.17006803
PLGLB1	2.14285714	0.36428571	0.17
RASIP1	1.78571429	0.3	0.168
TNFSF4	1.72727273	0.28947368	0.16759003
GP6	2.35616438	0.39473684	0.16753366
MUC4	2.19047619	0.36666667	0.1673913
PCDHB9	3.0952381	0.51470588	0.16628959
ARC	5.3	0.87603306	0.16528926
AC243967.1	1.66666667	0.275	0.165
ACRBP	1.81818182	0.3	0.165
CLDN18	10.1818182	1.67346939	0.1643586
CLDN7	1.56146179	0.256	0.16394894
B3GNT6	1.16129032	0.18965517	0.16331418
ERC2	2	0.32142857	0.16071429
LENEP	2.28571429	0.36666667	0.16041667
SCTR	5.4	0.86111111	0.15946502
ADGRF3	2.33333333	0.37037037	0.15873016
CYTH4	2.17073171	0.34375	0.15835674
PDE6B	1.81818182	0.28205128	0.15512821
TFAP2E	2.06451613	0.31914894	0.15458777
PPP4R4	5.9	0.90697674	0.15372487
NPY2R	1.19512195	0.18367347	0.15368596
ZNF671	1.9	0.28947368	0.15235457
SLC5A2	1.6875	0.25641026	0.15194682
MTRNR2L11	2.37717122	0.35959222	0.15126896
CXorf58	0.85714286	0.12857143	0.15
ZDHHC11B	4.29032258	0.63829787	0.1487762
TRIM74	3.54545455	0.52380952	0.14774115
AC012254.2	4.14754098	0.60429448	0.14569946
FKBP1C	5.3	0.76851852	0.14500349
PRKG1	3.71428571	0.53246753	0.14335664

PRSS22	2.04878049	0.29032258	0.14170507
HTR6	3.9	0.55	0.14102564
LAMB4	3	0.42196532	0.14065511
CACNA2D2	5	0.7	0.14
CCL24	2.63636364	0.36666667	0.13908046
CYBB	6.81818182	0.94736842	0.13894737
C7orf55-LUC7	3.29508197	0.45783133	0.13894384
MFNG	1.8	0.25	0.13888889
DNAH2	2	0.275	0.1375
LINGO2	1.11956522	0.15384615	0.13741598
CYP39A1	5	0.67889908	0.13577982
ZNF879	3	0.40384615	0.13461538
CYP4F2	6.72727273	0.89795918	0.13348042
SNAI2	1.68292683	0.2244898	0.13339249
DUOX1	3.9375	0.525	0.13333333
OVOL3	1.38095238	0.18367347	0.13300493
DACT3	3.4	0.45	0.13235294
SUSD3	2.70967742	0.35135135	0.12966538
CSTA	1.66666667	0.21568627	0.12941176
ACPP	0.90909091	0.11538462	0.12692308
KCNS1	4.36363636	0.55	0.12604167
SHBG	1.8	0.225	0.125
ST8SIA1	1	0.12328767	0.12328767
C2orf88	1.80769231	0.22222222	0.12293144
ADGRF2	2.63636364	0.32142857	0.12192118
ZNF597	0.97761194	0.11702128	0.11970115
LAT	3.49019608	0.41176471	0.11797753
AC083841.5	5.36363636	0.63235294	0.11789631
AC135586.2	2.42622951	0.27350427	0.11272811
PRR26	2.36363636	0.25581395	0.10822898
EBF3	2.6	0.28125	0.10817308
CARD14	6.3	0.66666667	0.10582011
FBXL13	8.36363636	0.88461538	0.10576923
RGMA	7.55	0.79591837	0.10541965
GPR151	1.74509804	0.18333333	0.10505618
PYGM	3.8	0.39285714	0.10338346
CBLN2	2.95	0.3	0.10169492
CATSPERB	6.90909091	0.67226891	0.09730208
AC245748.1	3.0952381	0.3	0.09692308
AC006254.1	1.16901408	0.1122449	0.09601672
HGF	4.36363636	0.40816327	0.09353741
SCX	4.36363636	0.40816327	0.09353741
CYTIP	14.4545455	1.35087719	0.09345691
TBC1D3I	1.33333333	0.12345679	0.09259259
DNAJC28	6.57142857	0.6	0.09130435
COL22A1	8	0.65306122	0.08163265
CNGB1	1.60784314	0.12857143	0.07996516
SAP25	5	0.39285714	0.07857143
C2	5.90909091	0.46315789	0.07838057
C16orf46	3.58064516	0.26351351	0.07359386
AANAT	2	0.14516129	0.07258065

AL713999.1	5.12903226	0.37096774	0.07232704
LRRN4	8.36363636	0.54716981	0.06542248
GYS2	2.63636364	0.16513761	0.06263841
CT45A5	1.85365854	0.11111111	0.05994152
RSPH1	3.25	0.18556701	0.05709754
SLC52A1	9.27272727	0.47211896	0.05091479
C11orf42	4.23809524	0.19298246	0.04553519
IL21R	4.9	0.2195122	0.04479841
AQP1	8.2	0.36521739	0.04453871
HAPLN2	11.2	0.42857143	0.03826531
NEXMIF	6.5	0.24358974	0.03747535

Ctrl cells
